# An application of slow feature analysis to the genetic sequences of coronaviruses and influenza viruses

**DOI:** 10.1186/s40246-021-00327-2

**Published:** 2021-05-07

**Authors:** Anastasios A. Tsonis, Geli Wang, Lvyi Zhang, Wenxu Lu, Aristotle Kayafas, Katia Del Rio-Tsonis

**Affiliations:** 1 Department of Mathematical Sciences, Atmospheric Sciences Group, University of Wisconsin-Milwaukee, Milwaukee, WI 53201 USA; 2 Hydrologic Research Center, San Diego, CA 92127 USA; 3 Key Laboratory of Middle Atmosphere and Global Environment Observation (LAGEO), Institute of Atmospheric Physics, Chinese Academy of Sciences, Beijing, 100029 China; 4 Department of Biology and Center for Visual Sciences, Miami University, Oxford, OH 45056 USA

**Keywords:** Nucleotide complexity, Slow feature analysis, Coronaviruses, Influenza viruses

## Abstract

**Background:**

Mathematical approaches have been for decades used to probe the structure of nucleotide sequences. This has led to the development of Bioinformatics. In this exploratory work, a novel mathematical method is applied to probe the genetic structure of two related viral families: those of coronaviruses and those of influenza viruses. The coronaviruses are SARS-CoV-2, SARS-CoV-1, and MERS. The influenza viruses include H1N1-1918, H1N1-2009, H2N2-1957, and H3N2-1968.

**Methods:**

The mathematical method used is the slow feature analysis (SFA), a rather new but promising method to delineate complex structure in nucleotide sequences.

**Results:**

The analysis indicates that the nucleotide sequences exhibit an elaborate and convoluted structure akin to complex networks. We define a measure of complexity and show that each nucleotide sequence exhibits a certain degree of complexity within itself, while at the same time there exists complex inter-relationships between the sequences within a family and between the two families. From these relationships, we find evidence, especially for the coronavirus family, that increasing complexity in a sequence is associated with higher transmission rate but with lower mortality.

**Conclusions:**

The complexity measure defined here may hold a promise and could become a useful tool in the prediction of transmission and mortality rates in future new viral strains.

**Supplementary Information:**

The online version contains supplementary material available at 10.1186/s40246-021-00327-2.

## Background

Since the early 1970s, scientists have attempted to discover some kind of order or hidden structures in nucleotide sequences. With the advent of sequencing techniques in the late 1970s, scientists had the opportunity to probe nucleic acid sequences for such order [1–3]. Soon, mathematical approaches were employed to shed light in this endeavor, leading to the full-blown field of Bioinformatics [4–7]. We report, for the first time, the application of slow feature analysis (SFA) to genetic sequences. SFA is a procedure for extracting slowly varying, driving signals from a given nonstationary time series and is used here to delineate signals or structure in nucleotide sequences, which would not otherwise be detected. Descriptions of this procedure, which have been successfully applied in many scientific areas, have been reported previously in detail [8–10].

## Methods

SFA is an approach that is designed to optimally identify low-frequency behavior in a time series, thereby delineating its complex structure more effectively. This analysis is rooted, theoretically, in the time-embedding theorems. In this method, a one-dimensional time series is embedded in a multi-dimensional space consisting of the original time series and lagged copies thereof. SFA further uses a nonlinear expansion to map this multi-dimensional input signal onto an even larger feature space, and then solves a linear problem to find a linear combination of feature-space variables that minimizes their time derivative (rate of change) [11]. The objective of SFA is to find the optimally filtered signals that vary as slowly as possible but still carry significant information. To ensure this, the output signals need to be uncorrelated and have unit variance [12]. This approach has been successfully applied in many areas, including climate science [13, 14].

In mathematical terms [8], the goal of SFA is, given an *n*-dimensional input signal **x**(*t*), to find a set of real-valued input-output functions *g*_j_(**x**) such that the output signals

$$ {y}_{\mathrm{j}}\left(\mathrm{t}\right):= {g}_{\mathrm{j}}\left(\mathbf{x}\left(\mathrm{t}\right)\right) $$

minimize $$ \varDelta \left({y}_i\right):= <{\dot{y}}_j^2{>}_t $$

under the constraints

$$ {\displaystyle \begin{array}{cc}<{y}_{\mathrm{j}}{>}_{\mathrm{t}}=0& \left(\mathrm{zero}\ \mathrm{mean}\right),\\ {}<{y^2}_{\mathrm{j}}{>}_{\mathrm{t}}=1& \left(\mathrm{unit}\ \mathrm{variance}\right),\\ {}\forall \mathrm{i}<\mathrm{j}:<{y}_{\mathrm{i}}{y}_{\mathrm{j}}{>}_{\mathrm{t}}=0& \left(\mathrm{decorrelation}\ \mathrm{and}\ \mathrm{order}\right)\end{array}} $$

with <∙>_t_ and $\dot{y}$ indicating temporal averaging and the derivative of *y*, respectively.

The *Δ*-value is a measure of the temporal slowness of the signal *y*(*t*)*.* It is given by the mean square of the signal’s time derivative. Small *Δ*-values correspond to slowly varying signals. The first two constraints avoid the trivial constant solution, while the last constraint guarantees that the output functions *g*_j_ are distinct and hence extract different information from the input signal. For a tutorial on this method, the reader could consult reference [8] or a more recent presentation in [15]. In that tutorial, a simple example of a two-dimensional input signal *x*_1_(*t*)=sin(*t*)+cos(11*t*)^2^ and *x*_2_(*t*)=cos(11*t*) is considered. Both components are quickly varying, but hidden in the signal is the slowly varying “feature” *y*(*t*)=*x*_1_(*t*)−*x*_2_(*t*)^2^=sin(*t*), which can be extracted with a polynomial of degree two, namely *h*(**x**)=*x*_1_−*x*_2_^2^.

In the situation with one observable (time series of some variable) from an unknown system where the actual state space is not known (as is the case here), embedding is necessary (and essential) to delineate the underlying dynamics much like in attractor reconstructions. The SFA algorithm can be summarized as follows. Consider a time series ${\left\{x(t)\right\}}_{t={t}_1,\dots, {t}_n}$, where *t* denotes time and *n* indicates the length of the time series. First, we embed {*x*(*t*)} into an *m*-dimensional state space using time-delayed copies of *x(t):*

$$ \mathbf{X}(t)={\left\{{x}_1(t),{x}_2(t),\dots, {x}_m(t)\right\}}_{t={t}_1,\dots, {t}_N}, $$

where *x*_1_(*t*) = *x*(*t*); *x*_2_(*t*) = *x*_1_(*t* − *τ*); *x*_3_(*t*) = *x*_1_(*t* − 2*τ*), and so on, *τ* is the delay, and *N* = *n* – *m* + 1. Then, nonlinear expansions (usually second-order polynomials) are used to generate a *k*-dimensional function state space:

$$ \mathbf{H}(t)={\left\{{x}_1(t),\dots, {x}_m(t),{x}_1^2(t),\dots, {x}_1(t){x}_m(t),\dots, {x}_{m-1}^2(t),\dots, {x}_m^2(t)\right\}}_{t={t}_1,\dots, {t}_N}, $$

which can also be written as $\mathbf{H}(t)={\left\{{h}_1(t),{h}_2(t),\dots, {h}_k(t)\right\}}_{t={t}_1,\dots, {t}_N}$, where

$$ k=m+m\left(m+1\right)/2. $$

The expanded signal **H**(*t*) is then centered and normalized to zero mean and unit variance. This process is referred to as whitening or sphering. Thus, we have

$$ {\mathbf{H}}^{\prime }(t)={\left\{{h}_1^{\prime }(t),{h}_2^{\prime }(t),\dots, {h}_k^{\prime }(t)\right\}}_{t={t}_1,\dots, {t}_N}, $$

where

$$ \overline{h_j^{\prime }}=0\ \left(\mathrm{zero}\ \mathrm{mean}\right), $$

$$ {h}_j^{\prime }{h_j^{\prime}}^T=1\ \left(\mathrm{unit}\ \mathrm{variance}\right), $$

$$ {h}_j^{\prime }(t)=\left[{h}_j(t)-\overline{h_j}\right]/S $$, and $$ S=\frac{1}{k}\sqrt{\sum_{j=1}^k{\left({h}_j(t)-\overline{h}\right)}^2} $$.

Using the Schmidt algorithm, **H**^′^(*t*) is orthogonized into:

$$ \mathbf{Z}(t)={\left\{{z}_1(t),{z}_2(t),\dots, {z}_k(t)\right\}}_{t={t}_1,\dots, {t}_N}, $$

where the transformed signal matrix **Z** is column orthogonal:

$$ \overline{z_i}(t)=\overline{z_j}(t)=0,\kern0.5em {z}_i^T(t)\bullet {z}_j(t)=0,\kern0.5em {z}_j^T(t)\bullet {z}_j(t)=1, $$

The final step of SFA is to find the set of coefficients (*a*_1_, *a*_2_, …, *a*_*k*_) such that the time series

$$ y(t)={a}_1{z}_1(t)+{a}_2{z}_2(t)+\dots +{a}_k{z}_k(t) $$

varies as slowly as possible. This set is given by the eigenvector **W**_1_ of the time-derivative covariance matrix

$$ \mathbf{B}={\dot{\boldsymbol{Z}}}^T\dot{\boldsymbol{Z}} $$

corresponding to the smallest eigenvalue *λ*_1_. Here

$$ \dot{\boldsymbol{Z}}(t)={\left\{\dot{z_1}(t),\dot{z_2}(t),\dots, \dot{z_k}(t)\right\}}_{t={t}_1,\dots, {t}_N} $$

and

$$ \dot{z_j}\left({t}_i\right)={z}_j\left({t}_{i+1}\right)-{z}_j\left({t}_i\right). $$

Using **W**_1_, the optimally filtered slow-feature signal (also known as a driving force factor, which can be composed of one or more components) can be written as:

1 $$ y(t)=r{\mathbf{W}}_1\bullet \mathbf{Z}\left(\mathrm{t}\right)+\mathrm{c}, $$

where *r* and *c* are constants derived to best match y(*t*) and the original time series *x*(*t*).

Once the optimally filtered (low-frequency) SFA signal has been identified, its significant periodicities can be found from the time-averaged wavelet power spectrum. Wavelet analysis has been widely used to analyze localized structures and spectral properties of time series. For example, [16] provides a detailed description of the wavelet analysis, along with a very useful toolkit to conduct step-by-step wavelet analysis, including a statistical significance test based on the red-noise surrogate data (see http://paos.colorado.edu/research/wavelets/). We here used the Morlet wavelet with the wavenumber set to 4 to match the smoothness of the SFA-derived slow-feature signal, focusing, once again, on the spectral peaks statistically significant at the 5% level. Note also that SFA is applicable to non-stationary data, so no data pre-processing is required.

The combination of the SFA and wavelet analyses we use in the present study has been shown to be more effective in diagnosing low-frequency periodicities in data sets of a limited length than direct spectral analysis methods. Note that the driving force may not necessarily consist of just one component, but several components, which, as we will see below, correspond to forcings or signals at certain time scales. The success of SFA in delineating these slow signals lies in the fact that embedding the time series in high enough dimensions and the subsequent dynamical procedure removes the noise and small-scale features that may obscure or suppress those slow signals, thereby delineating more accurately the complex structure of a sequence.

## Analysis and results

We first analyzed the nucleotide sequences from three viruses from the same family: SARS-CoV-2, SARS-CoV-1, and MERS. Those sequences are approximately 30,450 bases long and part of the now world-infamous coronavirus family. Since a nucleotide sequence is a string of the bases A, T (U in RNA), C, and G, we first transformed it to a time series of integers in the interval [1–4] (i.e., A➔1, T/U ➔2, C ➔3, G ➔4). Here, we need to stress that a time series represents a particular type of process, where some quantity is sampled in time, *t*. A nucleotide sequence is a very similar object, but the “sampling” is over space. In a time series, we are interested in the dependency of observations at different time scales, whereas in nucleotide sequences, we are interested in dependencies in different space scales. As such, the mathematical tools to identify structures in time can in principle be applied to identify structure in space, as long as *t* is thought as a parameter identifying the scale. Transforming a nucleotide sequence into a time series has been used in the past to identify interesting properties in nucleotide sequences (such as the well-known period 3; see [4, 5] and references therein). Note also that the above transformation of A➔1, T/U ➔2, C ➔3, and G ➔4 may, depending on frequency distribution of A, T/U, C, and G in the sequence, result in a nonstationary time series. However, unlike other spectral methods, SFA is not affected by nonstationarity in the data.

**Fig. 1:**
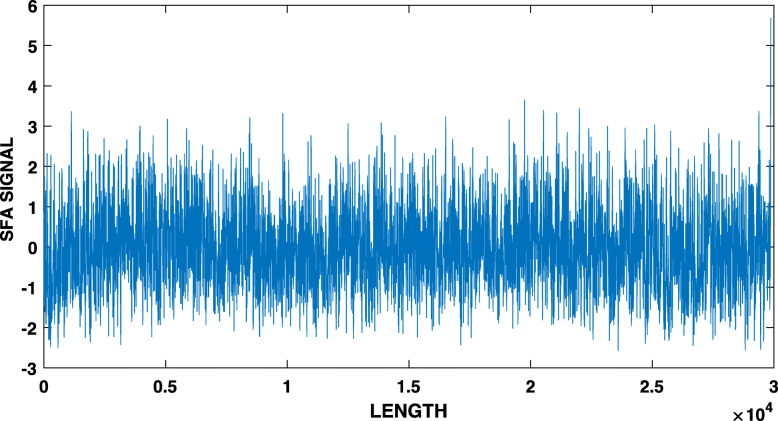
The SFA signal of the nucleotide sequence of SARS-CoV-2. Note the oscillatory components at many scales

**Fig. 2:**
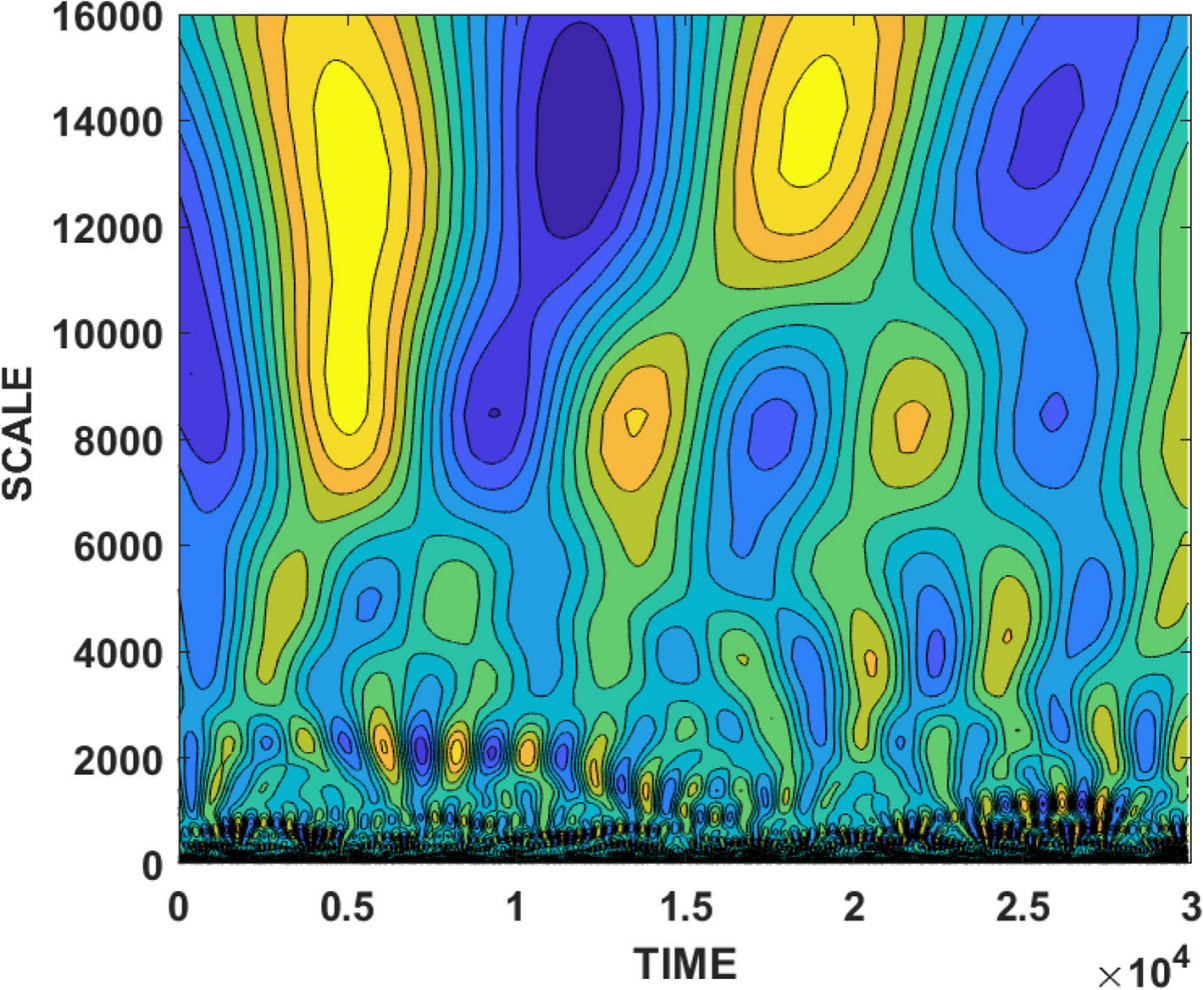
The wavelet of the signal extracted from Fig. 1

**Fig. 3:**
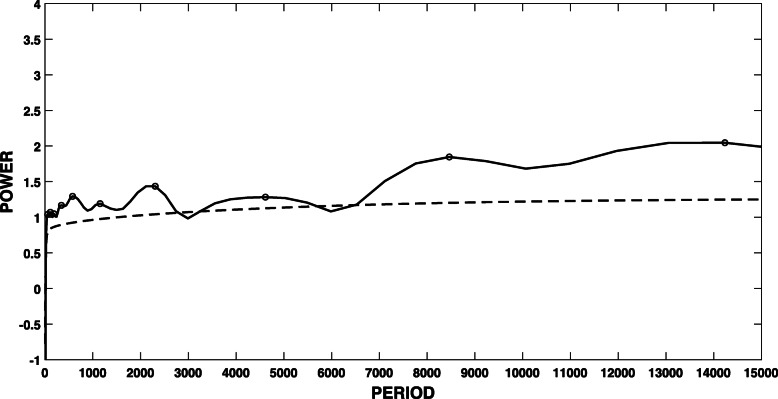
The time-averaged power spectrum of the wavelet transform extracted from Fig. 2. The dashed line represents the 95% confidence level. The dots show the periods of the oscillatory components of the driving force that are significant above the 95% level

Once we have a time series, we apply SFA, and once we have the SFA signal (which as we mentioned above may be comprised of several components, see Eq. 1), we extract the SFA components by wavelet analysis. Figure 1 shows the SFA signal for SARS-CoV-2 virus for *m*=15 and *τ*=1. As explained above, this signal is normalized to zero mean and unit variance. Figure 2 shows the wavelet of the time series in Fig. 1. In order to extract the peak “periods” of the driving force signal, we used the Morlet wavelet to compute the time-averaged power spectrum of the wavelet transform [16]. The black solid line in Fig. 3 is the time-averaged power spectrum of the wavelet transform of the driving force, and the dashed line represents the 95% confidence level, estimated using AR-1 surrogate data [16]. The dots show the periods of the oscillatory components of the driving force that are significant above the 95% level.

The significant peak periodicities for SARS-CoV-2 are as follows^1^:

2 $$ {\displaystyle \begin{array}{c}{P}_1=55.5956928123500\\ {}{P}_2=111.191385624700\\ {}\begin{array}{c}{P}_3=187.000875157807\\ {}{P}_4=342.961117205042\\ {}\begin{array}{c}{P}_5=576.789548058258\\ {}{P}_6=1153.57909611652\\ {}\begin{array}{c}{P}_7=2307.15819223303\\ {}{P}_8=4614.31638446607\\ {}\begin{array}{c}{P}_9=8462.69356236189\\ {}{P}_{10}=14232.4973599616\end{array}\end{array}\end{array}\end{array}\end{array}} $$

Given the above periodicities recovered from SARS-CoV-2, we next construct Table 1, which shows the ratios between these peaks. We observe the following EXACT relations between peak periods:

**Table 1:**
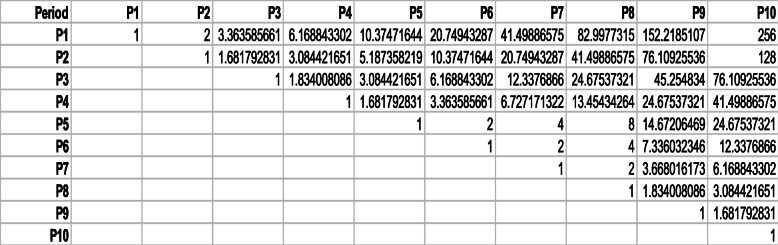
Ratios between the peaks in (2)

3 $$ {\displaystyle \begin{array}{c}{P}_2=2{P}_1\\ {}{P}_{10}=128{P}_2\\ {}\begin{array}{c}{P}_{10}=256{P}_1\\ {}{P}_8=2{P}_7\\ {}\begin{array}{c}{P}_8=4{P}_6\\ {}{P}_8=8{P}_5\\ {}\begin{array}{c}{P}_7=2{P}_6\\ {}{P}_7=4{P}_5\\ {}{P}_6=2{P}_5\end{array}\end{array}\end{array}\end{array}} $$

And the following almost exact relationships based on the criterion:

$$ \mid \mathrm{P}-\mathrm{nearest}\ \mathrm{integer}\mid /\mathrm{nearest}\ \mathrm{integer}<0.25\% $$

4 $$ {\displaystyle \begin{array}{c}{P}_9=152{P}_1\\ {}{P}_9=76{P}_2\\ {}\begin{array}{c}{P}_{10}=76{P}_3\\ {}{P}_8=83{P}_1\end{array}\end{array}} $$

Keeping only those relationships, we remain with Table 2, which could be thought as portraying the degree of structure or complexity in the SARS-CoV-2 sequence. We observe in the exact relationships multiples of a power of 2 and in the almost exact relationships multiples of 19 (152=2×76=8×19) and 83. Clearly, a sophisticated and rather convoluted structure, with numerous processes embedded in the sequence, is present. Keep in mind that the factors 19 and 83 (odd numbers) will appear in the rest of the sequences studied here. We define the number of entries above the diagonal in Table 2 as the degree complexity, *C*. In this case, *C*=13.

**Table 2:**
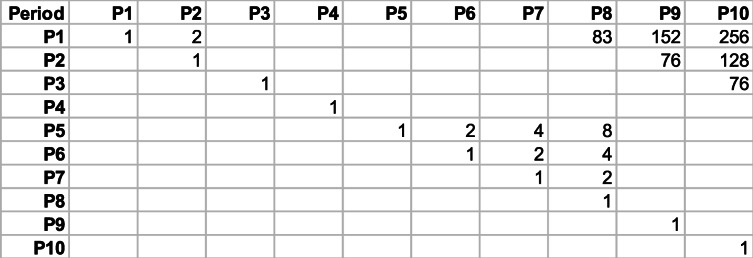
Same as Table 1 but keeping only the exact and almost exact relationships, see relationships (3) and (4)

In the Supplementary material, Figures S1, S2, S3 are similar to Figs. 1, 2, and 3, and Tables ST1 and ST2 are similar to Tables 1 and 2 but for SARS-CoV-1. Figures S4, S5, and S6 are similar to Figs. 1, 2, and 3, and Tables ST3 and ST4 are similar to Tables 1 and 2 but for MERS.

According to Figure S3, the peak periods for SARS-CoV-1 are as follows:

$$ {\displaystyle \begin{array}{c}{P}_1=50.9814750936898\\ {}{P}_2=111.191385624700\\ {}\begin{array}{c}{P}_3=528.918347647618\ \\ {}{P}_4=748.003500631229\\ {}\begin{array}{c}{P}_5=2115.67339059047\\ {}{P}_6=3558.12433999040\ \\ {}\begin{array}{c}{P}_7=8462.69356236189\ \\ {}{P}_8=13051.2576239846\ \end{array}\end{array}\end{array}\end{array}} $$

and according to Tables ST1 and ST2, we now have five exact periodicities

5 $$ {\displaystyle \begin{array}{c}{P}_5=4{P}_3\\ {}{P}_6=32{P}_2\\ {}\begin{array}{c}{P}_7=4{P}_5\\ {}{P}_7=16{P}_3\\ {}{P}_8=256{P}_1\end{array}\end{array}} $$

and three almost exact

6 $$ {\displaystyle \begin{array}{c}{P}_5=19{P}_2\\ {}{P}_7=166{P}_1\\ {}{P}_7=76{P}_2\end{array}} $$

Again here, we observe in the exact relationships, multiples of a power of 2, and in the almost exact between *P*_7_ and *P*_1_, *P*_7_ and *P*_2_, and between *P*_5_ and *P*_2_. Note again the multiples of 19 and 83. Note also that from the almost exact relationships, it follows that *P*_7_/*P*_5_=4, which is one of the exact relationships. Here, the degree of complexity is *C*=8.

According to Figure S6, the peak periods for MERS are as follows:

$$ {\displaystyle \begin{array}{c}{P}_1=101.962950187380\\ {}{P}_2=132.229586911904\\ {}\begin{array}{c}{P}_3=242.510131659000\\ {}{P}_4=628.993462278030\\ {}\begin{array}{c}{P}_5=1779.06216999520\\ {}{P}_6=2515.97384911212\\ {}\begin{array}{c}{P}_7=4614.31638446607\\ {}{P}_8=8462.69356236189\\ {}{P}_9=13051.2576239846\end{array}\end{array}\end{array}\end{array}} $$

and according to Tables ST3 and ST4, we now have three exact periodicities

7 $$ {\displaystyle \begin{array}{c}{P}_9=128{P}_1\\ {}{P}_8=64{P}_2\\ {}{P}_6=4{P}_4\end{array}} $$

and three almost exact relationships

8 $$ {\displaystyle \begin{array}{c}{P}_8=83{P}_1\\ {}{P}_7=19{P}_3\\ {}{P}_6=19{P}_2\end{array}} $$

*P*_9_, *P*_8_, and *P*_6_ are multiples (again in a power of 2) of *P*_1_, *P*_2_, *P*_4_ (ordered in a bottom-top “symmetric” way), *P*_6_, *P*_7_, and *P*_8_ are multiples of *P*_2_, *P*_3_, and *P*_1_ but not of 2, but again of 19 and 83 (it is interesting to note that the odd multiples of 19 and 83 appear in all three sequences). Here, the degree of complexity is *C*=6.

By comparing Tables 2, ST2, and ST4 (and their associated *C*), one may argue that there is more embedded complexity and intricate patterning in SARS-CoV-2 than SARS-CoV-1 and MERS.

### Other important relationships

Keeping in mind that all three sequences belong to the same coronavirus family, there are similarities and inter-relationships between the sequences. For example, it is easy to observe that:

9 $$ {\displaystyle \begin{array}{c}{P}_7\ \left(\mathrm{MERS}\right)\ \mathrm{is}\ \mathrm{the}\ \mathrm{same}\ \mathrm{as}\ {P}_8\ \left(\mathrm{SARS}-\mathrm{CoV}-2\right)\\ {}{P}_8\ \left(\mathrm{MERS}\right)\ \mathrm{is}\ \mathrm{the}\ \mathrm{same}\ \mathrm{as}\ {P}_9\ \left(\mathrm{SARS}-\mathrm{CoV}-2\right)\\ {}\begin{array}{c}{P}_8\ \left(\mathrm{MERS}\right)\ \mathrm{is}\ \mathrm{the}\ \mathrm{same}\ \mathrm{as}\ {P}_7\ \left(\mathrm{SARS}-\mathrm{CoV}-1\right)\\ {}{P}_9\ \left(\mathrm{MERS}\right)\ \mathrm{is}\ \mathrm{the}\ \mathrm{same}\ \mathrm{as}\ {P}_8\ \left(\mathrm{SARS}-\mathrm{CoV}-1\right)\\ {}\begin{array}{c}{P}_2\ \left(\mathrm{SARS}-\mathrm{CoV}-2\right)\ \mathrm{is}\ \mathrm{the}\ \mathrm{same}\ \mathrm{as}\ {P}_2\ \left(\mathrm{SARS}-\mathrm{CoV}-1\right)\\ {}{P}_9\ \left(\mathrm{SARS}-\mathrm{CoV}-2\right)\ \mathrm{is}\ \mathrm{the}\ \mathrm{same}\ \mathrm{as}\ {P}_7\ \left(\mathrm{SARS}-\mathrm{CoV}-1\right)\end{array}\end{array}\end{array}} $$

In general, SFA reveals a consistent picture between these sequences with very intricate structure with details at many scales, indicating very elaborate and sophisticated embedded processes, with complexity increasing from MERS to SARS-CoV-1 to SARS-CoV-2.

### Extension of the analysis to the influenza viruses of H1N1-1918, H1N1-2009, H2N2-1957, and H3N2-1968

In an effort to provide further support for the efficiency and consistency of SFA in the analysis of nucleotide sequences, we consider four other viral sequences from a different viral family, that of the influenza viruses or the *Orthomyxoviridae* family [17].

In the Supplementary material, Figures S7, S8, and S9 and Tables ST5 and ST6 correspond to H1N1-1918 and are similar to Figs. 1, 2, and 3 and Tables 1 and 2. Figures S10, S11, and S12 and Table ST7 and ST8 correspond to H1N1-2009 and are again similar to Figs. 2 and 3 and Table 2. Figures S13, S14, and S15 and Tables ST9 and ST10 correspond to H2N2-1957 and are similar to Figs. 2 and 3 and Table 2. The same goes for Figures S16, S17, and S18 and Tables ST11 and ST12, which correspond to H3N2-1968. From these figures and tables, it follows that:

- Peak SFA periodicities for H1N1-1918

$$ {\displaystyle \begin{array}{c}{P}_1=60.6275329147499\\ {}{P}_2=157.248365569507\\ {}\begin{array}{c}{P}_3=288.394774029129\\ {}{P}_4=576.789548058258\\ {}\begin{array}{c}{P}_5=970.040526635999\\ {}\begin{array}{c}{P}_6=1631.40720299807\\ {}{P}_7=2515.97384911212\\ {}{P}_8=5031.94769822424\end{array}\end{array}\end{array}\end{array}} $$

Exact relationships

10 $$ {\displaystyle \begin{array}{c}{P}_4=2{P}_3\\ {}{P}_5=16{P}_1\\ {}\begin{array}{c}{P}_7=16{P}_2\\ {}{P}_8=32{P}_2\\ {}{P}_8=2{P}_7\end{array}\end{array}} $$

Almost exact relationships

11 $$ {P}_8=83{P}_1 $$

Complexity measure, *C*=6

b) Peak SFA periodicities for H1N1-2009
  $$ {\displaystyle \begin{array}{c}{P}_1=55.5956928123500\\ {}{P}_2=157.248365569507\ \\ {}\begin{array}{c}{P}_3=288.394774029129\ \\ {}{P}_4=576.789548058258\ \\ {}\begin{array}{c}{P}_5=1057.83669529524\ \\ {}\begin{array}{c}{P}_6=2515.97384911212\ \\ {}{P}_7=5984.02800504983\ \end{array}\end{array}\end{array}\end{array}} $$

Exact relationships

12 $$ {\displaystyle \begin{array}{c}{P}_4=2{P}_3\\ {}{P}_6=16{P}_2\end{array}} $$

Almost exact relationships (note 38=2×19)

13 $$ {\displaystyle \begin{array}{c}{P}_5=19{P}_1\\ {}{P}_7=38{P}_2\end{array}} $$

Complexity measure, *C*=4

c) Peak SFA periodicities for H2N2-1957
  $$ {\displaystyle \begin{array}{c}{P}_1=72.0986935072823\\ {}{P}_2=203.925900374759\ \\ {}\begin{array}{c}{P}_3=288.394774029129\ \\ {}{P}_4=576.789548058258\ \\ {}\begin{array}{c}{P}_5=889.531084997600\ \\ {}\begin{array}{c}{P}_6=2515.97384911212\ \\ {}{P}_7=5984.02800504983\ \end{array}\end{array}\end{array}\end{array}} $$

Exact relationships

14 $$ {\displaystyle \begin{array}{c}{P}_3=4{P}_1\\ {}\begin{array}{c}{P}_4=8{P}_1\\ {}{P}_4=2{P}_3\end{array}\end{array}} $$

Almost exact relationships

15 $$ {P}_7=83{P}_1 $$

Complexity measure, *C*=4

d) Peak SFA periodicities for H3N2-1968

$$ {\displaystyle \begin{array}{c}{P}_1=72.0986935072823\\ {}{P}_2=187.000875157807\ \\ {}\begin{array}{c}{P}_3=628.993462278030\ \\ {}{P}_4=889.531084997600\ \\ {}\begin{array}{c}{P}_5=2515.97384911212\ \\ {}{P}_6=5487.37787528068\end{array}\end{array}\end{array}} $$

Exact relationships

16 $$ {P}_5=4{P}_3 $$

Almost exact periodicities

17 $$ {P}_6=76{P}_1\ \left(\mathrm{note}\ 76=2\times 38=4\times 19\right) $$

Complexity measure, *C*=2

### Inter-relationships

As in the case of the coronaviruses, the influenza virus sequence analysis also revealed plenty of inter-relationships as expected, since the four viruses belong to the same family.

18 $$ {\displaystyle \begin{array}{c}{P}_2\left(\mathrm{H}1\mathrm{N}1-1918\right)={P}_2\left(\mathrm{H}1\mathrm{N}1-2009\right)\\ {}{P}_3\left(\mathrm{H}1\mathrm{N}1-1918\right)={P}_3\left(\mathrm{H}1\mathrm{N}1-2009\right)={P}_3\left(\mathrm{H}2\mathrm{N}2-1957\right)\\ {}\begin{array}{c}{P}_4\left(\mathrm{H}1\mathrm{N}1-1918\right)={P}_4\left(\mathrm{H}1\mathrm{N}1-2009\right)={P}_4\left(\mathrm{H}2\mathrm{N}2-1957\right)\\ {}{P}_7\left(\mathrm{H}1\mathrm{N}1-1918\right)={P}_6\left(\mathrm{H}1\mathrm{N}1-2009\right)={P}_6\left(\mathrm{H}2\mathrm{N}2-1957\right)={P}_5\left(\mathrm{H}3\mathrm{N}2-1968\right)\\ {}\begin{array}{c}{P}_1\left(\mathrm{H}2\mathrm{N}2-1957\right)={P}_1\left(\mathrm{H}3\mathrm{N}2-1968\right)\\ {}{P}_7\left(\mathrm{H}1\mathrm{N}1-2009\right)={P}_7\left(\mathrm{H}2\mathrm{N}2-1957\right)\end{array}\end{array}\end{array}} $$

Interestingly, we found that many relationships exist between the two viral families investigated here. If we compare the results in this section to the previous section, we can infer that:

19 $$ {\displaystyle \begin{array}{c}{P}_1\left(\mathrm{SARS}-\mathrm{CoV}-2\right)={P}_1\left(\mathrm{H}1\mathrm{N}1-2009\right)\\ {}{P}_5\left(\mathrm{SARS}-\mathrm{CoV}-2\right)={P}_4\left(\mathrm{H}1\mathrm{N}1-1918\right)={P}_4\left(\mathrm{H}1\mathrm{N}1-2009\right)={P}_4\left(\mathrm{H}2\mathrm{N}2-1957\right)\\ {}\begin{array}{c}{P}_3\left(\mathrm{SARS}-\mathrm{CoV}-2\right)={P}_2\left(\mathrm{H}3\mathrm{N}2-1968\right)\\ {}{P}_6\left(\mathrm{MERS}\right)={P}_7\left(\mathrm{H}1\mathrm{N}1-1918\right)={P}_6\left(\mathrm{H}1\mathrm{N}1-2009\right)={P}_6\left(\mathrm{H}2\mathrm{N}2-1957\right)={P}_5\left(\mathrm{H}3\mathrm{N}2-1968\right)\\ {}{P}_4\left(\mathrm{MERS}\right)={P}_3\left(\mathrm{H}3\mathrm{N}2-1968\right)\end{array}\end{array}} $$

More on this is discussed next.

## Discussion

**Fig. 4:**
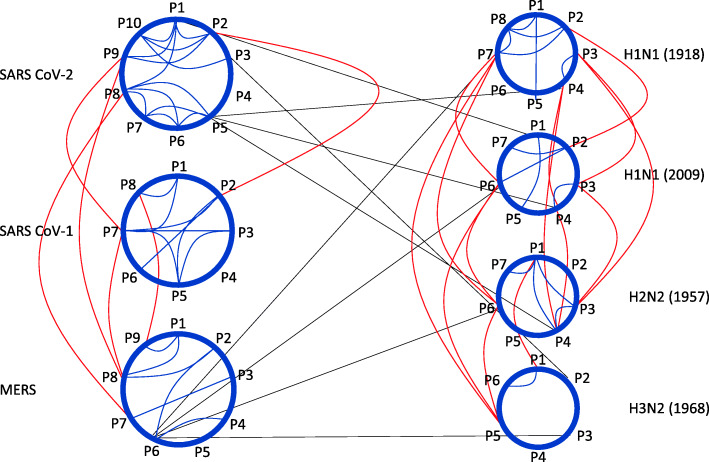
A complex network visualization of the relationships (connections) between individual nucleotide sequences (blue), between sequences within each individual family (the coronavirus family and the influenza family; red), and between the two families (black) resulted from the SFA. This picture is akin to structures of complex networks where in each community the nodes are connected in a certain way (meaning the community obeys its own dynamics), but where there are also connections between the communities

If we consider the peak SFA periodicities from a sequence as nodes of a community, and their relationships as links between the nodes, then, a visualization of the results for the SARS-CoV-2 community would look like the top left panel of Fig. 4. Since there are 10 peak periodicities, we have 10 nodes. Then, from Eqs. 3 and 4, we have 13 (recall that *C*=13) links between them (showing in blue). The rest of the panels correspond to the rest of the sequences in both families. The red lines give the links between the communities within a family (from Eq. 9 for the coronavirus family and from Eq. 18 for the influenza family). The black lines are the links between the two families (Eq. 19). This picture is a perfect example of complex networks, which are often characterized by a community structure, where in each community the nodes are connected in a certain way (meaning the community obeys its own dynamics), but where there exist also some connections (or interactions) between the communities (see for example [18, 19]). We note two interesting observations: (1) the influenza virus family is much more connected (more red links) than the coronavirus family, possibly indicating that the influenza strains are less mutated than the coronavirus strains, and (2) SARS-CoV-1 has no direct links to the influenza family.

This result supports our claims that SFA has the potential and efficiency to delineate the complex mathematical structure of genetic sequences and that it could become a useful tool in such analyses. We need to stress here that, given the mathematics behind SFA, while we can make direct comparisons of the complexity measure “C” within a certain family (where more or less the number of bases is the same), we cannot compare complexities based on “C” between different families. This is due to the differences in nucleotide length between viral families. The coronavirus family sequence length is approximately 30,450 bases, whereas the influenza family sequence length is approximately 13,500 bases. As such, SFA may “see” longer oscillations in the coronavirus family than in the influenza family. Thus, there will be more entries above the diagonal (in tables such as Table 2), and therefore, higher complexity in the coronavirus family.

Finally, it is interesting to note that the complexity measure “C” in the case of the coronavirus family relates to mortality and severity of symptoms as well as to the rate of transmission. As “C” increases, the transmission rate to humans increases, but mortality rate decreases. It is reported that symptoms of the SARS-CoV-2 are milder than SARS-CoV-1 and MERS; however, the viral transmission rate (from human-to-human) is greater than the other family members. The mortality rate of SARS-CoV-2 is lower (3.4%) than that of SARS-CoV-1 (9.6%) and MERS (35%) [20]. This relationship is not as clear, however, in the case of the influenza virus family. Unfortunately, in this case, the outbreaks span over a century, and the actual numbers are skewed by several factors such as deaths by secondary infection (due to the unavailability of antibiotics), hygiene, lack of experience and lack of proper healthcare, especially in the early outbreaks, and other problems. For example, H1N1-1918 (*C*=6) infected 30% of the planet’s population and H1N1-2009 (*C*=4) infected 10% of the population. This is consistent with “increasing C ➔ higher infection rate”, but it is not consistent with “increasing C➔ less mortality rate”. H1N1-1918 killed about 8% of the infected, whereas H1N1-2009 killed only 0.0025% of the infected [21–28]. But how can we compare the conditions in 1918 and 2009? To complicate comparisons further, there is hardly any reliable data of infection rates for H2N2 and H3N3. In any case, the complexity measure “C” may hold a promise and could become a useful tool in the prediction of transmission and mortality rates in future new viral strains.

## Conclusions

In this exploratory work, a relatively recent mathematical method (SFA) is applied to probe the structure of the nucleotide sequences of two related viral families: those of coronaviruses and those of influenza viruses. The coronaviruses are SARS-CoV-2, SARS-CoV-1, and MERS. The influenza viruses include H1N1-1918, H1N1-2009, H2N2-1957, and H3N2-1968. The analysis indicates that the nucleotide sequences exhibit an elaborate and convoluted structure akin to complex networks. We define a measure of complexity and show that each nucleotide sequence exhibits a certain degree of complexity within itself, while at the same time there exists complex inter-relationships between the sequences within a family and between the two families. From these relationships, we find evidence, especially for the coronavirus family, that increasing complexity in a sequence is associated with higher transmission rate but with lower mortality. As such, the complexity measure defined here may hold a promise and could become a useful tool in the prediction of transmission and mortality rates in future new viral strains.

## Supplementary Information

**Additional file 1.** Supplementary figures and tables

**Additional file 2.** Nucleotide sequences used in the study

## Data Availability

All nucleotide sequences used in this analysis are public domain and can be downloaded from the National Center for Biotechnology Information https://www.ncbi.nlm.nih.gov. For convenience, we have supplied all the nucleotide sequences used here.

## Footnotes

1 Note here that before we applied SFA to actual nucleotide sequences, and in order to test the efficiency of SFA when the time series is a string of integers, we considered artificial sequences of known periodicities. SFA was able to reproduce the known periodicities.
