# Correction to: An application of slow feature analysis to the genetic sequences of coronaviruses and influenza viruses

**DOI:** 10.1186/s40246-021-00334-3

**Published:** 2021-06-02

**Authors:** Anastasios A. Tsonis, Geli Wang, Lvyi Zhang, Wenxu Lu, Aristotle Kayafas, Katia Del Rio-Tsonis

**Affiliations:** 1 Department of Mathematical Sciences, Atmospheric Sciences Group, University of Wisconsin-Milwaukee, Milwaukee, WI 53201 USA; 2 Hydrologic Research Center, San Diego, CA 92127 USA; 3 Key Laboratory of Middle Atmosphere and Global Environment Observation (LAGEO), Institute of Atmospheric Physics, Chinese Academy of Sciences, Beijing, 100029 China; 4 Department of Biology and Center for Visual Sciences, Miami University, Oxford, OH 45056 USA

**Correction to: Hum Genomics 15, 26 (2021)**

**https://doi.org/10.1186/s40246-021-00327-2**

Following publication of the original article [1], the authors would like to change all the word DNA to nucleotide or genetic.

The viruses that we analysed have an RNA genome, however the sequences obtained from the repository (NCBI) are reported as DNA. In this manuscript a neutral term "nucleotide" sequence(s) or “genetic” structure should be used instead of DNA sequences or DNA structure

The original article [1] has been updated.
