# Supplementary material for: An application of slow feature analysis to the genetic sequences of coronaviruses and influenza viruses
Source: Hum Genomics. 2021 May 7;15:26. doi: 10.1186/s40246-021-00327-2 (PMC8103670; doi:10.1186/s40246-021-00327-2)
Supplement: Supplementary file 1 — Additional file 1. Supplementary figures and tables [file 40246_2021_327_MOESM1_ESM.docx]

**Supplementary Material**

**An Application of Slow Feature Analysis to the Genetic Sequences of**

**Coronaviruses and Influenza viruses**

Anastasios A. Tsonis^1,2,^* Geli Wang^3^, Lvyi Zhang^3^, Wenxu Lu^3^, Aristotle Kayafas^4^, and Katia Del Rio-Tsonis^4^*

1 Department of Mathematical Sciences, Atmospheric Sciences Group, University of Wisconsin-Milwaukee, Milwaukee, WI 53201, USA

2 Hydrologic Research Center, San Diego, CA 92127, USA

^3^ Key Laboratory of Middle Atmosphere and Global Environment Observation (LAGEO), Institute of Atmospheric Physics, Chinese Academy of Sciences, Beijing 100029, China

^4^Department of Biology and Center for Visual Sciences at Miami University, Oxford, OH 45056, USA

* Corresponding authors


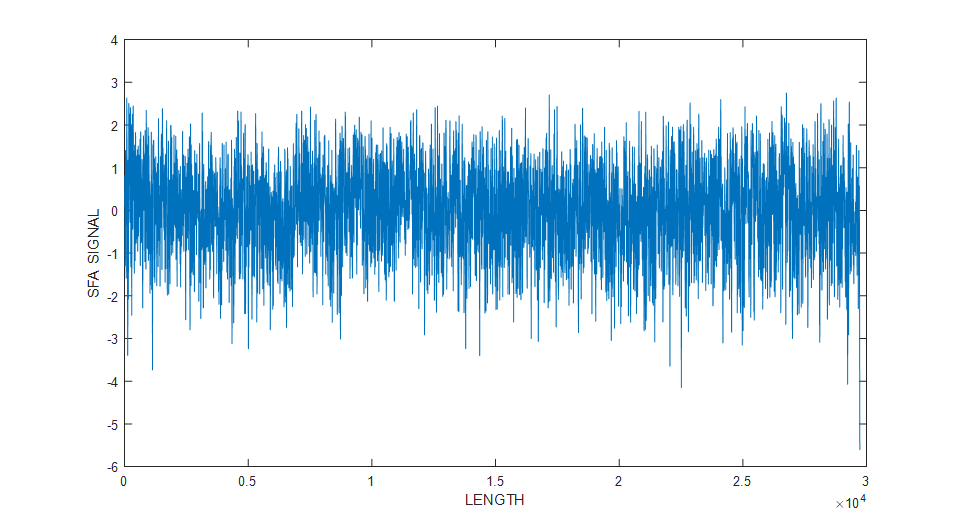


*Figure S1: Same as Figure 1 but for SARS-CoV-1*


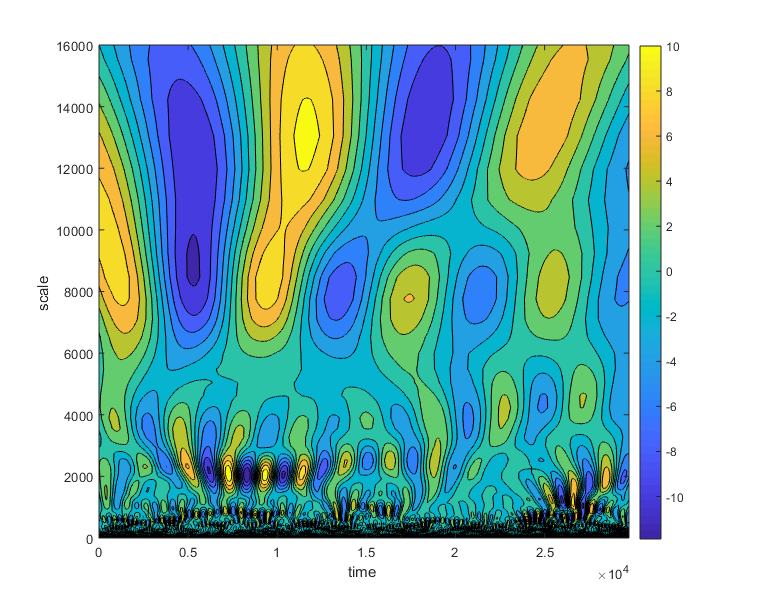


*Figure S2: Same as Figure 2 but for SARS-CoV-1*


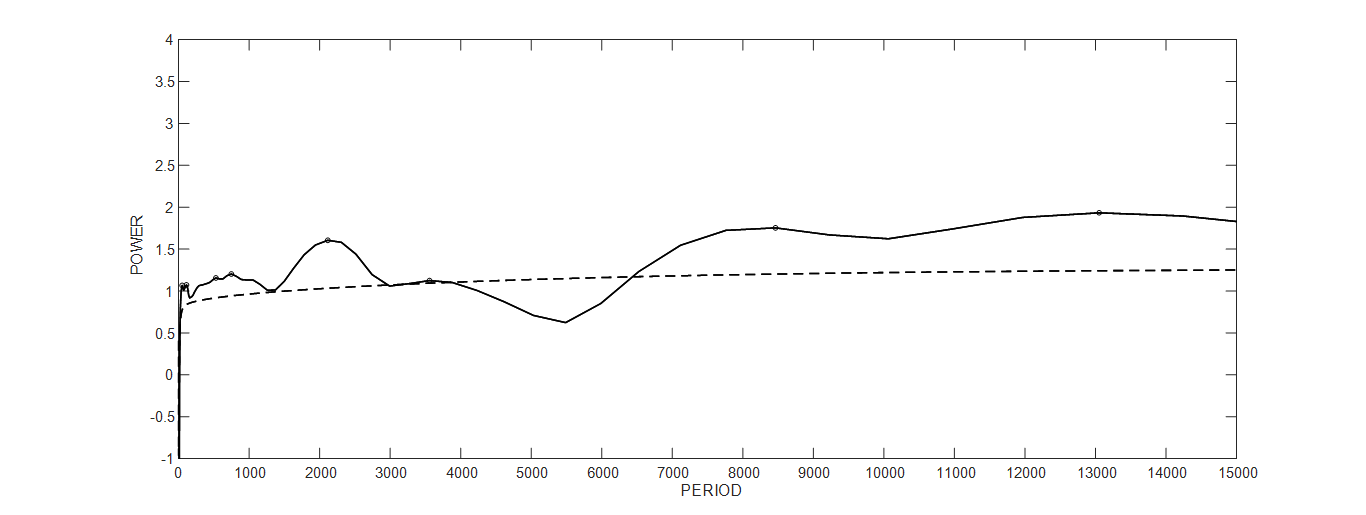


*Figure S3: Same as Figure 3 but for SARS-CoV-1*

*Table ST1: Same as Table 1 but for SARS-CoV-1*

**

*Table ST2: Same as Table 2 but for SARS-CoV-1*


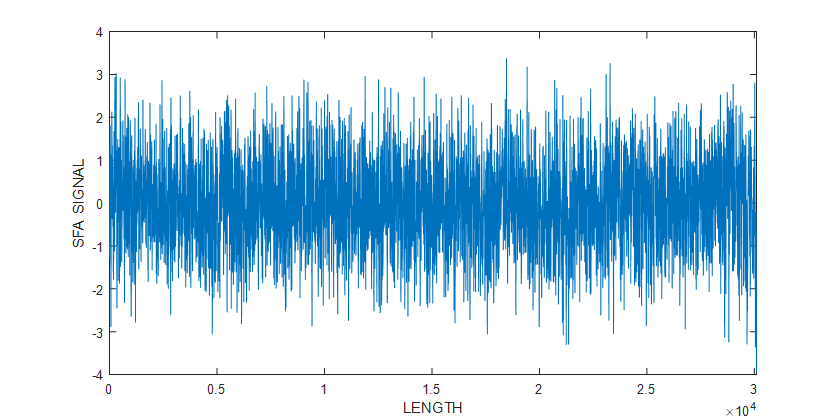


*Figure S4: Same as Figure 1 but for MERS*


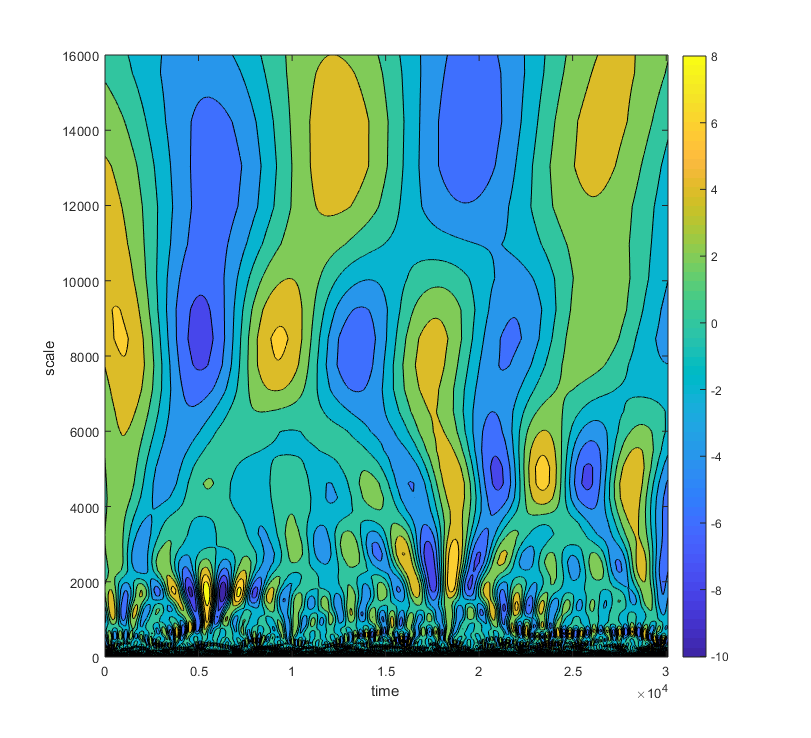


*Figure S5: Same as Figure 2 but for MERS*


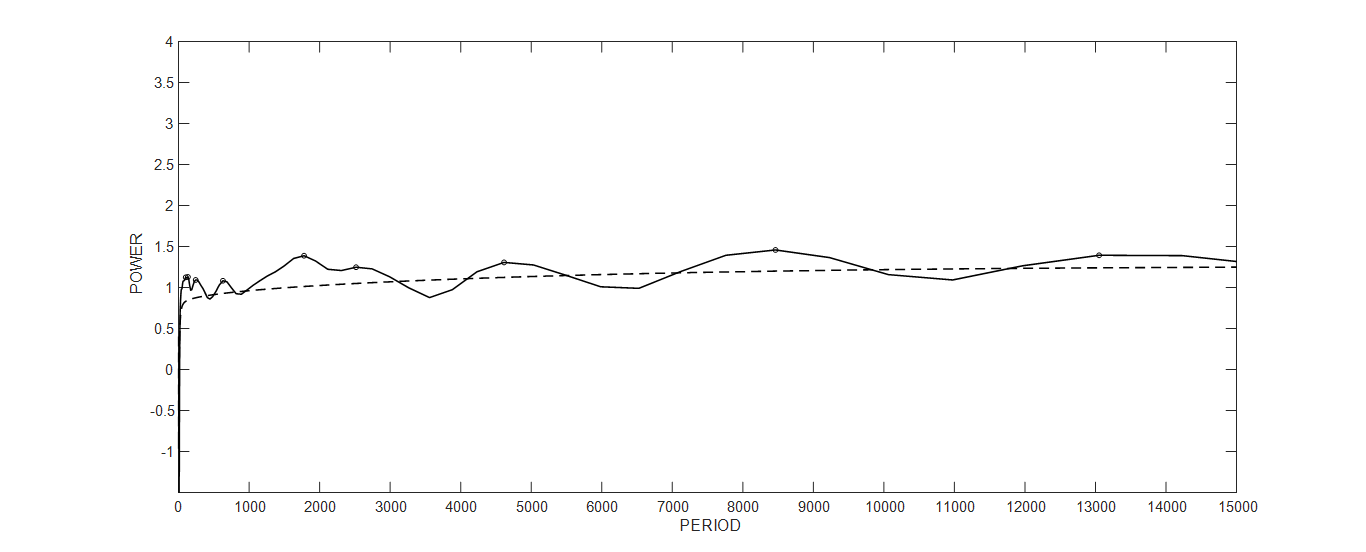


*Figure S6: Same as Figure 3 but for MERS*

**

*Table ST3: Same as Table 1 but for MERS*

**

*Table ST4:* *Same as Table 2 but for MERS*


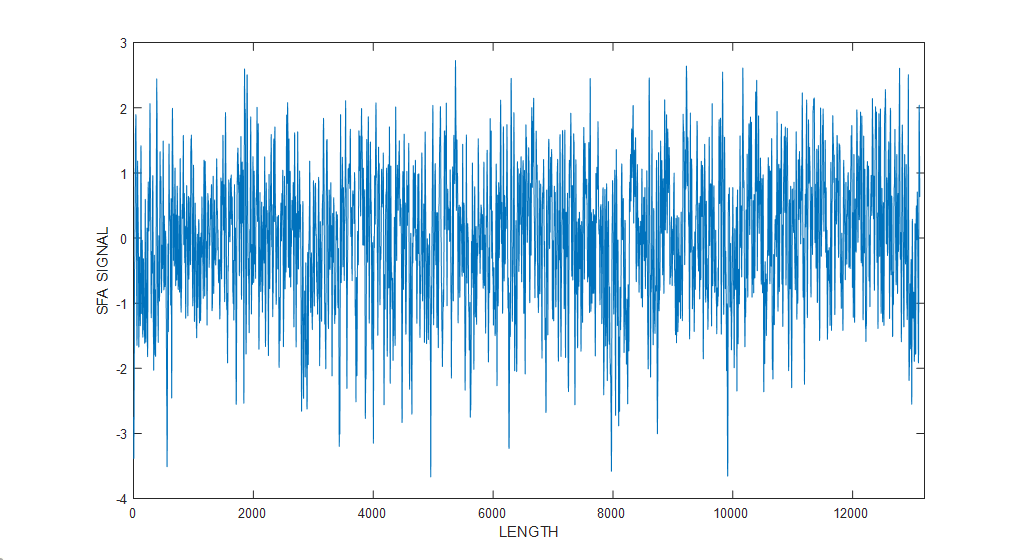


*Figure S7: Same as Figure 1 but for H1N1-1918*


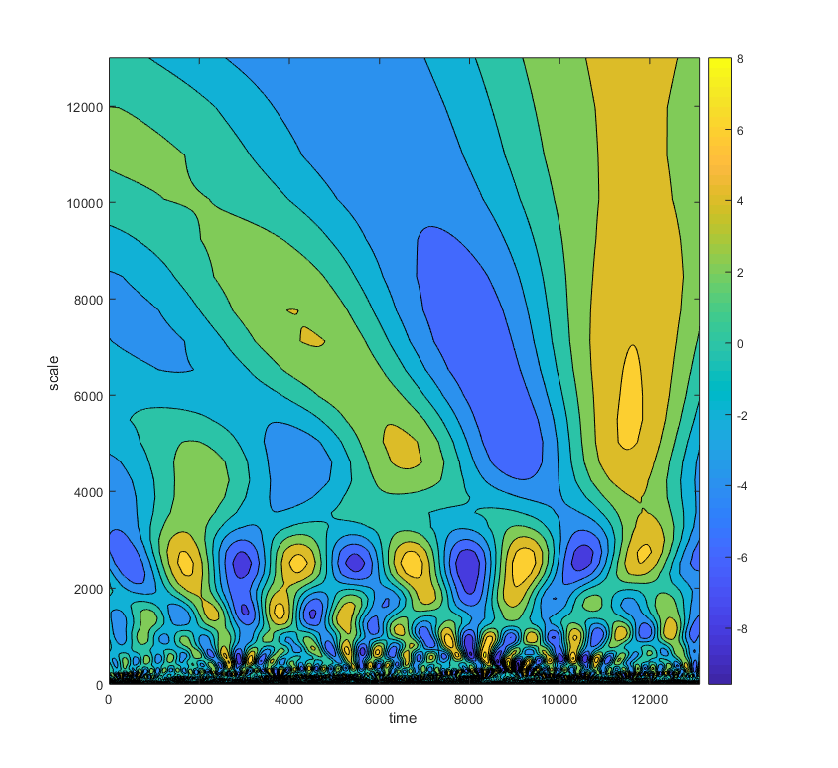


*Figure S8: Same as Figure 2 but for H1N1-1918*


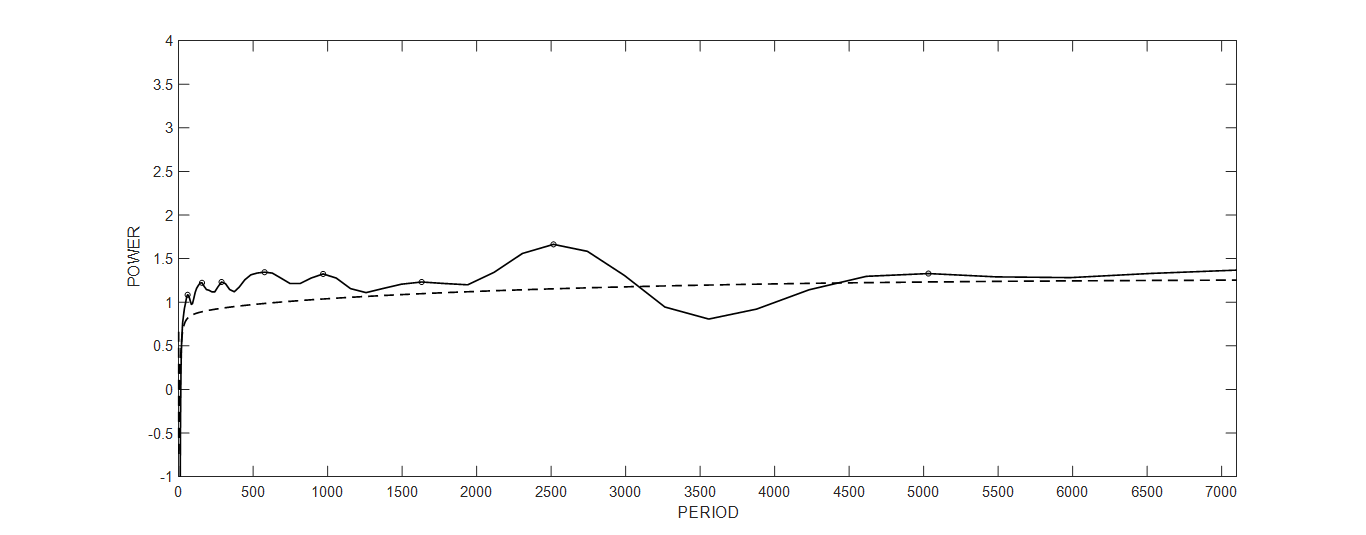


*Figure S9: Same as Figure 3 but for H1N1-1918*

*Table ST5: Same as Table 1 but for H1N1-1918*

| **Period** | **P1** | **P2** | **P3** | **P4** | **P5** | **P6** | **P7** | **P8** |
| --- | --- | --- | --- | --- | --- | --- | --- | --- |
| **P1** | 1 |  |  |  | 16 |  |  | 83 |
| **P2** |  | 1 |  |  |  |  | 16 | 32 |
| **P3** |  |  | 1 | 2 |  |  |  |  |
| **P4** |  |  |  | 1 |  |  |  |  |
| **P5** |  |  |  |  | 1 |  |  |  |
| **P6** |  |  |  |  |  | 1 |  |  |
| **P7** |  |  |  |  |  |  | 1 | 2 |
| **P8** |  |  |  |  |  |  |  | 1 |

*Table ST6: Same as Table 2 but for H1N1-1918*


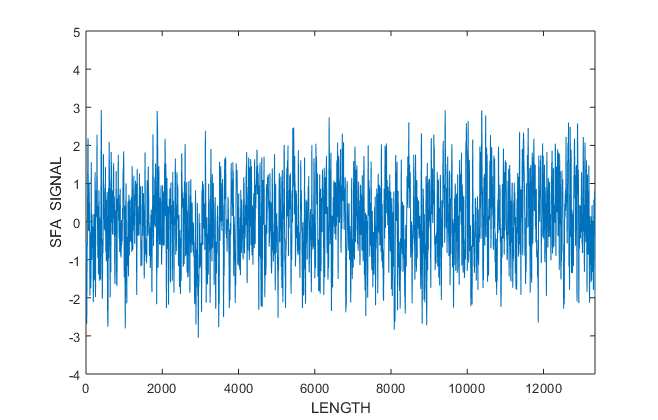


*Figure S10: Same as Figure 1 but for H1N1-2009*


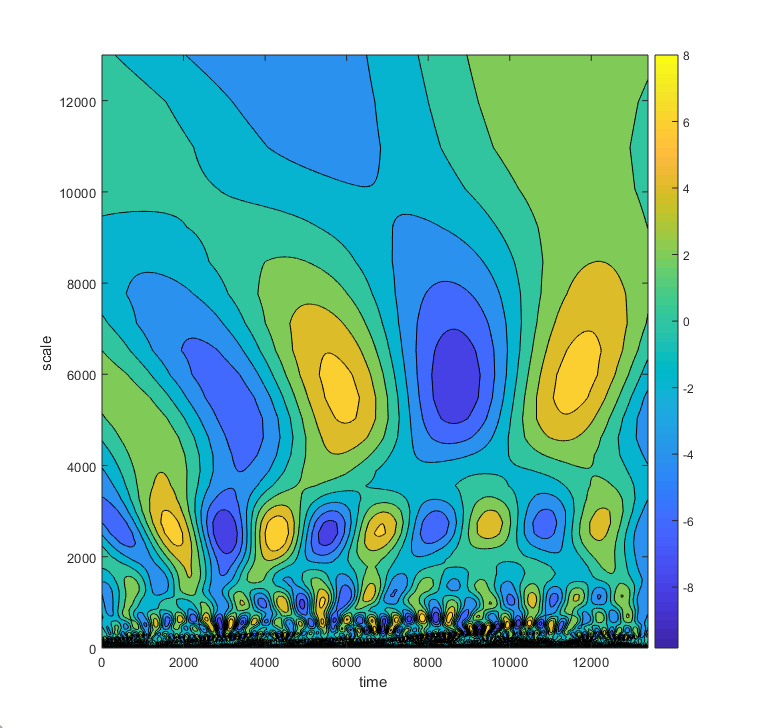


*Figure S11: Same as Figure 2 but for H1N1-2009*


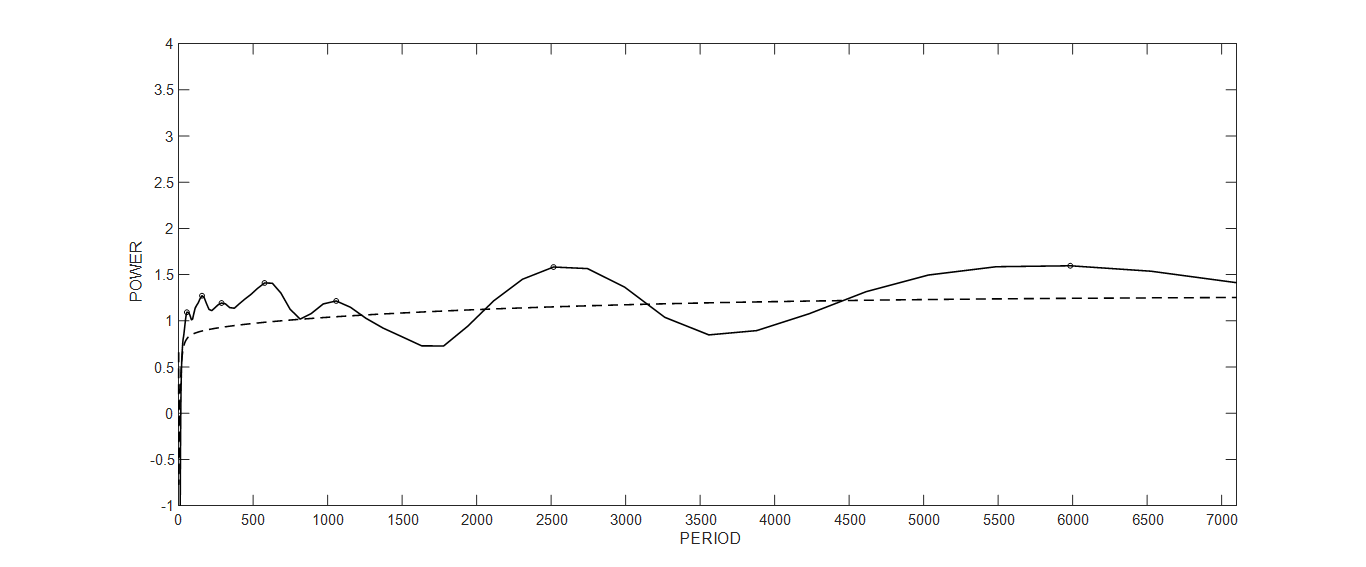


Figure S12: Same as Figure 3 but for H1N1-2009

*Table ST7: Same as Table 1 but for H1N1-2009*

| **Period** | **P1** | **P2** | **P3** | **P4** | **P5** | **P6** | **P7** |
| --- | --- | --- | --- | --- | --- | --- | --- |
| **P1** | 1 |  |  |  | 19 |  |  |
| **P2** |  | 1 |  |  |  | 16 | 38 |
| **P3** |  |  | 1 | 2 |  |  |  |
| **P4** |  |  |  | 1 |  |  |  |
| **P5** |  |  |  |  | 1 |  |  |
| **P6** |  |  |  |  |  | 1 |  |
| **P7** |  |  |  |  |  |  | 1 |

*Table ST8: Same as Table 2 but for H1N1-2009*

*
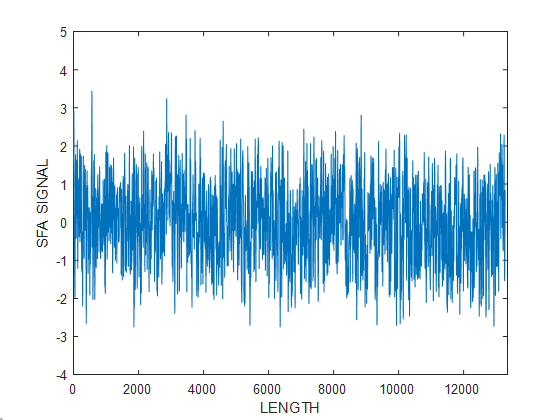
*

*Figure S13: Same as Figure 1 but for H2N2-1957*


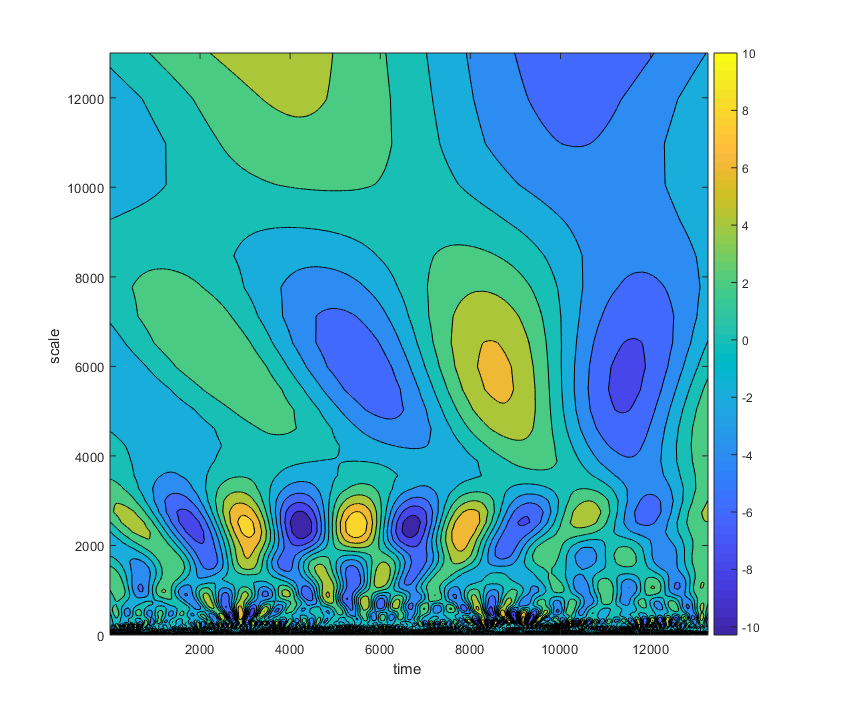


Figure S14: Same as Figure 2 but for H2N2-1957


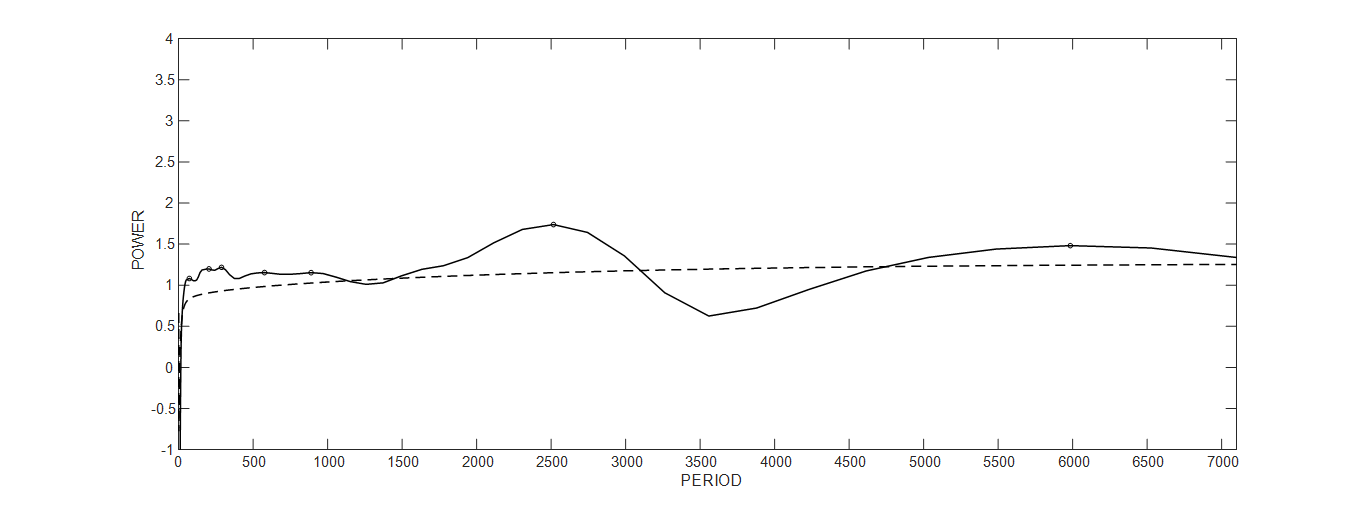


Figure S15: Same as Figure 3 but for H2N2-1957

*Table ST9: Same as Table 1 but for H2N2-1957*

| **Period** | **P1** | **P2** | **P3** | **P4** | **P5** | **P6** | **P7** |
| --- | --- | --- | --- | --- | --- | --- | --- |
| **P1** | 1 |  | 4 | 8 |  |  | 83 |
| **P2** |  | 1 |  |  |  |  |  |
| **P3** |  |  | 1 | 2 |  |  |  |
| **P4** |  |  |  | 1 |  |  |  |
| **P5** |  |  |  |  | 1 |  |  |
| **P6** |  |  |  |  |  | 1 |  |
| **P7** |  |  |  |  |  |  | 1 |

*Table ST10: Same as Table 2 but for H2N2-1957*


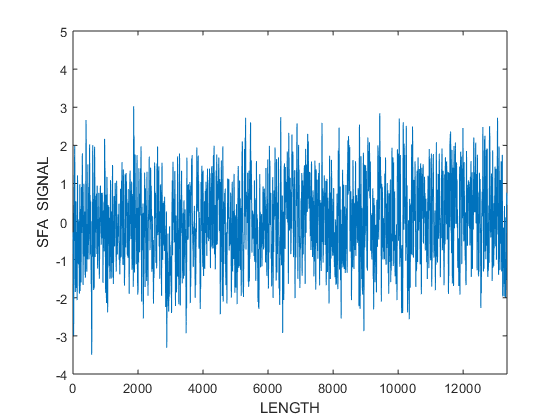


*Figure S16: Same as Figure 1 but for H3N2-1968*


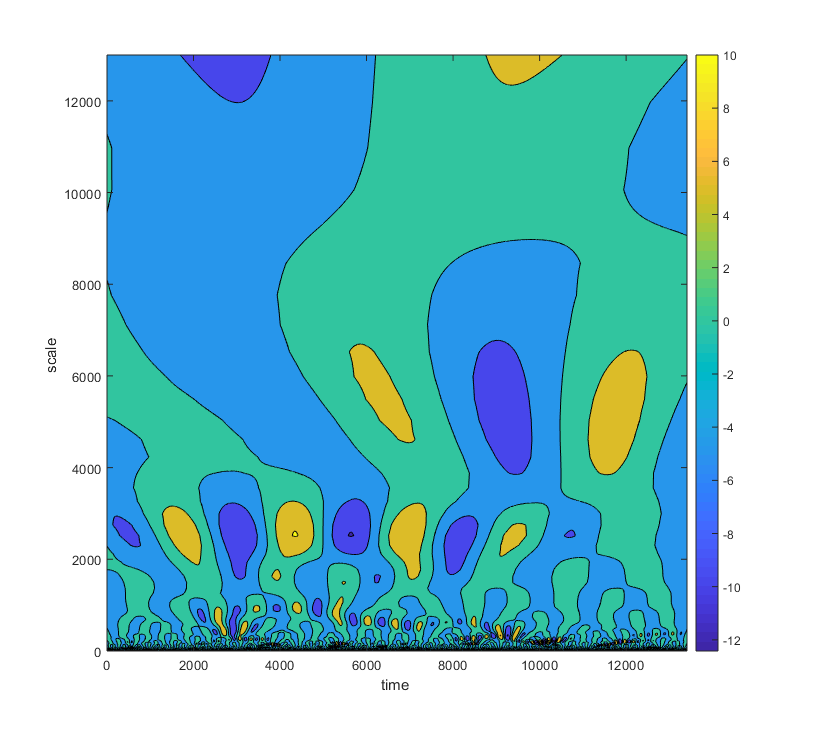


*Figure S17: Same as Figure 2 but for H3N2-1968*


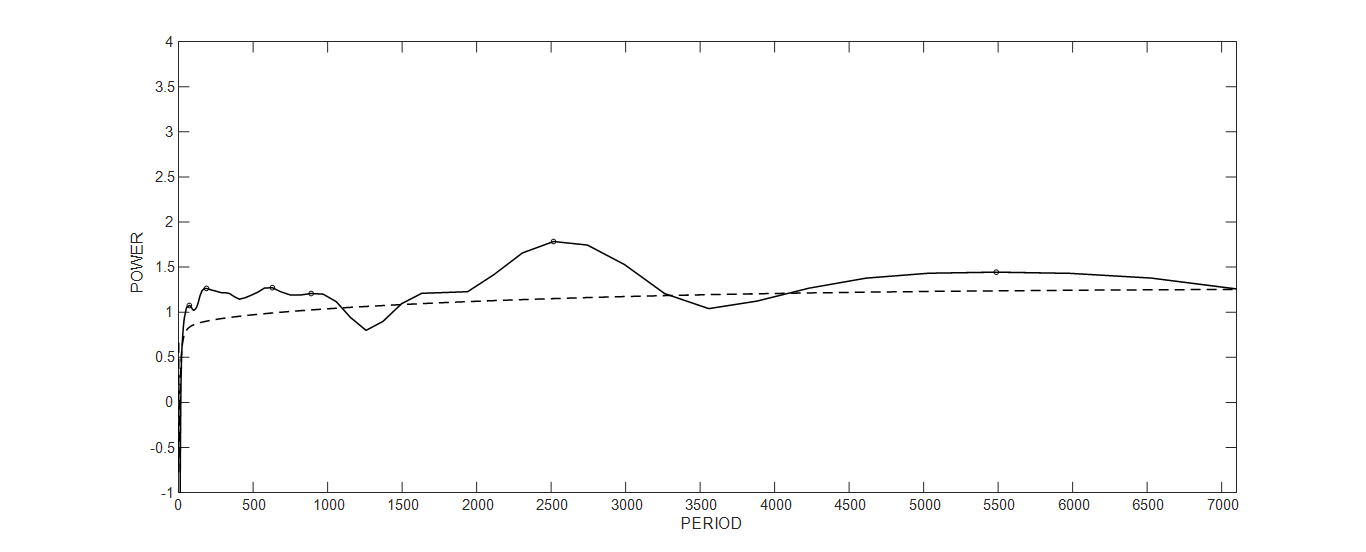


*Figure S18: Same as Figure 3 but for H3N2-1968*

*Table ST11: Same as Table 1 but for H3N2-1968*

*Table ST12: Same as Table 2 but for H3N2-1968*
