# Supplementary material for: An application of slow feature analysis to the genetic sequences of coronaviruses and influenza viruses
Source: Hum Genomics. 2021 May 7;15:26. doi: 10.1186/s40246-021-00327-2 (PMC8103670; doi:10.1186/s40246-021-00327-2)
Supplement: Supplementary file 2 — Additional file 2. DNA sequences used in the study [file 40246_2021_327_MOESM2_ESM.docx]

**Supplementary Material 2**

**DNA sequences used in the study**

**An Application of Slow Feature Analysis to the Genetic Sequences of**

**Coronaviruses and Influenza viruses**

Anastasios A. Tsonis^1,2,^* Geli Wang^3^, Lvyi Zhang^3^, Wenxu Lu^3^, Aristotle Kayafas^4^, and Katia Del Rio-Tsonis^4^*

1 Department of Mathematical Sciences, Atmospheric Sciences Group, University of Wisconsin-Milwaukee, Milwaukee, WI 53201, USA

2 Hydrologic Research Center, San Diego, CA 92127, USA

^3^ Key Laboratory of Middle Atmosphere and Global Environment Observation (LAGEO), Institute of Atmospheric Physics, Chinese Academy of Sciences, Beijing 100029, China

^4^Department of Biology and Center for Visual Sciences at Miami University, Oxford, OH 45056, USA

* Corresponding authors

**>Influenza A virus (A/Brevig Mission/1/1918(H1N1))**

**ATGGAAAGAATAAAAGAACTAAGGGATCTAATGTCGCAGTCTCGCACTCGCGAGATACTCACAAAAACCA**

**CCGTGGACCATATGGCCATAATCAAGAAGTACACATCAGGAAGACAGGAGAAGAACCCCGCACTTAGGAT**

**GAAATGGATGATGGCAATGAAATATCCAATTACAGCAGACAAGAGGATAATGGAAATGATTCCTGAGAGA**

**AATGAGCAAGGACAAACTCTATGGAGTAAAACGAACGATGCCGGATCAGACCGAGTGATGGTATCACCTC**

**TGGCTGTGACATGGTGGAATAGGAATGGACCAACGACAAGTGCAGTTCACTATCCAAAAATCTACAAAAC**

**TTATTTTGAAAAAGTCGAAAGGTTAAAACATGGAACCTTTGGCCCTGTCCATTTCAGAAACCAAGTCAAA**

**ATACGTCGAAGAGTTGACATAAATCCTGGTCATGCAGATCTCAGTGCCAAAGAGGCACAGGATGTAATCA**

**TGGAAGTTGTTTTCCCAAACGAAGTGGGAGCCAGGATACTAACATCGGAATCGCAACTGACAATAACCAA**

**AGAAAAGAAAGAAGAACTCCAAGATTGTAAAATTTCTCCTTTAATGGTGGCATACATGTTGGAGAGAGAA**

**CTGGTCCGAAAAACAAGATTCCTCCCAGTGGCTGGTGGGACAAGCAGTGTGTATATTGAAGTGTTGCATT**

**TGACTCAAGGAACATGCTGGGAACAGATGTACACTCCAGGAGGGGAAGTGAGGAATGATGATGTTGATCA**

**AAGCTTAATTATTGCTGCTAGGAACATAGTGAGAAGAGCGACAGTGTCAGCAGATCCACTAGCATCTCTG**

**TTGGAAATGTGCCACAGCACACAGATTGGTGGAATAAGGATGGTAGACATCCTTAGGCAGAACCCGACAG**

**AAGAGCAAGCCGTGGATATATGCAAGGCAGCAATGGGCCTGAGAATTAGCTCATCCTTTAGCTTTGGCGG**

**ATTCACATTTAAGAGGACAAGTGGCTCATCAGTCAAGAGGGAGGAAGAAGTGCTTACAGGCAATCTTCAA**

**ACATTGAAGATAAGAGTGCATGAGGGATATGAAGAGTTCACAATGGTTGGGAGAAGAGCAACAGCTATAC**

**TCAGAAAAGCGACCAGGAGATTGATTCAGCTGATAGTGAGTGGGAGAGACGAACAGTCGATTGCCGAGGC**

**AATAATTGTGGCCATGGTATTTTCACAAGAGGATTGTATGATAAAGGCAGTTAGGGGTGATCTGAATTTC**

**GTTAATAGGGCGAATCAGCGATTGAATCCTATGCATCAACTTTTGAGGCATTTTCAAAAGGATGCGAAAG**

**TGCTTTTTCAAAATTGGGGAATTGAACCCATCGACAATGTGATGGGAATGATTGGGATACTGCCCGACAT**

**GACTCCAAGTACTGAGATGTCAATGAGAGGAGTGAGAGTCAGCAAAATGGGAGTAGATGAGTACTCCAGC**

**ACAGAGAGGGTGGTGGTGAGCATTGACCGCTTTTTAAGAGTCCGGGACCAACGAGGAAACGTACTACTGT**

**CTCCTGAGGAGGTCAGCGAAACACAGGGAACAGAGAAATTGACGATAACTTATTCATCGTCAATGATGTG**

**GGAGGTTAATGGCCCTGAATCAGTGTTGGTCAACACCTATCAGTGGATCATCAGAAACTGGGAAACTGTT**

**AAAATTCAGTGGTCACAGAATCCTACAATGCTATACAATAAAATGGAATTTGAGCCATTTCAGTCTTTAG**

**TTCCTAAGGCCGCTAGAGGTCAATACAGTGGGTTTGTGAGAACTCTGTTCCAGCAAATGAGGGATGTGCT**

**TGGGACATTTGACACCGTTCAGATAATAAAACTTCTTCCCTTTGCAGCCGCTCCACCAAAGCAAAGTAGA**

**ATGCAGTTCTCCTCTCTGACTGTGAATGTGAGAGGATCAGGAATGAGAATACTTGTAAGGGGCAATTCTC**

**CCGTATTCAACTACAACAAGGCCACTAAGAGACTCACAGTTCTCGGAAAGGATGCAGGTGCTTTAACTGA**

**AGACCCAGATGAAGGCACAGCTGGAGTGGAGTCTGCTGTTCTGAGAGGATTCCTCATTCTGGGCAAAGAA**

**GACAGGAGATATGGGCCAGCATTAAGCATCAATGAACTGAGCAATCTTGCGAAAGGGGAGAAGGCTAATG**

**TGCTAATTGGGCAAGGAGACGTGGTGTTGGTAATGAAACGGAAACGGGACTCTAGCATACTTACTGACAG**

**CCAGACAGCGACCAAAAGAATTCGGATGGCCATCAATTAGATGGATGTCAATCCGACTTTACTTTTCTTG**

**AAAGTGCCAGCGCAAAATGCTATAAGCACAACGTTCCCTTATACTGGAGACCCTCCTTACAGCCATGGGA**

**CAGGAACAGGATACACCATGGATACTGTCAACAGGACACATCAGTACTCAGAAAAGGGAAGATGGACAAC**

**AAACACCGAGACTGGAGCACCACAACTCAACCCGATTGATGGACCACTGCCAGAAGACAATGAACCAAGT**

**GGTTATGCCCAAACAGATTGTGTATTGGAAGCAATGGCTTTCCTTGAGGAGTCCCACCCCGGTATCTTTG**

**AAAACTCGTGTCTTGAAACGATGGAGGTTGTTCAGCAAACACGAGTGGACAAGCTGACGCAAGGCCGACA**

**GACCTATGACTGGACTCTAAATAGGAACCAGCCTGCTGCAACAGCATTGGCCAACACAATAGAAGTGTTC**

**AGATCAAATGGCCTCACGGCCAATGAATCTGGAAGGCTCATAGACTTCCTCAAGGATGTCATGGAGTCAA**

**TGGACAAAGAAGAAATGGAAATCACAACTCATTTTCAGAG**

**AAAGAGAAGAGTGAGAGACAACATGACTAAGAAAATGGTGACACAGAGAACAATAGGTAAAAAGAAGCAG**

**AGGTTGAACAAAAGGAGTTATCTAATCAGGGCACTGACCCTGAACACAATGACCAAAGATGCTGAGAGAG**

**GGAAGCTAAAACGGAGAGCAATTGCAACCCCAGGGATGCAAATACGAGGATTTGTATACTTTGTTGAGAC**

**ACTGGCAAGGAGTATATGTGAGAAACTTGAACAATCAGGATTGCCAGTTGGAGGTAATGAGAAGAAAGCC**

**AAGTTGGCAAATGTTGTAAGGAAGATGATGACCAATTCTCAGGACACTGAGCTTTCTTTCACCATCACTG**

**GAGATAACACCAAATGGAACGAAAATCAGAATCCTCGGATGTTTTTGGCCATGATCACATACATAACCAG**

**AAATCAGCCCGAATGGTTCAGAAATGTTCTAAGTATTGCTCCAATAATGTTCTCAAACAAAATGGCGAGA**

**CTGGGGAAGGGGTACATGTTCGAGAGCAAGAGTATGAAACTTAGAACTCAAATACCTGCAGAGATGCTAG**

**CAAGCATCGATTTGAAATATTTCAATGATTCAACAAGAAAGAAAATTGAAAAAATCCGACCGCTCTTAAT**

**AGATGGGACTGCATCATTGAGCCCTGGAATGATGATGGGCATGTTCAATATGTTAAGCACTGTATTAGGC**

**GTCTCCATCCTGAATCTTGGACAAAAGAGATACACCAAGACTACTTACTGGTGGGATGGTCTTCAATCTT**

**CTGATGATTTTGCTCTGATTGTGAATGCACCCAATCATGAGGGAATTCAAGCCGGAGTCGACAGGTTTTA**

**TCGAACCTGTAAGCTGCTTGGAATCAATATGAGCAAGAAAAAGTCTTACATAAACAGAACAGGCACATTT**

**GAATTCACAAGTTTTTTCTATCGCTATGGGTTTGTTGCCAATTTCAGCATGGAGCTTCCCAGCTTTGGGG**

**TGTCTGGGATCAACGAGTCTGCGGACATGAGTATTGGAGTTACTGTCATCAAAAACAATATGATAAACAA**

**TGATCTTGGTCCAGCAACAGCTCAAATGGCCCTTCAGTTGTTCATCAAAGATTACAGGTACACGTACCGG**

**TGCCACAGGGGTGACACCCAAATACAAACCCGAAGATCATTTGAAATAAAGAAATTGTGGGAGCAAACCC**

**GTTCCAAAGCTGGACTGCTGGTCTCCGACGGAGGCCCCAATTTGTACAACATTAGAAATCTCCACATTCC**

**TGAAGTCTGCTTGAAATGGGAATTGATGGATGAGGATTACCAGGGGCGTTTATGCAACCCACTGAACCCA**

**TTTGTCAGCCATAAAGAAATTGAATCAGTGAATAATGCAGTGATGATGCCAGCACATGGTCCAGCCAAAA**

**ACATGGAGTATGATGCTGTTGCAACAACACACTCCTGGATTCCCAAAAGAAATCGATCCATCTTGAACAC**

**AAGCCAGAGAGGAATACTTGAAGATGAACAAATGTACCAAAAATGCTGCAATTTATTTGAAAAATTCTTC**

**CCCAGCAGTTCATACAGGAGACCAGTCGGGATTTCCAGTATGGTGGAGGCCATGGTTTCTAGGGCCCGAA**

**TTGATGCACGGATTGATTTCGAATCTGGAAGGATAAAGAAAGAGGAGTTCGCTGAGATCATGAAGATCTG**

**TTCCACCATTGAAGAGCTCAGACGGCAAAAGTAGATGGAAGACTTTGTGCGACAATGCTTCAATCCGATG**

**ATTGTCGAGCTTGCGGAAAAAGCAATGAAAGAGT**

**ATGGAGAGGACCTGAAAATCGAAACAAACAAATTTGCAGCAATATGCACTCACTTGGAAGTATGCTTCAT**

**GTATTCAGATTTTCACTTCATCAATGAGCGAGGCGAATCAATAATCGTAGAATCTGGCGATCCAAATGCA**

**CTCTTGAAGCACAGATTTGAAATAATCGAGGGAAGAGATCGCACAATGGCCTGGACGGTGGTAAACAGTA**

**TTTGCAACACTACAGGGGCTGAGAAACCAAAGTTTCTCCCAGATCTGTATGATTACAAGGAGAATAGATT**

**CATTGAGATTGGAGTAACAAGGAGAGAAGTCCACATATACTATCTGGAAAAGGCCAATAAAATAAAATCT**

**GAGAAGACACACATCCACATTTTCTCGTTCACTGGGGAAGAAATGGCCACAAAGGCCGACTACACTCTCG**

**ATGAGGAGAGCAGGGCTAGGATCAAAACCAGACTATTCACCATAAGACAAGAGATGGCCAGCAGAGGCCT**

**CTGGGATTCCTTTCGTCAGTCCGAGAGAGGCGAAGAGACAATTGAAGAAAGATTTGAAATCACAGGAACA**

**ATGCGCAGGCTTGCCGACCAAAGTCTCCCACCGAACTTCTCCAGCCTTGAAAACTTTAGAGCCTATGTGG**

**ATGGATTCGAACCGAACGGCTACATTGAGGGCAAGCTTTCTCAAATGTCCAAAGAAGTAAATGCTAGAAT**

**TGAACCTTTTTTGAAGACAACACCACGCCCACTTAGGCTGCCAGATGGGCCTCCCTGTTCTCAGCGGTCC**

**AAATTCCTGCTGATGGATGCCTTGAAATTAAGCATTGAGGACCCAAGCCATGAAGGAGAGGGGATACCGC**

**TATATGATGCGATCAAGTGCATGAGAACATTCTTTGGGTGGAAGGAACCCAATGTTGTTAAGCCACACGA**

**AAAGGGAATAAATCCAAATTATCTCTTGGCATGGAAGCAAGTACTGGCAGAACTGCAGGATATTGAGAAT**

**GAGGAGAAAATTCCAAAGACTAAAAATATGAAGAAAACGAGCCAGTTAAAGTGGGCACTTGGTGAGAATA**

**TGGCACCAGAAAAAGTGGATTTTGACGACTGTAAAGATGTAAGCGATCTGAAGCAATATGATAGTGATGA**

**ACCGGAATTGAGGTCGCTTGCAAGTTGGATTCAGAGTGAGTTCAACAAGGCATGCGAACTGACCGATTCA**

**AGCTGGATAGAGCTCGATGAGATTGGAGAAGATGTGGCTCCAATTGAACACATTGCAAGCATGAGAAGGA**

**ATTATTTCACAGCAGAGGTGTCTCATTGCAGAGCCACAGAATACATAATGAAGGGGGTATACATCAATAC**

**TGCCTTGCTTAATGCATCCTGTGCAGCAATGGATGATTTCCAATTAATTCCAATGATAAGCAAGTGTAGA**

**ACTAAGGAGGGAAGACGAAAGACCAATTTGTACGGGTTCATCATAAAAGGAAGATCCCACTTAAGGAATG**

**ACACCGACGTGGTAAATTTTGTGAGCATGGAGTTTTCCCTCACTGATCCAAGACTTGAACCGCACAAATG**

**GGAGAAGTACTGTGTCCTTGAGATAGGGGATATGCTTCTAAGAAGTGCCATAGGCCAAGTGTCAAGGCCC**

**ATGTTCTTGTATGTGAGGACAAATGGGACATCAAAAATTAAAATGAAATGGGGAATGGAAATGAGACGTT**

**GCCTCCTTCAGTCACTTCAACAAATCGAGAGTATGATTGAAGCTGAGTCCTCTGTCAAGGAGAAAGACAT**

**GACCAAAGAATTCTTTGAGAACAAATCAGAAACATGGCCCATTGGAGAGTCCCCCAAAGGAGTGGAGGAA**

**GGTTCCATTGGGAAGGTCTGCAGGACTTTGTTGGCAAAATCGGTATTCAACAGCTTGTATGCATCTCCAC**

**AACTAGAAGGATTCTCAGCTGAATCAAGAAAACTGCTTCTTATCGTTCAGGCTCTTAGGGACAACCTGGA**

**ACCTGGAACCTTTGATCTTGGGGGGCTATATGAAGCAATTGAGGAGTGCCTGATTAATGATCCCTGGGTT**

**TTGCTTAATGCGTCTTGGTTCAACTCCTTCCTCACACATGCACTGAGATAGATGGAGGCAAGACTACTGG**

**TCTTGTTATGTGCATTTGCAGCTACAAATGCAGACACAATATGTATAGGCT**

**ACCATGCGAATAACTCAACCGACACTGTTGACACAGTACTCGAAAAGAATGTGACCGTGACACACTCTGT**

**TAACCTGCTCGAAGACAGCCACAACGGAAAACTATGTAAATTAAAAGGAATAGCCCCATTACAATTGGGG**

**AAATGTAATATCGCCGGATGGCTCTTGGGAAACCCGGAATGCGATTTACTGCTCACAGCGAGCTCATGGT**

**CCTATATTGTAGAAACATCGAACTCAGAGAATGGAACATGTTACCCAGGAGATTTCATCGACTATGAAGA**

**ACTGAGGGAGCAATTGAGCTCAGTGTCATCGTTTGAAAAATTCGAAATATTTCCCAAGACAAGCTCGTGG**

**CCCAATCATGAAACAACCAAAGGTGTAACGGCAGCATGCTCCTATGCGGGAGCAAGCAGTTTTTACAGAA**

**ATTTGCTGTGGCTGACAAAGAAGGGAAGCTCATACCCAAAGCTTAGCAAGTCCTATGTGAACAATAAAGG**

**GAAAGAAGTCCTTGTACTATGGGGTGTTCATCATCCGCCTACCGGTACTGATCAACAGAGTCTCTATCAG**

**AATGCAGATGCTTATGTCTCTGTAGGGTCATCAAAATATAACAGGAGATTCACCCCGGAAATAGCAGCGA**

**GACCCAAAGTAAGAGATCAAGCTGGGAGGATGAACTATTACTGGACATTACTAGAACCCGGAGACACAAT**

**AACATTTGAGGCAACTGGAAATCTAATAGCACCATGGTATGCTTTCGCACTGAATAGAGGTTCTGGATCC**

**GGTATCATCACTTCAGACGCACCAGTGCATGATTGTAACACGAAGTGTCAAACACCCCATGGTGCTATAA**

**ACAGCAGTCTCCCTTTCCAGAATATACATCCAGTCACAATAGGAGAGTGCCCAAAATACGTCAGGAGTAC**

**CAAATTGAGGATGGCTACAGGACTAAGAAACATTCCATCTATTCAATCCAGGGGTCTATTTGGAGCCATT**

**GCCGGTTTTATTGAGGGGGGATGGACTGGAATGATAGATGGATGGTATGGTTATCATCATCAGAATGAAC**

**AGGGATCAGGCTATGCAGCGGATCAAAAAAGCACACAAAATGCCATTGACGGGATTACAAACAAGGTGAA**

**TTCTGTTATCGAGAAAATGAACACCCAATTCACAGCAGTGGGTAAAGAATTCAACAACTTAGAAAGAAGG**

**ATAGAAAATTTAAATAAAAAAGTCGATGATGGATTTCTGGATATTTGGACATATAATGCAGAATTGTTAG**

**TTCTACTGGAAAATGAAAGAACCCTGGATTTCCATGACTCAAATGTAAGGAATCTGTATGAGAAAGTAAA**

**AAGCCAATTAAAGAATAATGCCAAGGAAATCGGAAATGGATGTTTTGAGTTCTACCACAAGTGTGACGAT**

**GCATGCATGGAAAGTGTAAGAAATGGGACTTATGATTACCCAAAATATTCAGAAGAATCAAAGTTGAACA**

**GAGAAGAAATAGATGGAGTGAAATTAGAATCAATGGGGGTCTATCAGATTCTGGCGATCTACTCAACTGT**

**CGCCAGTTCACTAGTGCTGTTAGTCTCCCTGGGGGCAATCAGCTTCTGGATGTGTTCTAATGGGTCTTTG**

**CAGTGCAGAATATGCATTTGAATGGCGTCTCAAGGCACCAAACGATCTTACGAACAGATGGAGACTGATG**

**GGGAACGCCAGAATGCCACTG**

**AAATCAGAGCATCTGTCGGAAGAATGATTGGTGGAATTGGACGATTCTACATCCAAATGTGCACCGAACT**

**TAAACTCAGTGATTATGAGGGACGGCTGATCCAGAACAGCATAACAATAGAGAGAATGGTGCTCTCTGCT**

**TTTGACGAGAGGAGGAATAAATATCTGGAAGAACATCCCAGCGCTGGGAAAGACCCTAAGAAAACTGGAG**

**GACCCATATACAGGAGAATAGATGGAAAGTGGATGAGAGAACTCATCCTTTATGACAAAGAAGAAATAAG**

**GAGAATCTGGCGCCAAGCTAATAATGGTGAAGATGCAACAGCTGGTCTGACTCACATGATGATCTGGCAT**

**TCCAATTTGAATGATGCCACTTATCAGAGGACAAGAGCTCTTGTTCGCACCGGAATGGATCCCAGGATGT**

**GCTCTCTGATGCAAGGTTCAACTCTCCCTAGGAGGTCTGGAGCCGCAGGTGCTGCAGTCAAAGGAGTTGG**

**AACAATGGTGATGGAGTTGATCAGGATGATCAAACGTGGGATCAATGATCGGAACTTCTGGAGGGGTGAG**

**AATGGACGAAGGACAAGAATTGCCTATGAAAGAATGTGCAACATTCTCAAAGGGAAATTTCAAACAGCTG**

**CACAGAGAGCAATGATGGATCAAGTGAGAGAGAGCCGGAATCCAGGAAATGCTGAAATTGAAGATCTCAT**

**CTTTCTGGCACGGTCTGCACTCATATTGAGAGGGTCAGTTGCTCACAAGTCCTGCCTGCCTGCCTGTGTG**

**TATGGACCTGCTGTAGCCAGTGGATACGACTTTGAAAGAGAGGGATACTCTCTGGTCGGAATAGACCCTT**

**TCAGACTGCTTCAAAACAGCCAAGTATACAGTCTAATCAGACCAAATGAGAATCCAGCACACAAGAGTCA**

**ACTGGTATGGATGGCATGCCATTCTGCTGCATTTGAAGATCTGAGAGTATCAAGCTTCATCAGAGGGACA**

**AGAGTGGTTCCAAGAGGGAAGCTTTCCACTAGAGGGGTTCAGATTGCTTCCAATGAAAACATGGAGACTA**

**TGGACTCCAGTACCCTTGAACTGAGAAGCAGATACTGGGCCATAAGGACCAGAAGTGGAGGAAACACTAA**

**TCAACAGAGGGCATCTGCGGGCCAAATCAGCGTGCAACCTACATTCTCAGTACAGAGAAACCTCCCTTTT**

**GAGAGAGCAACCATTATGGCAGCATTCACTGGGAACACAGAGGGGAGAACATCTGACATGAGAACCGAAA**

**TCATAAGGATGATGGAAAGTGCAAGACCAGAAGATGTGTCTTTCCAGGGGCGGGGAGTCTTCGAGCTCTC**

**GGACGAAAAGGCAACGAGCCCGATCGTGCCTTCCTTTGACATGAGTAATGAAGGATCTTATTTCTTCGGA**

**GACAATGCAGAGGAGTACGACAATTAAATGAATCCAAATCAAAAAATAATAACCATTGGGTCAATCTGTA**

**TGGTAGTCGGAATAATTAGCCTAATAT**

**TGCAAATAGGGAATATAATCTCAATATGGGTTAGCCATTCAATTCAAACTGGAAATCAAAACCATCCTGA**

**AACATGCAACCAAAGCATCATTACCTATGAAAATAACACCTGGGTGAATCAAACATATGTTAACATTAGC**

**AATACTAACGTTGTTGCTGGACAGGATGCAACTTCAGTGATATTAACCGGCAATTCCTCTCTTTGTCCCA**

**TCAGTGGGTGGGCTATATACAGCAAAGACAATGGCATAAGAATTGGTTCCAAAGGAGACGTTTTTGTCAT**

**AAGAGAGCCATTTATTTCATGCTCTCACTTGGAATGCAGGACCTTTTTTCTGACTCAAGGCGCCTTGCTG**

**AATGACAAGCATTCAAATGGGACCGTCAAGGACAGAAGCCCCTATAGAACCTTAATGAGCTGCCCTGTTG**

**GTGAAGCTCCGTCTCCGTACAATTCAAGGTTCGAATCGGTTGCTTGGTCAGCAAGTGCATGCCATGATGG**

**CATGGGCTGGCTAACAATCGGAATTTCAGGTCCAGATAATGGAGCAGTGGCTGTATTAAAATACAACGGT**

**ATAATAACAGACACCATCAAAAGTTGGAGGAACAACATATTGAGAACGCAAGAGTCTGAATGTGCCTGTG**

**TAAATGGTTCATGTTTTACTATAATGACCGATGGCCCAAGTAATGGGCAGGCCTCGTACAAAATTTTAAA**

**GATAGAGAAGGGGAAGGTTACTAAATCAATTGAGTTGAATGCACCTAATTACCACTACGAGGAATGTTCC**

**TGTTACCCTGATACAGGTAAAGTGATGTGTGTGTGCAGAGACAATTGGCATGGTTCGAATCGACCATGGG**

**TGTCTTTCGATCAAAACCTGGATTATCAAATAGGGTACATCTGCAGTGGGGTTTTCGGTGACAATCCGCG**

**TCCCAATGATGGAACAGGCAGCTGTGGTCCAGTGTCTTCTAATGGAGCAAATGGAATAAAGGGATTTTCA**

**TTTAGGTATGATAATGGTGTTTGGATAGGAAGAACTAAAAGTACCAGTTCCAGAAGCGGGTTTGAGATGA**

**TTTGGGATCCTAATGGATGGACAGAGACTGATAGTAGTTTCTCTGTGAGACAAGATATTGTAGCAATAAC**

**TGATTGGTCGGGGTACAGCGGGAGTTTCGTTCAACATCCTGAGCTAACAGGGCTGGACTGCATGAGGCCT**

**TGCTTCTGGGTTGAATTAATCAGGGGACAACCTAAAGAGAATACAATCTGGACTAGTGGGAGCAGCATTT**

**CCTTTTGTGGCGTAAATAGTGATACTGTAGGTTGGTCTTGGCCAGACGGTGCTGAGTTGCCATTCAGCAT**

**TGACAAGTAGATGAGTCTTTTAACCGAGGTCGAAACGTACGTTCTCTCTATCGTCCCGTCAGGCCCCCTC**

**AAAGCCGAGA**

**TCGCGCAGAGACTTGAAGATGTCTTTGCAGGGAAGAACACCGATCTTGAGGCTCTCATGGAATGGCTAAA**

**GACAAGACCAATCCTGTCACCTCTGACTAAGGGGATTTTAGGATTTGTGTTCACGCTCACCGTGCCCAGT**

**GAGCGAGGACTGCAGCGTAGACGCTTTGTCCAAAATGCCCTTAATGGGAACGGGGATCCAAATAACATGG**

**ACAGAGCAGTTAAACTGTACAGGAAGCTTAAGAGGGAGATAACATTCCATGGGGCCAAAGAAGTAGCACT**

**CAGTTATTCCGCTGGTGCACTTGCCAGTTGTATGGGCCTCATATACAACAGGATGGGGACTGTGACCACT**

**GAAGTGGCATTTGGCCTGGTATGCGCAACCTGTGAACAGATTGCTGATTCCCAGCATCGGTCTCACAGGC**

**AAATGGTGACAACAACCAATCCACTAATCAGACATGAGAACAGAATGGTACTGGCCAGCACTACGGCTAA**

**GGCTATGGAGCAAATGGCTGGATCGAGTGAGCAAGCAGCAGAGGCCATGGAGGTTGCTAGTCAGGCTAGG**

**CAAATGGTGCAGGCGATGAGAACCATTGGGACTCATCCTAGCTCCAGTGCTGGTCTGAAAGACGATCTTA**

**TTGAAAATTTGCAGGCCTACCAGAAACGAATGGGGGTGCAGATGCAACGATTCAAGTGATCCTCTCGTTA**

**TTGCCGCAAGTATCATTGGGATCTTGCACTTGATATTGTGGATTCTTGATCGTCTTTTTTTCAAATGCAT**

**TTATCGTCGCCTTAAATACGGTTTGAAAAGAGGGCCTTCTACGGAAGGAGTGCCGGAGTCTATGAGGGAA**

**GAATATCGAAAGGAACAGCAGAGTGCTGTGGATGTTGACGATGGTCATTTTGTCAACATAGAGCTGGAGT**

**AAATGGATTCCAACACTGTGTCAAGCTTTCAGGTAGACTGCTTTCTTTGGCATGTCCGCAAACGGTTTGCAG**

**ACCAAGAACTGGGTGATGCCCCATTCCTTGATCGGCTTCGCCGAGATCAGAAGTCCCTAAGAGGAAGAGG**

**CAGCACTCTTGGTCTGGACATCGAGACAGCCACCCGTGCTGGAAAGCAGATAGTGGAGCGGATTCTGAAG**

**GAAGAATCCGATGAGGCACTTAAAATGACCATTGCCTCTGTACCTGCTTCGCGCTACCTAACTGACATGA**

**CTCTTGAGGAGATGTCAAGGGACTGGTTCATGCTCATGCCCAAGCAGAAAGTGGCAGGCTCTCTTTGTAT**

**CAGAATGGACCAGGCGATCATGGATAAGAACATCATACTGAAAGCGAACTTCAGTGTGATTTTCGACCGG**

**CTGGAGACTCTAATACTACTAAGGGCTTTCACCGAAGAGGGAGCAATTGTTGGCGAAATTTCACCATTGC**

**CTTCTCTTCCAGGACATACTGATGAGGATGTCAAAAATGCAGTTGGGGTCCTCATCGGAGGACTTGAATG**

**GAATGATAACACAGTTCGAGTCTCTGAAACTCTACAGAGATTCGCTTGGAGAAGCAGTAATGAGAATGGG**

**AGACCTCCACTCCCTCCAAAACAGAAACGGAAAATGGCGAGAACAATTAAGTCAGAAGTTTGAAGAAATA**

**AGATGGTTGATTGAAGAAGTGAGACATAGACTGAAGATAACAGAGAATAGTTTTGAGCAAATAACATTTA**

**TGCAAGCCTTACAACTATTGCTTGAAGTGGAGCAAGAGATAAGAACTTTCTCGTTTCAGCTTATTTAA**

**>Influenza A virus (A/Aalborg/INS132/2009(H1N1))**

**TCAAATATATTCAATATGGAGAGAATAAAAGAACTGAGAGATCTAATGTCGCAGTCCCGCACTCGCGAGA**

**TACTCACTAAGACCACTGTGGACCATATGGCCATAATCAAAAAGTACACATCAGGAAGGCAAGAGAAGAA**

**CCCCGCACTCAGAATGAAGTGGATGATGGCAATGAGATACCCAATTACAGCAGACAAGAGAATAATGGAC**

**ATGATTCCAGAGAGGAATGAACAAGGACAAACCCTCTGGAGCAAAACAAACGATGCTGGATCAGACCGAG**

**TGATGGTATCACCTCTGGCCGTAACATGGTGGAATAGGAATGGCCCAACAACAAGTACAGTTCATTACCC**

**TAAGGTATATAAAACTTATTTCGAAAAGGTCGAAAGATTGAAACATGGTACCTTCGGCCCTGTCCACTTC**

**AGAAATCAAGTTAAAATAAGGAGGAGAGTTGATACAAACCCTGGCCATGCAGATCTCAGTGCCAAGGAGG**

**CACAGGATGTGATTATGGAAGTTGTTTTCCCAAATGAAGTGGGGGCAAGAATACTGACATCAGAGTCACA**

**GCTGGCAATAACAAAAGAGAAGAAAGAAGAGCTCCAGGATTGTAAAATTGCTCCCTTGATGGTGGCGTAC**

**ATGCTAGAAAGAGAATTGGTCCGTAAAACAAGGTTTCTCCCAGTAGCCGGCGGAACAGGCAGTGTTTATA**

**TTGAAGTGTTGCACTTAACCCAAGGGACGTGCTGGGAGCAGATGTACACTCCAGGAGGAGAAGTGAGAAA**

**TGATGATGTTGACCAAAGTTTGATTATCGCTGCTAGAAACATAGTAAGAAGAGCAGCAGTGTCAGCAGAC**

**CCATTAGCATCTCTCTTGGAAATGTGCCACAGCACACAGATTGGAGGAGTAAGGATGGTGGACATCCTTA**

**GACAGAATCCAACTGAGGAACAAGCCGTAGACATATGCAAGGCAGCAATAGGGTTGAGGATTAGCTCATC**

**TTTCAGTTTTGGTGGGTTCACTTTCAAAAGGACAAGCGGATCATCAGTCAAGAAAGAAGAAGAAGTGCTA**

**ACGGGCAACCTCCAAACACTGAAAATAAGAGTACATGAAGGGTATGAAGAATTCACAATGGTTGGGAGAA**

**GAGCAACAGCTATTCTCAGAAAGGCAACCAGGAGATTGATCCAGTTGATAGTAAGCGGGAGAGACGAGCA**

**GTCAATTGCTGAGGCAATAATTGTGGCCATGGTATTCTCACAAGAGGATTGCATGATCAAGGCAGTTAGG**

**GGCGATCTGAACTTTGTCAATAGGGCAAACCAGCGACTGAACCCCATGCACCAACTCTTGAGGCATTTCC**

**AAAAAGATGCAAAAGTGCTTTTCCAGAACTGGGGAATTGAATCCATCGACAATGTGATGGGAATGATCGG**

**AATACTGCCCGACATGACCCCAAGCACGGAGATGTCGCTGAGAGGGATAAGAGTCAGCAAAATGGGAGTA**

**GATGAATACTCCAGCACGGAGAGAGTGGTAGTGAGTATTGACCGATTTTTAAGGGTTAGAGATCAAAGAG**

**GGAACGTACTATTGTCTCCCGAAGAAGTCAGTGAAACGCAAGGAACTGAGAAGTTGACAATAACTTATTC**

**GTCATCAATGATGTGGGAGATCAATGGCCCTGAGTCAGTGCTAGTCAACACTTATCAATGGATAATCAGG**

**AACTGGGAAATTGTGAAAATTCAATGGTCACAAGATCCCACAATGTTATACAACAAAATGGAATTTGAAC**

**CATTTCAGTCTCTTGTCCCTAAGGCAACCAGAAGCCGGTACAGTGGATTCGTAAGGACACTGTTCCAGCA**

**AATGCGGGATGTGCTTGGGACATTTGACACTGTCCAAATAATAAAACTTCTCCCCTTTGCTGCTGCTCCA**

**CCAGAACAGAGTAGGATGCAATTTTCCTCATTGACTGTGAATGTGAGAGGATCAGGGTTGAGGATACTGG**

**TAAGAGGCAATTCTCCAGTATTCAATTACAACAAGGCAACCAAACGACTTACAGTTCTTGGAAAGGATGC**

**AGGTGCATTGACTGAAGATCCAGATGAAGGCACATCTGGGGTGGAGTCTGCTGTCCTGAGAGGATTTCTC**

**ATTTTGGGCAAAGAAGACAAGAGATATGGCCCAGCATTAAGCATCAATGAACTGAGCAATCTTGCAAAAG**

**GAGAGAAAGCTAATGTGCTAATTGGGCAAGGGGACGTAGTGTTGGTAATGAAACGAAAGCGGGACTCTAG**

**CATACTTACTGACAGCCAGACAGCGACCAAAAGAATTCGGATGGCCATCAATTAGTGTCGAATTGTTTAA**

**AAACGACAAACCATTTGAATGGATGTCAATCCGACTCTACTTTTCCTAAAAATTCCAGCGCAAAATGCCATAAGCA**

**CCACATTCCCTTATACTGGAGATCCTCCATACAGCCATGGAACAGGAACAGGATACACCATGGACACAGT**

**AAACAGAACACACCAATACTCAGAAAAGGGAAAGTGGACGACAAACACAGAGACTGGTGCACCCCAGCTC**

**AACCCGATTGATGGACCACTACCTGAGGATAATGAACCAAGTGGGTATGCACAAACAGACTGTGTTCTAG**

**AGGCTATGGCTTTCCTTGAAGAAACCCACCCAGGAATATTTGAGAATTCATGCCTTGAAACAATGGAAGT**

**TGTTCAACAAACAAGGGTAGATAAACTAACTCAAGGTCGCCAGACTTATGATTGGACATTAAACAGAAAT**

**CAACCGGCAGCAACTGCATTGGCCAACACCATAGAAGTCTTTAGATCGAATGGCCTAACAGCTAATGAGT**

**CAGGAAGGCTAATAGATTTCTTAAAGGATGTAATGGAATCAATGAACAAAGAGGAAATAGAGATAACAAC**

**CCACTTTCAAAGAAAAAGGAGAGTAAGAGACAACATGACCAAGAAGATGGTCACGCAAAGAACAATAGGG**

**AAGAAAAAACAAAGACTGAATAAGAGAGGCTATCTAATAAGAGCACTGACATTAAATACGATGACCAAAG**

**ATGCAGAGAGAGGCAAGTTAAAAAGAAGGGCTATCGCAACACCTGGGATGCAGATTAGAGGTTTCGTATA**

**CTTTGTTGAAACTTTAGCTAGGAGCATTTGCGAAAAGCTTGAACAGTCTGGGCTCCCAGTAGGGGGCAAT**

**GAAAAGAAGGCCAAACTGGCAAATGTTGTGAGAAAGATGATGACTAATTCACAAGACACAGAGATTTCTT**

**TCACAATCACTGGGGACAACACTAAGTGGAATGAAAATCAAAATCCTCGAATGTTCCTGGCGATGATTAC**

**ATATATCACCAGAAATCAACCCGAGTGGTTCAGAAACATCCTGAGCATGGCACCCATAATGTTCTCAAAC**

**AAAATGGCAAGACTAGGGAAAGGGTACATGTTCGAGAGTAAAAGAATGAAGATTCGAACACAAATACCAG**

**CAGAAATGCTAGCAAGCATTGACCTGAAGTACTTCAATGAATCAACAAAGAAGAAAATTGAGAAAATAAG**

**GCCTCTTCTAATAGATGGCACAGCATCACTGAGTCCTGGGATGATGATGGGCATGTTCAACATGCTAAGT**

**ACGGTATTGGGAGTCTCGATACTGAATCTTGGACAAAAGAAATACACCAAGACAATATACTGGTGGGATG**

**GGCTCCAATCATCCGACGATTTTGCTCTCATAGTGAATGCACCAAACCATGAGGGTATACAAGCAGGAGT**

**GGACAGATTCTACAGGACCTGCAAGTTAGTGGGAATCAACATGAGCAAAAAGAAGTCCTATATAAATAAG**

**ACAGGGACATTTGAATTCACAAGCTTTTTTTATCGCTATGGATTTGTGGCTAATTTTAGCATGGAGCTAC**

**CCAGCTTTGGAGTGTCTGGAGTAAATGAATCAGCTGACATGAGTATTGGAGTAACAGTGATAAAGAACAA**

**CATGATAAACAATGACCTTGGACCTGCAACGGCCCAGATGGCTCTTCAATTGTTCATCAAAGACTACAGA**

**TACACATATAGGTGCCATAGGGGAGACACACAAATTCAGACGAGAAGATCATTTGAGTTAAAGAAGCTGT**

**GGGATCAAACCCAATCAAAGGTAGGGCTATTAGTATCAGATGGAGGACCAAACTTATACAATATACGGAA**

**TCTTCACATTCCTGAAGTCTGCTTAAAATGGGAGCTAATGGATGATGATTATCGGGGAAGACTTTGTAAT**

**CCCCTGAATCCCTTTGTCAGTCATAAAGAGATTGATTCTGTAAACAATGCTGTGGTAATGCCAGCCCATG**

**GTCCAGCCAAAAGCATGGAATATGATGCCGTTGCAACTACACATTCCTGGATTCCCAAGAGGAATCGTTC**

**TATTCTCAACACAAGCCAAAGGGGAATTCTTGAGGATGAACAGATGTACCAGAAGTGCTGCAATCTATTC**

**GAGAAATTTTTCCCTAGCAGTTCATATAGGAGACCGGTTGGAATTTCTAGCATGGTGGAGGCCATGGTGT**

**CTAGGGCCCGGATTGATGCCAGGGTCGACTTCGAGTCTGGACGGATCAAGAAAGAAGAGTTCTCTGAGAT**

**CATGAAGATCTGTTCCACCATTGAAGAACTCAGACGGCAAAAATAATGAATTTAACTTGTCCTTCATGAA**

**AAAATGATGGAAGACTTTGTGCGACAATGCTTCAATCCAATGATCGTCGAGCTTGCGGAAAAGGCAATGAAAGAAT**

**ATGGGGAAGATCCGAAAATCGAAACTAACAAGTTTGCTGCAATATGCACACATTTGGAAGTTTGTTTCAT**

**GTATTCGGATTTCCATTTCATCGACGAACGGGGTGAATCAATACTGGCAAAATCTGGTGACCCGAATGCA**

**TTATTGAAGCACCGATTTGAGATAATTGAAGGAAGAGACCGAATCATGGCCTGGACAGTGGTGAACAGTA**

**TATGTAACACAACAGGGGTAGAGAAGCCTAAATTTCTTCCTGATTTGTATGATTACAAAGAGAACCGGTT**

**CATTGAAATTGGAGTAACACGGAGGGAAGTCCACATATATTACCTAGAGAAAGCCAACAAAATAAAATCT**

**GAGAAGACACACATTCACATCTTTTCATTCACTGGAGAGGAGATGGCCACCAAAGCGGACTACACCCTTG**

**ACGAAGAGAGCAGGGCAAGAATCAAAACTAGGCTTTTCACTATAAGACAAGAAATGGCCAGTAGGAGTCT**

**ATGGGATTCCTTTCGTCAGTCCGAAAGAGGCGAAGAGACAATTGAAGAAAAATTTGAGATTACAGGAACT**

**ATGCGCAAGCTTGCCGACCAAAGTCTCCCACCGAACTTCTCCAGCCTTGAAAACTTTAGAGCCTATGTAG**

**ATGGATTCGAGCCGAACGGCTGCATTGAGGGCAAGCTTTCCCAAATGTCAAAAGAAGTGAACGCCAAAAT**

**TGAACCATTCTTGAGGACGACACCACGCCCCCTCAGATTGCCTGATGGGCCTCTTTGCCATCAGCGGTCA**

**AAGTTCCTGCTGATGGATGCTCTGAAATTAAGTATTGAAGACCCGAGTCACGAGGGGGAGGGAATACCAC**

**TATATGATGCAATCAAATGCATGAAGACATTCTTTGGCTGGAAAGAGCCTAACATAGTCAAACCACATGA**

**GAAAGGCATAAATCCCAATTACCTCATGGCTTGGAAGCAGGTGCTAGCAGAGCTACAGGACATTGAAAAT**

**GAAGAGAAGATCCCAAGGACAAAGAACATGAAGAGAACAAGCCAATTGAAGTGGGCACTCGGTGAAAATA**

**TGGCACCAGAAAAAGTAGACTTTGATGACTGCAAAGATGTTGGAGACCTTAAACAGTATGACAGTGATGA**

**GCCAGAGCCCAGATCTCTAGCAAGCTGGGTCCAAAATGAATTCAATAAGGCATGTGAATTGACTGATTCA**

**AGCTGGATAGAACTTGATGAAATAGGAGAAGATGTTGCCCCTATTGAACATATCGCAAGCATGAGGAGGA**

**ACTATTTTACAGCAGAAGTGTCCCACTGCAGGGCTACTGAATACATAATGAAGGGAGTGTACATAAATAC**

**GGCCTTGCTCAATGCATCCTGTGCAGCCATGGATGACTTTCAGCTGATCCCAATGATAAGCAAATGTAGG**

**ACCAAAGAAGGAAGACGGAAAACAAACCTGTATGGGTTCATTATAAAAGGAAGGTCTCATTTGAGAAATG**

**ATACTGATGTGGTGAACTTTGTAAGTATGGAGTTCTCACTCACTGACCCGAGACTGGAGCCACACAAATG**

**GGAAAAATACTGTGTTCTTGAAATAGGAGACATGCTCTTGAGGACTGCGATAGGCCAAGTGTCGAGGCCC**

**ATGTTCCTATATGTGAGAACCAATGGAACCTCCAAGATCAAGATGAAATGGGGAATGGAAATGAGGCGCT**

**GCCTTCTTCAGTCTCTTCAGCAGATTGAGAGCATGATTGAGGCCGAGTCTTCTGTCAAAGAGAAAGACAT**

**GACCAAGGAATTCTTTGAAAACAAATCGGAAACATGGCCAATCGGAGAGTCACCCAGGGGAGTGGAGGAA**

**GGCTCTATTGGGAAAGTGTGCAGGACCTTACTGGCAAAATCTGTATTCAACAGTCTATATGCGTCTCCAC**

**AACTTGAGGGGTTTTCGGCTGAATCGAGAAAATTGCTTCTCATTGTTCAGGCACTTAGGGACAACCTGGA**

**ACCTGGAACCTTCGATCTTGGGGGGCTATATGAAGCAATCGAGGAGTGCCTGATTAATGATCCCTGGGTT**

**TTGCTTAATGCATCTTGGTTCAACTCCTTCCTCACACATGCACTGAAGTAGTTGTGGCAATGCTACTATT**

**TGCTATCCATACGGAAAACAAAAGCAACAAAAATGAAGGCAATACTAGTAGTTCTGCTATATACATTTGTAACCGCAAATGC**

**AGACACATTATGTATAGGTTATCATGCGAACAATTCAACAGACACTGTAGACACAGTACTAGAAAAGAAT**

**GTAACAGTAACACACTCTGTTAACCTTCTAGAAGACAAGCATAACGGGAAACTATGCAAACTAAGAGGGG**

**TAGCCCCATTGCATTTGGGTAAATGTAACATTGCTGGCTGGATCCTGGGAAATCCAGAGTGTGAATCACT**

**CTCCACAGCAAGCTCATGGTCCTACATTGTGGAAACATCTAGTTCAGACAATGGAACGTGTTACCCAGGA**

**GATTTCATCGATTATGAGGAGCTAAGAGAGCAATTGAGCTCAGTGTCATCATTTGAAAGGTTTGAGATAT**

**TCCCCAAGACAAGTTCATGGCCCAATCATGACTCGAACAAAGGTGTAACGGCAGCATGTCCTCATGCTGG**

**AGCAAAAAGCTTCTACAAAAATTTAATATGGCTAGTTAAAAAAGGAAATTCATACCCAAAGCTCAGCAAA**

**TCCTACATTAATGATAAAGGGAAAGAAGTCCTCGTGCTATGGGGAATTCACCATCCATCTACTAGTGCTG**

**ACCAACAAAGTCTCTATCAGAATGCAGATGCATATGTTTTTGTGGGGACATCAAGATACAGCAAGAAGTT**

**CAAGCCGGAAATAGCAATAAGACCCAAAGTGAGGGATCAAGAAGGGAGAATGAACTATTACTGGACACTA**

**GTAGAGCCGGGAGACAAAATAACATTCGAAGCAACTGGAAATCTAGTGGTACCGAGATATGCATTCGCAA**

**TGGAAAGAAATGCTGGATCTGGTATTATCATTTCAGATACACCAGTCCACGATTGCAATACAACTTGTCA**

**GACACCCAAGGGTGCTATAAACACCAGCCTCCCATTTCAGAATATACATCCGATCACAATTGGAAAATGT**

**CCAAAATATGTAAAAAGCACAAAATTGAGACTGGCCACAGGATTGAGGAATGTCCCGTCTATTCAATCTA**

**GAGGCCTATTTGGGGCCATTGCCGGTTTCATTGAAGGGGGGTGGACAGGGATGGTAGATGGATGGTACGG**

**TTATCACCATCAAAATGAGCAGGGGTCAGGATATGCAGCCGACCTGAAGAGCACACAGCATGCCATTGAC**

**GAGATTACTAACAAAGTAAATTCTGTTATTGAAAAGATGAATACACAGTTCACAGCAGTAGGTAAAGAGT**

**TCAACCACCTGGAAAAAAGAATAGAGAATTTAAATAAAAAAGTTGATGATGGTTTCCTGGACATTTGGAC**

**TTACAATGCCGAACTGTTGGTTCTATTGGAAAATGAAAGAACTTTGGACTACCACGATTCAAATGTGAAG**

**AACTTATATGAAAAGGTAAGAAGCCAGTTAAAAAACAATGCCAAGGAAATTGGAAACGGCTGCTTTGAAT**

**TTTACCACAAATGCGATAACACGTGCATGGAAAGTGTCAAAAATGGGACTTATGACTACCCAAAATACTC**

**AGAGGAAGCAAAATTAAACAGAGAAGAAATAGATGGGGTAAAGCTGGAATCAACAAGGATTTACCAGATT**

**TTGGCGATCTATTCAACTGTCGCCAGTTCATTGGTACTGGTAGTCTCCCTGGGGGCAATCAGTTTCTGGA**

**TGTGCTCTAATGGGTCTCTACAGTGTAGAATATGTATTTAACATTAGGATTTCAGAAGCATGAGAAAAAC**

**ACGTAGATAATCACTCAATGAGTGACATCGAAGCCATGGCGTCTCAAGGCACCAAACGATCATATGAACAAA**

**TGGAGACTGGTGGGGAGCGCCAGGATGCCACAGAAATCAGAGCATCTGTCGGAAGAATGATTGGTGGAAT**

**CGGGAGATTCTACATCCAAATGTGCACTGAACTCAAACTCAGTGATTATGATGGACGACTAATCCAGAAT**

**AGCATAACAATAGAGAGGATGGTGCTTTCTGCTTTTGATGAGAGAAGAAATAAATACCTAGAAGAGCATC**

**CAAGTGCTGGGAAGGACCCTAAGAAAACAGGAGGACCCATATATAGAAGAATAGACGGAAAGTGGATGAG**

**AGAACTCATCCTTTATGACAAAGAAGAAATAAGGAGAGTTTGGCGCCAAGCAAACAATGGCGAAGATGCA**

**ACAGCAGGTCTTACTCATATCATGATTTGGCATTCCAACCTGAATGATGCCACATATCAGAGAACAAGAG**

**CGCTTGTTCGCACCGGAATGGATCCCAGAATGTGCTCTCTAATGCAAGGTTCAACACTTCCCAGAAGGTC**

**TGGTGCCGCAGGTGCTGCGGTGAAAGGAGTTGGAACAATAGCAATGGAGTTAATCAGAATGATCAAACGT**

**GGAATCAATGACCGAAATTTCTGGAGGGGTGAAAATGGACGAAGGACAAGGGTTGCTTATGAAAGAATGT**

**GCAATATCCTCAAAGGAAAATTTCAAACAGCTGCCCAGAGGGCAATGATGGATCAAGTAAGAGAAAGTCG**

**GAACCCAGGAAACGCTGAGATTGAAGACCTCATTTTCCTGGCACGGTCAGCACTCATTCTGAGGGGATCA**

**GTTGCACATAAATCCTGCCTGCCTGCTTGTGTGTATGGGCTTGCAGTAGCAAGTGGGCATGACTTTGAAA**

**GGGAAGGGTACTCACTGGTCGGGATAGACCCATTCAAATTACTCCAAAACAGCCAAGTGGTCAGCCTGAT**

**GAGACCAAATGAAAACCCAGCTCACAAGAGTCAATTGGTGTGGATGGCATGCCACTCTGCTGCATTTGAA**

**GATTTAAGAGTATCAAGTTTCATAAGAGGAAAGAAAGTGATTCCAAGAGGAAAGCTTTCCACAAGAGGGG**

**TCCAGATTGCTTCAAATGAGAATGTGGAAACCATGGACTCCAATACCCTGGAACTAAGAAGCAGGTACTG**

**GGCCATAAGGACCAGGAGTGGAGGAAATACCAATCAACAAAAGGCATCCGCAGGCCAGATCAGTGTGCAG**

**CCTACATTCTCAGTGCAGCGAAATCTCCCTTTTGAAAGAGCAACCGTTATGGCAGCATTCAGCGGGAACA**

**ATGAAGGACGGACATCCGACATGCGAACAGAAGTTATAAGAATGATGGAAAGTGCAAAGCCAGAAGATTT**

**GTCCTTCCAGGGGCGGGGGGTCTTCGAGCTCTCGGACGAAAAGGCAACGAACCCGATCGTGCCTTCCTTT**

**GACATGAGTAATGAAGGGTCTTATTTCTTCGGAGACAATGCAGAGGAGTATGACAGTTGAGGAAAAATAC**

**AGTTTAAAATGAATCCAAACCAAAAGATAATAACCATTGGTTCGGTCTGTATGACAATTGGAATGGCTAA**

**CTTAATATTACAAATTGGAAACATAATCTCAATATGGATTAGCCACTCAATTCAACTTGGGAATCAAAAT**

**CAGATTGAAACATGCAATCAAAGCGTCATTACTTATGAAAACAACACTTGGGTAAATCAGACATATGTTA**

**ACATCAGCAACACCAACTTTGCTGCTGGACAGTCAGTGGTTTCCGTGAAATTAGCGGGCAATTCCTCTCT**

**CTGCCCTGTTAGTGGATGGGCTATATACAGTAAAGACAACAGTATAAGAATCGGTTCCAAGGGGGATGTG**

**TTTGTCATAAGGGAACCATTCATATCATGCTCCCCCTTGGAATGCAGAACCTTCTTCTTGACTCAAGGGG**

**CCTTGCTAAATGACAAACATTCCAATGGAACCATTAAAGACAGGAGCCCATATCGAACCCTAATGAGCTG**

**TCCTATTGGTGAAGTTCCCTCTCCATACAACTCAAGATTTGAGTCAGTCGCTTGGTCAGCAAGTGCTTGT**

**CATGATGGCATCAATTGGCTAACAATTGGAATTTCTGGCCCAGACAATGGGGCAGTGGCTGTGTTAAAGT**

**ACAACGGCATAATAACAGACACTATCAAGAGTTGGAGAAACAATATATTGAGAACACAAGAGTCTGAATG**

**TGCATGTGTAAATGGTTCTTGCTTTACTGTAATGACCGATGGACCAAGTGATGGACAGGCCTCATACAAG**

**ATCTTCAGAATAGAAAAGGGAAAGATAGTCAAATCAGTCGAAATGAATGCCCCTAATTATCACTATGAGG**

**AATGCTCCTGTTATCCTGATTCTAGTGAAATCACATGTGTGTGCAGGGATAACTGGCATGGCTCGAATCG**

**ACCGTGGGTATCTTTCAACCAGAATCTGGAATATCAGATAGGATACATATGCAGTGGGATTTTCGGAGAC**

**AATCCACGCCCTAATGATAAGACAGGCAGTTGTGGTCCAGTATCGTCTAATGGAGCAAATGGAGTAAAAG**

**GATTTTCATTCAAATACGGCAATGGTGTTTGGATAGGGAGAACTAAAAGCATTAGTTCAAGAAACGGTTT**

**TGAGATGATTTGGGATCCGAACGGATGGACTGGGACAGACAATAACTTCTCAATAAAGCAAGATATCGTA**

**GGAATAAATGAGTGGTCAGGATATAGCGGGAGTTTTGTTCAGCATCCAGAACTAACAGGGCTGGATTGTA**

**TAAGACCTTGCTTCTGGGTTGAACTAATCAGAGGGCGACCCAAAGAGAACACAATCTGGACTAGCGGGAG**

**CAGCATATCCTTTTGTGGTGTAAACAGTGACACTGTGGGTTGGTCTTGGCCGGACGGTGCTGAGTTGCCA**

**TTTACCATTGACAAGTAATTTGTTCAAAAAACTTAGATATTTAAAGATGAGTCTTCTAACCGAGGTCGAA**

**ACGTACGTTCTTTCTATCATCCCGTCAGGCCCC**

**CTCAAAGCCGAGATCGCGCAGAGACTGGAAAGTGTCTTTGCAGGAAAGAACACAGATCTTGAGGCTCTCA**

**TGGAATGGCTAAAGACAAGACCAATCTTGTCACCTCTGACTAAGGGAATTTTAGGATTTGTGTTCACGCT**

**CACCGTGCCCAGTGAGCGAGGACTGCAGCGTAGACGCTTTGTCCAAAATGCCCTAAATGGGAATGGGGAC**

**CCGAACAACATGGATAGAGCAGTTAAACTATACAAGAAGCTCAAAAGAGAAATAACGTTCCATGGGGCCA**

**AGGAGGTGTCACTAAGCTATTCAACTGGTGCACTTGCCAGTTGCATGGGCCTCATATACAACAGGATGGG**

**AACAGTGACCACAGAAGCTGCTTTTGGTCTAGTGTGTGCCACTTGTGAACAGATTGCTGATTCACAGCAT**

**CGGTCTCACAGACAAATGGCTACTACCACCAATCCACTAATCAGGCATGAAAACAGAATGGTGCTGGCTA**

**GCACTACGGCAAAGGCTATGGAACAGATGGCTGGATCGAGTGAACAGGCAGCAGAGGCCATGGAGGTTGC**

**TAATCAGACTAGGCAGATGGTACATGCAATGAGAACTATTGGGACTCATCCTAGCTCCAGTGCTGGTCTG**

**AAAGATGACCTTCTTGAAAATTTGCAGGCCTACCAGAAGCGAATGGGAGTGCAGATGCAGCGATTCAAGT**

**GATCCTCTCGTCATTGCAGCAAATATCATTGGGATCTTGCACCTGATATTGTGGATTACTGATCGTCTTT**

**TTTTCAAATGTATTTATCGTCGCTTTAAATACGGTTTGAAAAGAGGGCCTTCTACGGAAGGAGTGCCTGA**

**GTCCATGAGGGAAGAATATCAACAGGAACAGCAGAGTGCTGTGGATGTTGACGATGGTCATTTTGTCAAC**

**ATAGAGCTAGAGTAAAAAACTAGTGACAAAAACATAATGGACTCCAACACCATGTCAAGCTTTCAGGTAG**

**ACTGTTTCCTTTGGCATATCCG**

**CAAGCGATTTGCAGACAATGGATTGGGTGATGCCCCATTCCTTGATCGGCTCCGCCGAGATCAAAAGTCC**

**TTAAAAGGAAGAGGCAACACCCTTGGCCTCGATGTCGAAACAGCCACTCTTGTTGGGAAACAAATCGTGG**

**AATGGATCTTGAAAGAGGAATCCAGCGAGACACTTAGAATGACAATTGCATCTGTACCTACTTCGCGCTA**

**CCTTTCTGACATGACCCTCGAGGAAATGTCACGAGACTGGTTCATGCTCATGCCTAGGCAAAAGATAATA**

**GGCCCTCTTTGCGTGCGATTGGACCAGGCGGTCATGGAAAAGAACATAGTACTGAAAGCGAACTTCAGTG**

**TAATCTTTAACCGATTAGAGACCTTGATACTACTAAGGGCTTTCACTGAGGAGGGAGCAATAGTTGGAGA**

**AATTTCACCATTACCTTCTCTTCCAGGACATACTTATGAGGATGTCAAAAATGCAGTTGGGGTCCTCATC**

**GGAGGACTTGAATGGAATGGTAACACGGTTCGAGTCTCTGAAAATATACAGAGATTCGCTTGGAGAAACT**

**GTGATGAGAATGGGAGACCTTCACTACCTCCAGAGCAGAAATGAAAAGTGGCGAGAGCAATTGGGACAGA**

**AATTTGAGGAAATAAGGTGGTTAATTGAAGAAATGCGGCACAGATTGAAAGCGACAGAGAATAGTTTCGA**

**ACAAATAACATTTATGCAAGCCTTACAACTACTGCTTGAAGTAGAACAAGAGATAAGAGCTTTCTCGTTT**

**CAGCTTATTTAATGATAAAAAACAC**

**>Influenza A virus (A/Albany/1/1958(H2N2))**

**CAATATGGAAAGAATAAAAGAACTACGGAATCTGATGTCGCAGTCTCGCACTCGCGAGATACTAACAAAA**

**ACCACAGTGGACCATATGGCCATAATTAAGAAGTACACATCAGGGAGACAGGAAAAGAACCCGTCACTTA**

**GGATGAAATGGATGATGGCAATGAAATATCCGATTACAGCTGACAAGAGGATAACAGAAATGATTCCTGA**

**GAGAAATGAGCAAGGGCAAACTCTATGGAGTAAAATGAGTGATGCCGGATCGGATCGAGTGATGGTATCA**

**CCTCTGGCTGTGACATGGTGGAATAGAAATGGACCAATGACAAGTACGGTTCATTATCCAAAAATCTACA**

**AAACTTATTTTGAGAAAGTCGAAAGGTTGAAACATGGAACCTTTGGCCCTGTCCATTTTAGAAACCAAGT**

**CAAAATACGCCGAAGAGTTGACATAAATCCTGGTCATGCAGACCTCAGTGCCAAGGAGGCACAGGATGTA**

**ATCATGGAAGTTGTTTTCCCTAACGAAGTGGGGGCCAGGATACTAACGTCGGAATCGCAATTAACAATAA**

**CCAAAGAGAAAAAAGAAGAACTCCAGGATTGCAAAATTTCTCCTTTGATGGTTGCGTACATGTTAGAGAG**

**AGAACTTGTCCGAAAAACGAGATTTCTCCCAGTTGCTGGTGGAACAAGCAGTGTGTACATTGAAGTGTTG**

**CACTTGACTCAAGGAACATGCTGGGAACAGATGTACACTCCAGGTGGAGAAGTGAGGAATGATGATGTTG**

**ATCAAAGTCTAATTATTGCAGCCAGGAACATAGTGAGAAGAGCAGCAGTATCAGCAGATCCACTAGCATC**

**TTTATTGGAGATGTGCCACAGCACACAGATTGGCGGGACAAGGATGGTGGACATTCTTAGGCAGAACCCA**

**ACGGAAGAGCAAGCTGTGGATATATGCAAGGCTGCAATGGGACTGAAAATCAGCTCATCCTTCAGTTTTG**

**GCGGGTTCACATTTAAGAGAACAAGCGGATCATCAGTCAAGAGAGAGGAAGAAGTGCTTACGGGCAATCT**

**TCAAACATTGAAAATAAGGGTGCATGAGGGATACGAGGAGTTCACAATGGTTGGGAAAAGGGCAACAGCT**

**ATACTCAGAAAAGCAACCAGGAGATTGATTCAGCTGATAGTGAGTGGAAGAGACGAACAGTCGATAGCCG**

**AAGCAATAATTGTGGCCATGGTATTTTCACAAGAAGATTGTATGATAAAAGCAGTTAGAGGTGATCTGAA**

**TTTCGTTAATAGGGCAAATCAGCGATTGAATCCCATGCATCAACTTTTAAGACATTTTCAGAAGGATGCG**

**AAAGTGCTTTTTCAAAATTGGGGAATTGAACATATCGACAATGTGATGGGAATGATTGGGGTATTACCAG**

**ACATGACTCCAAGCACAGAGATGTCAATGAGAGGGGTAAGAGTCAGCAAAATGGGCGTAGATGAATACTC**

**CAGCGCGGAGAGAGTAGTGGTGAGCATTGACCGGTTTTTGAGAGTTCGAGACCAACGAGGAAATGTACTA**

**CTATCTCCTGAGGAGGTCAGTGAAACACAGGGAACAGAGAAACTGACAATAACTTACTCATCGTCAATGA**

**TGTGGGAGATTAATGGCCCTGAGTCAGTGTTGGTCAATACCTATCAGTGGATCATCAGAAACTGGGAAAC**

**TGTTAAAATTCAGTGGTCTCAGAATCCTACAATGCTATACAATAAAATGGAATTTGAGCCATTTCAGTCT**

**TTAGTTCCTAAGGCCATTAGAGGCCAATACAGTGGGTTTGTTAGGACTCTATTCCAACAAATGAGGGATG**

**TACTTGGGACATTTGATACCACCCAGATAATAAAACTTCTTCCCTTTGCAGCCGCCCCACCAAAGCAAAG**

**TAGAATGCAGTTCTCTTCATTGACTGTGAATGTGAGGGGATCAGGAATGAGAATACTTGTAAGGGGCAAT**

**TCTCCTGTATTCAACTACAACAAGACCACTAAGAGACTAACAATTCTCGGAAAGGATGCTGGCACTTTAA**

**CTGAAGACCCAGATGAAGGCACATCTGGAGTGGAGTCCGCTGTTCTGAGAGGATTCCTCATTCTGGGCAA**

**AGAAGATAGGAGATATGGACCAGCATTAAGCATCAATGAACTGAGTAACCTTGCGAAAGGAGAAAAGGCT**

**AATGTACTAATTGGGCAAGGAGACGTGGTGTTGGTAATGAAACGAAAACGGGACTCTAGCATACTTACTG**

**ACAGCCAGACAGCGACCAAAAGAATTCGGATGGCCATCAATTAATGTTGAATAGTTTAAAAACGTGAATG**

**GATGTCAATCCGACCTTACTTTTCTTGAAAGTTCCAGCGCAAAATGCCATAAGTACTACATTCC**

**CTTATACTGGAGATCCTCCATACAGCCATGGAACAGGAACAGGATACACCATGGACACAGTCAACAGAAC**

**ACATCAATATTCAGAAAAGGGGAAGTGGACAACAAACACGGAAACTGGAGCGCCCCAACTTAACCCAATT**

**GATGGACCACTACCTGAGGACAATGAACCAAGTGGATATGCACAAACAGACTGCGTCCTGGAAGCAATGG**

**CTTTCCTTGAAGAATCCCACCCAGGAATCTTTGAAAACTCGTGTCTTGAAACGATGGAAGTTATTCAACA**

**AACAAGAGTGGACAAACTGACCCAAGGTCGTCAGACCTATGATTGGACATTGAACAGAAATCAGCCGGCT**

**GCAACTGCGCTAGCCAACACTATAGAGGTCTTCAGATCGAATGGTCTGACAGCTAATGAATCGGGAAGGC**

**TAATAGATTTCCTCAAGGATGTGATAGAATCAATGGATAAAGAGGAGATGGAAATAACAACACACTTCCA**

**AAGAAAAAGAAGAGTAAGAGACAACATGACCAAGAAAATGGTCACACAACGAACAATAGGAAAGAAGAAG**

**CAAAGATTGGACAAGAGAAGCTATCTAATAAGAGCACTGACATTGAACACAATGACTAAAGATGCAGAGA**

**GAGGTAAATTAAAGAGAAGAGCAATTGCAACACCCGGTATGCAGATCAGAGGGTTCGTGTACTTTGTCGA**

**AACACTAGCGAGAAGTATTTGTGAGAAGCTTGAACAGTCTGGGCTTCCGGTTGGAGGTAATGAAAAGAAG**

**GCTAAACTGGCAAATGTTGTGAGAAAAATGATGACTAATTCACAAGACACAGAGCTCTCTTTCACAATTA**

**CTGGAGACAATACCAAATGGAATGAGAATCAAAATCCTCGGATGTTCCTGGCGATGATAACATACATCAC**

**AAGAAATCAACCTGAATGGTTTAGAAACGTCCTGAGCATCGCACCTATAATGTTCTCAAATAAAATGGCA**

**AGACTAGGGAAAGGATACATGTTCGAAAGCAAGAGCATGAAGCTCCGAACACAAATACCAGCAGAAATGC**

**TAGCAAGTATTGACCTGAAATACTTTAATGAATCAACAAGAAAGAAAATCGAGAAAATAAGGCCTCTCCT**

**AATAGATGGCACAGTCTCATTGAGTCCTGGAATGATGATGGGCATGTTCAACATGCTAAGTACAGTCTTA**

**GGAGTCTCAATCCTGAATCTTGGACAAAAGAAGTACACCAAAACAACATACTGGTGGGACGGACTCCAAT**

**CCTCTGATGACTTCGCCCTCATAGTGAATGCACCAAATCATGAGGGAATACAAGCAGGGGTGGATAGATT**

**CTACAGAACCTGCAAGCTAGTCGGAATCAATATGAGCAAAAAGAAGTCCTACATAAATAGGACAGGGACA**

**TTTGAATTCACAAGCTTTTTCTATCGCTATGGATTTGTAGCCAATTTTAGCATGGAGCTGCCCAGCTTTG**

**GGGTGTCTGGAATTAATGAATCGGCTGATATGAGCATTGGGGTAACAGTGATAAAGAACAACATGATAAA**

**CAATGACCTTGGGCCAGCAACAGCCCAAATGGCTCTTCAACTATTCATCAAAGACTACAGATATACGTAC**

**CGGTGCCACAGAGGAGACACACAAATTCAGACAAGGAGATCATTCGAGCTAAAGAAGCTGTGGGAGCAAA**

**CCCGCTCAAAGGCAGGACTTTTGGTTTCGGATGGAGGACCAAACTTATACAATATCCGGAATCTCCACAT**

**TCCAGAAGTCTGCTTGAAGTGGGAGCTAATGGATGAAGACTATCAGGGGAGGCTTTGTAATCCCCTGAAT**

**CCATTTGTCAGTCATAAGGAGATTGAGTCTATAAACAATGCTGTGGTAATGCCAGCTCACGGTCCAGCCA**

**AGAGCATGGAATATGATGCTGTTGCTACTACACACTCCTGGATCCCTAAGAGGAACCGCTCCATTCTCAA**

**CACAAGCCAAAGGGGAATTCTTGAGGATGAACAGATGTATCAAAAGTGTTGCAATCTATTCGAGAAATTC**

**TTCCCTAGCAGTTCGTACAGGAGACCAGTTGGAATTTCCAGCATGGTGGAGGCCATGGTGTCTAGGGCCC**

**GGATTGATGCACGGATTGACTTCGAGTCTGGACGGATTAAGAAAGAGGAGTTCGCTGAGATCATGAAGAT**

**CTGTTCCACCATTGAAGAGCTCAGACGGCAAAAATAGTGAATTTAGCTTGTCCTTCATGAAAAAAATGGA**

**AGATTTTGTGCGACAATGCTTCAATCCGATGATTGTCGAGCTTGCGGAAAAGGCAATGAAAGA**

**GTATGGAGAGGATCTGAAAATCGAAACAAACAAATTTGCAGCAATATGCACTCACTTGGAAGTATGCTTC**

**ATGTATTCAGATTTTCATTTCATCAATGAGCAAGGCGAGTCAATAATAGTAGAGCTTGATGATCCAAATG**

**CACTTTTGAAGCACAGATTTGAAATAATAGAGGGAAGAGATCGCACAATGGCCTGGACAGTAGTAAACAG**

**TATTTGCAACACTACAGGAGCTGAGAAACCGAAGTTTCTGCCAGATTTGTATGATTACAAGGAGAATAGA**

**TTCATCGAGATTGGAGTGACAAGGAGGGAAGTCCACATATACTATCTTGAAAAAGCCAATAAAATTAAAT**

**CTGAGAAGACACACATCCACATTTTCTCATTCACTGGGGAAGAAATGGCCACAAAGGCCGACTACACTCT**

**CGATGAGGAAAGCAGGGCTAGGATCAAAACCAGACTATTCACCATAAGACAAGAAATGGCTAGCAGAGGC**

**CTCTGGGATTCCTTTCGTCAGTCCGAAAGAGGCGAAGAAACAATTGAAGAAAGATTTGAAATCACAGGGA**

**CAATGCGCAGGCTCGCCGACCAAAGTCTCCCGCCGAACTTCTCCTGCATTGAGAATTTTAGAGCCTATGT**

**GGATGGATTCGAACCGAACGGCTACATTGAGGGCAAGCTTTCTCAAATGTCCAAAGAAGTAAATGCTAAA**

**ATTGAACCTTTTCTGAAAACAACACCAAGACCAATTAGACTTCCGGATGGGCCTCCTTGTTCTCAGCGGT**

**CCAAATTCCTGCTGATGGATGCTTTAAAATTAAGCATTGAGGACCCAAGTCACGAAGGAGAGGGAATACC**

**ACTATATGATGCGATCAAGTGTATGAGAACATTCTTTGGATGGAAAGAACCCTATGTTGTTAAACCACAC**

**GAAAAGGGAATAAATCCAAATTATCTGCTGTCATGGAAGCAAGTACTGGCAGAACTGCAGGACATTGAGA**

**ATGAGGAGAAGATTCCAAGAACCAAAAACATGAAGAAAACGAGTCATCTAAAGTGGGCACTTGGTGAGAA**

**CATGGCACCAGAGAAGGTAGACTTTGACGACTGTAGAGATATAAGCGATTTGAAGCAATATGATAGTGAT**

**GAACCTGAATTAAGGTCACTTTCAAGCTGGATCCAGAATGAGTTCAACAAGGCATGCGAGCTGACCGATT**

**CAATTTGGATAGAGCTCGATGAGATTGGAGAAGATGTGGCTCCAATTGAACACATTGCAAGCATGAGAAG**

**GAATTACTTCACAGCAGAGGTGTCTCATTGCAGAGCCACAGAATATATAATGAAGGGGGTATACATTAAT**

**ACTGCCTTGCTTAATGCATCCTGTGCAGCAATGGACGATTTCCAACTAATTCCCATGATAAGCAAATGTA**

**GAACTAAAGAGGGAAGGCGAAAGACCAATTTATATGGTTTCATCATAAAAGGAAGATCTCACTTAAGGAA**

**TGACACCGACGTGGTAAACTTTGTGAGCATGGAGTTTTCTCTCACTGACCCAAGACTTGAGCCACACAAA**

**TGGGAGAAGTACTGTGTTCTTGAGATAGGAGATATGCTACTAAGAAGTGCCATAGGCCAGGTGTCAAGGC**

**CCATGTTCTTGTATGTGAGGACAAATGGAACATCAAAGATTAAAATGAAATGGGGAATGGAGATGAGGCG**

**TTGCCTCCTTCAGTCGCTCCAACAAATCGAGAGTATGATTGAAGCCGAGTCCTCTGTCAAGGAGAAAGAC**

**ATGACCAAAGAGTTTTTCGAGAATAAATCAGAAACATGGCCCATTGGAGAGTCCCCCAAAGGAGTGGAAG**

**AAGGTTCCATTGGGAAGGTCTGCAGGACTTTATTAGCCAAGTCGGTATTCAATAGCCTGTATGCATCTCC**

**ACAATTGGAAGGATTTTCAGCTGAATCAAGAAAACTGCTTCTTGTCGTTCAGGCTCTTAGGGACAATCTT**

**GAACCTGGGACCTTTGATCTTGGGGGGCTATATGAAGCAATTGAGGAGTGCCTGATTAATGATCCCTGGG**

**TTTTGCTTAATGCGTCTTGGTTCAACTCCTTCCTAACACATGCATTAAGATAGTTGTGGCAATGCTACTA**

**TTTGCTATCCATACTGTCCAAAATGGCCATCATTTATCTCATTCTCCTGTTCACAGCAGTGAGAGGGGAC**

**CAGATATGCATTGGATACCATG**

**CCAATAATTCCACAGAGAAGGTCGACACAATTCTAGAGCGGAACGTCACTGTGACTCATGCCAAGGACAT**

**TCTTGAGAAGACCCATAACGGAAAGTTATGCAAACTAAACGGAATCCCTCCACTTGAACTAGGGGACTGT**

**AGCATTGCCGGATGGCTCCTTGGAAATCCAGAATGTGATAGGCTTCTAAGTGTGCCAGAATGGTCCTATA**

**TAATGGAGAAAGAAAACCCGAGAAACGGTTTGTGTTATCCAGGCAGCTTCAATGATTATGAAGAATTGAA**

**ACATCTCCTCAGCAGCGTGAAACATTTCGAGAAAGTAAAGATTCTGCCCAAAGATAGATGGACACAGCAT**

**ACAACAACTGGAGGTTCACAGGCCTGTGCGGTGTCTGGTAATCCATCATTCTTCAGGAACATGGTCTGGC**

**TGACAAAGAAAGGATCAGATTATCCGGTTGCCAAAGGATCGTACAACAATACAAGCGGAGAACAAATGCT**

**AATAATTTGGGGGGTGCACCATCCCATTGATGAGACAGAACAAAGAACATTGTACCAGAATGTGGGAACC**

**TATGTTTCCGTAGGCACATCAACATTGAACAAAAGGTCAACCCCAGAAATAGCAACAAGGCCTAAAGTGA**

**ATGGACTAGGGAGTAGAATGGAATTCTCTTGGACCCTCTTGGATATGTGGGACACCATAAATTTTGAGAG**

**TACTGGTAATCTAATTGCACCAGAGTATGGATTCAAAATATCGAAAAGAGGTAGTTCAGGGATCATGAAA**

**ACAGAAGGAACACTTGAGAACTGTGAGACCAAATGCCAAACTCCTTTGGGAGCAATAAATACAACATTGC**

**CTTTTCACAATGTCCACCCACTGACAATAGGTGAGTGCCCCAAATATGTAAAATCGGAGAAGTTGGTCTT**

**AGCAACAGGACTAAGGAATGTTCCCCAGATTGAATCAAGAGGATTGTTTGGGGCAATAGCTGGTTTTATA**

**GAAGGAGGATGGCAAGGAATGGTTGATGGTTGGTATGGATACCATCACAGCAATGACCAGGGATCAGGGT**

**ATGCAGCAGACAAAGAATCCACTCAAAAGGCATTTGATGGAATCACCAACAAGGTAAATTCTGTGATTGA**

**AAAGATGAACACCCAATTTGAAGCTGTTGGGAAAGAATTCGGTAACTTAGAGAGAAGACTGGAGAACTTG**

**AACAAAAAGATGGAAGACGGGTTTCTAGATGTGTGGACATACAATGCTGAGCTTCTAGTTCTGATGGAAA**

**ATGAGAGGACACTTGACTTTCATGATTCTAATGTCAAGAATCTGTATGATAAAGTCAGAATGCAGCTGAG**

**AGACAACGTCAAAGAACTAGGAAATGGATGTTTTGAATTTTATCACAAATGTGATGATGAATGCATGAAT**

**AGTGTGAAAAACGGGACGTATGATTATCCCAAGTATGAAGAAGAGTCTAAACTAAATAGAAATGAAATCA**

**AAGGGGTAAAATTGAGCAGCATGGGGGTTTATCAAATCCTTGCCATTTATGCTACAGTAGCAGGTTCTCT**

**GTCACTGGCAATCATGATGGCTGGGATCTCTTTCTGGATGTGCTCCAACGGGTCTCTGCAGTGCAGGATC**

**TGCATATGACTCACTGAGTGACATCAAAATCATGGCGTCCCAAGGCACCAAACGGTCTTATGAACAGATG**

**GAAACTGAT**

**GGGGAACGCCAGAATGCAACTGAAATCAGAGCATCCGTCGGGAAGATGATTGATGGAATTGGACGATTCT**

**ACATCCAAATGTGCACCGAACTTAAACTCAGTGATTATGAGGGGCGGCTGATCCAGAACAGCTTAACAAT**

**AGAGAGAATGGTGCTCTCTGCTTTTGACGAGAGGAGGAATAAATATCTGGAAGAACATCCCAGCGCGGGG**

**AAGGATCCTAAGAAAACTGGAGGACCCATATACAAGAGAGTAGATGGAAAGTGGATGAGGGAACTCGTCC**

**TTTATGACAAAGAAGAAATAAGGCGAATCTGGCGCCAAGCTAATAATGGTGATGATGCAACAGCTGGTCT**

**GACTCACATGATGATCTGGCATTCCAATTTGAATGATACAACATACCAGAGAACAAGAGCTCTTGTTCGC**

**ACCGGAATGGATCCCAGGATGTGCTCTTTGATGCAGGGTTCGACTCTCCCTAGGAGGTCTGGAGCCGCAG**

**GCGCTGCAGTCAAAGGAGTTGGGACAATGGTGATGGAGTTGATCAGGATGATCAAACGTGGGATCAATGA**

**TCGGAACTTCTGGAGAGGTGAGAATGGGCGGAAAACAAGGACTGCTTATGAGAGAATGTGCAACATTCTC**

**AAAGGAAAATTTCAAACAGCTGCACAAAGAGCAATGATGGATCAAGTGAGAGAAAGCCGGAACCCAGGAA**

**ATGCTGAGATCGAAGATCTCATCTTTCTGGCACGGTCTGCACTCATATTGAGAGGGTCAGTTGCTCACAA**

**ATCTTGTCTGCCTGCCTGTGTGTATGGACCTGCCGTAGCCAGTGGGTACGACTTCGAAAAAGAGGGATAC**

**TCTTTAGTAGGGATAGACCCTTTCAAATTGCTTCAAAACAGCCAAGTATACAGCCTAATCAGACCGAACG**

**AGAATCCAGCACACAAGAGTCAGCTGGTGTGGATGGCATGCAATTCTGCTGCATTTGAAGATCTAAGAGT**

**ATCAAGCTTCATCAGAGGGACCAAAGTAATCCCAAGGGGGAAACTTTCCACTAGAGGAGTACAAATTGCT**

**TCAAATGAAAACATGGATACTATGGAATCAAGTACTCTTGAACTGAGAAGCAGGTACTGGGCCATAAGGA**

**CCAGAAGTGGAGGAAACACTAATCAACAGAGGGCCTCTGCAGGTCAAATCAGTGTACAACCTACGTTTTC**

**TGTGCAAAGAAACCTCCCATTTGACAAACCAACCATCATGGCAGCATTCACTGGGAATGCAGAGGGAAGA**

**ACATCAGACATGAGGGCAGAAATCATAAGGATGATGGAAGGTGCAAAACCAGAAGAAGTGTCCTTCCAGG**

**GGCGGGGAGTCTTCGAGCTCTCGGACGAAAAGGCAACGAACCCGATCGTGCCCTCTTTTGACATGAGTAA**

**TGAAGGATCTTATTTCTTCGGAGACAATGCAGAGGAGTACGACAATTAAGGAATGAAAATGAATCCAAAT**

**CAAAAGATAATAACAATTGGCTCTGTCTCTCTCACCATTGCAACAGTATGCTT**

**CCTCATGCAGATTGCCATCCTGGCAACTACTGTGACATTGCATTTTAAACAACATGAGTGCGACTCCCCC**

**GCGAGCAACCAAGTAATGCCATGTGAACCAATAATAATAGAAAGGAACATAACAGAGATAGTGTATTTGA**

**ATAACACCACCATAGAGAAAGAGATTTGCCCCGAAGTAGTGGAATACAGAAATTGGTCAAAGCCGCAATG**

**TCAAATTACAGGATTTGCACCTTTTTCTAAGGACAATTCAATCCGGCTTTCTGCTGGTGGGGACATTTGG**

**GTGACGAGAGAACCTTATGTGTCATGCGATCCTGGCAAGTGTTACCAATTTGCACTCGGGCAGGGGACCA**

**CACTAGACAACAAACATTCAAATGGCACAATACATGATAGAATCCCTCACCGAACCCTATTAATGAATGA**

**GTTGGGTGTTCCATTTCATTTAGGAACCAAACAAGTGTGTGTAGCATGGTCCAGCTCAAGTTGTCACGAT**

**GGAAAAGCATGGTTGCATGTTTGTGTCACTGGGGATGATAGAAATGCGACTGCTAGCTTCATTTATGACG**

**GGAGGCTTGTGGACAGTATTGGTTCATGGTCTCAAAATATCCTCAGGACCCAGGAGTCGGAATGCGTTTG**

**TATCAATGGGACTTGCACAGTAGTAATGACTGATGGAAGTGCATCAGGAAGAGCCGATACTAGAATACTA**

**TTCATTAAAGAGGGGAAAATTGTCCATATTAGCCCATTGTCAGGAAGTGCTCAGCATATAGAGGAGTGTT**

**CCTGTTACCCTCGATATCCTGACGTCAGATGTATCTGCAGAGACAACTGGAAAGGCTCTAATAGGCCCGT**

**TATAGACATAAATATGGAAGATTATAGCATTGATTCCAGTTATGTGTGCTCAGGGCTTGTTGGCGACACA**

**CCCAGGAACGACGACAGCTCTAGCAATAGCAATTGCAGGGATCCTAACAATGAGAGAGGGAATCCAGGAG**

**TGAAAGGCTGGGCCTTTGACAATGGAGATGATGTATGGATGGGAAGAACAATCAACAAAGATTCACGCTC**

**AGGTTATGAAACTTTCAAAGTCATTGGTGGTTGGTCCACACCTAATTCCAAATCGCAGGTCAATAGACAG**

**GTCATAGTTGACAACAATAATTGGTCTGGTTACTCTGGTATTTTCTCTGTTGAGGGCAAAAGCTGCATCA**

**ATAGGTGCTTTTATGTGGAGTTGATAAGGGGAAGGCCACAGGAGACTAGAGTATGGTGGACCTCAAACAG**

**TATTGTTGTGTTTTGTGGCACTTCAGGTACTTATGGAACAGGCTCATGGCCTGATGGGGCGAACATCAAT**

**TTCATGCCTATATAAGCTTTCGCAATTTTAGAAAATATTGAAAGATGAGTCTTCTAACCGAGGTCGAAAC**

**GTACGTTCTCTCTATCGTCCCGTCAGGCCCCCTC**

**AAAGCCGAGATCGCACAGAGACTTGAAGATGTCTTTGCTGGGAAGAACACCGATCTTGAGGCTCTCATGG**

**AATGGCTAAAGACAAGACCAATCCTGTCACCTCTGACTAAGGGGATTTTGGGATTTGTATTCACGCTCAC**

**CGTGCCCAGTGAGCGAGGACTGCAGCGTAGACGCTTTGTCCAAAATGCCCTCAATGGGAATGGGGATCCA**

**AATAACATGGACAGAGCAGTTAAACTGTATAGAAAGCTTAAGAGGGAGATAACATTCCATGGGGCCAAAG**

**AAATAGCGCTCAGTTATTCTGCTGGTGCACTTGCCAGTTGTATGGGCCTCATATACAACAGGATGGGGGC**

**TGTGACCACTGAAGTGGCCTTTGGCCTGGTATGTGCAACCTGTGAACAGATTGCTGACTCCCAGCATAGG**

**TCTCATAGGCAAATGGTGACAACAACCAATCCACTAATAAGACATGAGAACAGAATGGTTCTGGCCAGCA**

**CTACAGCTAAGGCTATGGAGCAAATGGCTGGATCGAGTGAGCAAGCAGCAGAGGCCATGGAAGTTGCTAG**

**TCAGGCCAGGCAAATGGTGCAGGCAATGAGAGCCATTGGGACTCATCCTAGCTCCAGTGCTGGTCTAAAA**

**GATGATCTTCTTGAAAATTTGCAGGCCTATCAGAAACGAATGGGGGTGCAGATGCAACGATTCAAGTGAC**

**CCTCTTGTTGTTGCCGCGAGTATCATTGGGATCTTGCACTTGATATTGTGGATTCTTGATCGTCTTTTTT**

**TCAAATGCATTTATCGCTTCTTTAAACACGGTCTGAAAAGAGGGCCTTCTACGGAAGGAGTACCAGAGTC**

**TATGAGGGAAGAATATCGAAAGGAACAGCAGAGTGCTGTGGATGCTGACGATAGTCATTTTGTCAGCATA**

**GAGCTGGAGTAAAACATAATGGATCCTAACACTGTGTCAAGCTTTCAGGTAGATTGCTTCCTTTGGCATG**

**TCCGCAAACAAGT**

**TGCAGACCAAGAACTAGGTGATGCCCCATTCCTTGATCGGCTTCGCCGAGATCAGAAGTCCCTAAGGGGA**

**AGAGGCAGTACTCTCGGTCTGAACATCGAAACAGCCACCCGTGTTGGAAAGCAGATAGTGGAGAGGATTC**

**TGAAGGAAGAATCCGATGAGGCACTTAAAATGACCATGGCCTCCGCACCTGCTTCGCGATACCTAACTGA**

**CATGACTATTGAGGAAATGTCAAGGGACTGGTTCATGCTAATGCCCAAGCAGAAAGTGGCAGGCCCTCTT**

**TGTATCAGAATGGACCAGGCAATCATGGATAAGAACATCATGTTGAAAGCGAATTTCAGTGTGATTTTTG**

**ACCGGCTAGAGACCCTAATATTACTAAGGGCTTTCACCGAAGAGGGAGCAATTGTTGGCGAAATTTCACC**

**ATTGCCTTCTCTTCCAGGACATACTAATGAGGATGTCAAAAATGCAATTGGGGTCCTCATCGGAGGACTT**

**GAATGGAATGATAACACAGTTCGAGTCTCTAAAACTCTACAGAGATTCGCTTGGAAAAGCAGTAATGAGA**

**ATGGGAGACCTCCACTCACTCCAAACCAGAAACGGAAAATGGCGAGAACAATTAGGTCAAAAGTTCGAAG**

**AAATAAGATGGCTGATTGAAGAAGTGAGGCACAAATTGAAGATAACAGAGAATAGTTTTGAGCAAATAAC**

**ATTTATGCAAGCCTTACAGCTACTATTTGAAGTGGAACAAGAGATAAGAACTTTCTCATTTCAGCTTATT**

**TAATGATAAA**

**>Influenza A virus (A/Aichi/2/1968(H3N2))**

**TCAATTATATTCAACATGGAAAGAATAAAAGAACTACGGAATCTGATGTCGCAGTCTCGCACTCGCGAGA**

**TACTAACAAAAACCACAGTTGACCATATGGCCATAATTAAGAAGTATACATCAGGGAGACAGGAAAAGAA**

**CCCGTCACTTAGGATGAAATGGATGATGGCAATGAAATATCCAATTACAGCTGACAAGAGGATAACAGAA**

**ATGGTTCCTGAGAGAAATGAGCAAGGACAAACTCTATGGAGCAAAATGAGTGATGCCGGATCAGATCGAG**

**TGATGGTATCACCCTTGGCAGTGACATGGTGGAATAGAAATGGACCAATGACAAGTACGGTTCATTATCC**

**AAAAGTCTACAAGACTTATTTTGAGAAAGTCGAAAGGTTAAAACATGGAACCTTTGGCCCTGTCCATTTT**

**AGAAACCAAGTCAAAATACGCCGAAGAGTTGACATAAACCCTGGTCATGCAGACCTCAGTGCCAAGGAGG**

**CACAAGATGTAATCATGGAAGTTGTTTTCCCCAATGAAGTGGGGGCCAGAATACTAACGTCGGAATCACA**

**ATTAACAATAACCAAAGAGAAAAAAGAAGAACTCCAAGATTGCAAAATTTCTCCTTTGATGGTTGCATAC**

**ATGTTAGAGAGAGAACTTGTCCGAAAAACGAGATTTCTCCCAGTTGCTGGTGGAACAAGCAGTGTATACA**

**TCGAAGTGTTACACTTGACTCAAGGAACGTGTTGGGAACAGATGTACACTCCAGGTGGAGAAGTGAGGAA**

**TGATGATGTTGATCAAAGTCTAATTATTGCAGCCAGGAACATAGTGAGAAGAGCAGCAGTATCAGCAGAT**

**CCACTAGCATCTTTATTGGAGATGTGCCACAGCACACAGATTGGCGGGACAAGGATGGTGGACATTCTTA**

**GGCAGAACCCAACGGAAGAACAAGCTGTGGATATATGCAAAGCTGCAATGGGACTGAGAATCAGCTCGTC**

**CTTCAGTTTTGGCGGATTCACATTTAAGAGAACAAGCGGGTCATCAATCAAGAGAGAGGAAGAATTGCTT**

**ACGGGCAATCTCCAAACATTAAAAATAAGGGTGCATGAGGGGCACGAGGAATTCACAATGGTGGGGAAAA**

**GGGCAACAGCTATACTCAGAAAAGCAACCAGGAGATTGGTTCAGCTGATAGTGAGTGGAAGAGACGAACA**

**GTCAGTAGCCGAAGCAATAATTGTAGCCATGGTGTTTTCACAAGAAGATTGCATGATAAAAGCAGTTAGA**

**GGTGATCTGAATTTCGTTAACAGGGCAAATCAGCGATTGAATCCCATGCATCAACTTTTAAGGCATTTTC**

**AGAAAGATGCGAAAGTGCTTTTTCAAAATTGGGGAATTGAACATATCGACAATGTAATGGGGATGATTGG**

**AGTATTACCAGACATGACTCCAAGCACAGAGATGTCAATGAGAGGGATAAGAGTCAGCAAAATGGGCGTG**

**GATGAATACTCCAGCACAGAGAGGGTTGTGGTGAGCATTGACCGGTTTTTGAGAGTTCGAGACCAACGAG**

**GAAATGTATTACTATCTCCTGAGGAGGTCAGTGAAACACAGGGGACAGAGAAACTGACAATAACTTACTC**

**ATCGTCAATGATGTGGGAGATTAATGGCCCTGAGTCAGTGTTGGTCAATACCTATCAGTGGATCATCAGA**

**AACTGGGAAACTGTCAAAATTCAATGGTCTCAGAATCCTACAATGTTATACAACAAAATGGAATTTGAGC**

**CATTTCAGTCTTTAGTTCCTAAGGCCATTAGAGGCCAATACAGTGGATTTGTTAGGACTCTATTCCAACA**

**AATGAGGGATGTACTTGGGACATTTGATACCACCCAGATAATAAAGCTTCTCCCCTTTGCAGCCGCCCCA**

**CCAAAGCAAAGTAGGATGCAGTTCTCTTCATTGACTGTGAATGTGAGGGGATCAGGGATGAGAATACTTG**

**TAAGGGGCAATTCTCCTGTATTCAACTACAACAAGACAACGAAAAGACTAACAATTCTCGGAAAAGATGC**

**TGGCACTTTAATTGAAGACCCAGATGAAGGTACATCCGGAGTGGAGTCAGCTGTTCTGAGAGGGTTCCTC**

**ATTCTGGGTAAGGAAGATAGAAGATATGGACCAGCATTAAGCATCAATGAACTGAGTAACCTTGCAAAAG**

**GAGAAAAGGCTAATGTACTAATTGGGCAAGGAGACGTGGTGTTGGTAATGAAACGAAAACGGGACTCTAG**

**CATACTTACTGACAGCCAGACAGCGACCAAAAGAATTCGGATGGCCATCAATTAATGTTGAATAGTTTAA**

**AAACGACAAACCATTTGAATGGATGTCAATCCGACTTTACTTTTCTTGAAAGTTCCAGCGCAAAATGCCATAAGCA**

**CCACATTCCCTTATACTGGAGATCCTCCATACAGCCATGGAACAGGAACAGGATACACCATGGACACAGT**

**CAACAGAACACATCAATATTCAGAAAAAGGGAAGTGGACAACAAACACGGAAACTGGAGCGCCCCAACTT**

**AACCCAATTGATGGACCACTACCTGAGGATAATGAGCCAAGTGGATATGCACAAACAGACTGTGTCCTGG**

**AAGCAATGGCTTTCCTTGAAGAATCCCACCCAGGGATCTTTGAAAACTCGTGTCTTGAAACGATGGAAGT**

**TGTTCAACAAACAAGGGTGGACAGACTGACCCAAGGTCGTCAGACCTATGATTGGACATTAAACAGAAAT**

**CAACCGGCCGCAACTGCATTAGCCAACACTATAGAAGTCTTCAGATCGAATGGTCTAACAGCTAATGAGT**

**CGGGAAGGCTAATAGATTTCCTCAAAGATGTGATGGAATCAATGGATAAAGAGGAAATGGAGATAACAAC**

**ACACTTCCAAAGAAAAAGAAGAGTAAGAGACAACATGACCAAGAAAATGGTCACACAAAGAACAATAGGA**

**AAGAAGAAGCAGAGAGTGAACAAGAGAAGCTATCTAATAAGAGCATTAACATTGAACACAATGACCAAAG**

**ATGCAGAAAGAGGTAAATTAAAGAGAAGAGCTATTGCAACACCCGGGATGCAAATCAGAGGGTTCGTGTA**

**CTTTGTTGAAACTCTAGCTAGGAGCATTTGTGAGAAGCTTGAACAGTCTGGACTTCCAGTTGGAGGTAAT**

**GAAAAGAAGGCCAAACTGGCAAATGTTGTGAGAAAGATGATGACTAATTCACAAGACACAGAGCTTTCTT**

**TCACAATTACTGGAGACAATACTAAATGGAATGAAAATCAAAATCCTCGAATGTTCCTGGCGATGATTAC**

**ATATATCACAAAAAATCAACCTGAATGGTTCAGAAACGTTCTGAGCATCGCACCCATAATGTTCTCAAAC**

**AAAATGGCGAGACTAGGGAAAGGATACATGTTCGAAAGTAAGAGCATGAAGCTCCGAACACAAATACCAG**

**CAGAAATGCTAGCAAGCATTGACCTAAAGTATTTCAATGAATCAACAAGAAAGAAAATTGAGAAAATAAG**

**GCCTCTTCTAATAGATGGCACAGCTTCATTGAGTCCTGGAATGATGATGGGCATGTTCAACATGCTAAGT**

**ACGGTTTTAGGAGTCTCAATCCTGAATCTTGGGCAAAAGAGATACACCAAAACAACATACTGGTGGGATG**

**GACTCCAATCCTCCGATGATTTTGCTCTCATAGTGAATGCACCAAATCATGAGGGAATACAAGCAGGAGT**

**GGATAGATTCTACAGAACCTGCAAGTTAGTCGGAATCAATATGAGCAAGAAGAAGTCCTATATAAATAGG**

**ACAGGAACATTTGAATTCACAAGCTTTTTCTATCGCTATGGATTTGTAGCCAATTTTAGCATGGAGCTGC**

**CCAGTTTTGGAGTGTCTGGGATTAATGAGTCAGCTGATATGAGCATTGGAGTAACAGTGATAAAGAACAA**

**CATGATAAACAATGACCTTGGACCAGCAACAGCCCAGATGGCTCTTCAACTGTTCATCAAGGACTACAGA**

**TATACATACCGGTGCCACAGAGGAGACACACAAATTCAGACGAGGAGATCATTCGAGCTAAAGAAGCTGT**

**GGGAGCAAACCCGCTCAAAGGCAGGACTATTGGTTTCAGATGGAGGACCAAACTTATACAATATCCGGAA**

**TCTTCACATCCCGGAAGTCTGCTTAAAGTGGGAGCTAATGGATGAGGACTATCAGGGAAGACTTTGTAAT**

**CCCCTGAATCCATTTGTCAGCCATAAGGAGATTGAGTCTGTAAACAATGCTGTGGTAATGCCAGCTCATG**

**GTCCAGCCAAGAGCATGGAATATGACGCTGTTGCAACTACACACTCCTGGATTCCTAAGAGGAACCGCTC**

**TATCCTCAACACAAGCCAAAGGGGAATTCTTGAGGATGAACAGATGTATCAGAAGTGCTGCAACCTGTTC**

**GAGAAATTTTTCCCCAGTAGTTCATACAGGAGACCGGTTGGAATTTCCAGCATGGTGGAGGCCATGGTGT**

**CTAGGGCCCGGATTGATGCCAGAATTGACTTCGAGTCTGGACGGATTAATAAAGAAGAGTTCGCCGAGAT**

**CATGAAGATCTGTTCCACCATTGAAGAGCTCAGACGGCAAAAATAGTGAATTTAGCTTGTCCTTCATGAA**

**AAAATGTACTGATTCGAAATGGAAGATTTTGTACGACAATGCTTTAATCCGATGATTGTCGAACTTGCGGAAAAGG**

**CAATGAAAGAGTATGGAGAGGATCTTAAAATCGAAACAAACAAATTTGCAGCAATATGCACTCACTTGGA**

**AGTATGCTTCATGTATTCAGATTTTCATTTCATCAATGAGCAAGGCGAGTCAATAGTGGTAGAACTTGAT**

**GATCCAAATGCACTTTTGAAGCACAGATTTGAAATAATAGAGGGAAGAGACCGCACAATGGCCTGGACAG**

**TAGTAAACAGTATTTGCAACACCACAGGAGCTGAGAAACCGAAGTTTCTGCCAGATTTGTATGATTACAA**

**GGAGAATAGATTCATCGAGATTGGAGTGACAAGGAGAGAAGTCCACATATACTACCTTGAAAAGGCCAAT**

**AAAATTAAATCTGAGAATACACACATCCACATTTTCTCATTCACTGGGGAAGAAATGGCCACAAAGGCCG**

**ACTACACTCTCGATGAGGAAAGCAGGGCTAGGATCAAAACCAGACTATTCACCATAAGACAAGAGATGGC**

**CAACAGAGGCCTCTGGGATTCCTTTCGTCAGTCCGAAAGAGGCGAAGAAACAATTGAAGAAAGATTTGAA**

**ATCACAGGGACAATGCGCAGGCTTGCCGACCAAAGTCTCCCGCCGAACTTCTCCTGCCTTGAGAATTTTA**

**GAGCCTATGTGGATGGATTCGAACCGAACGGCTACATTGAGGGCAAGCTTTCTCAAATGTCCAAAGAAGT**

**GAATGCAAAAATTGAACCTTTTCTGAAAACAACACCAAGACCAATTAGACTTCCGGATGGGCCTCCTTGT**

**TTTCAGCGGTCCAAATTCCTTCTGATGGATGCTTTAAAGTTAAGCATTGAGGATCCAAGTCACGAGGGGG**

**AGGGAATACCACTATATGATGCGATCAAATGCATGAGAACATTTTTTGGATGGAAAGAACCCTATATTGT**

**TAAACCACACGAAAAGGGGATAAATCCAAATTATCTGCTGTCATGGAAGCAAGTACTGGCAGAACTGCAG**

**GACATTGAAAATGAGGAGAAAATTCCAAGAACTAAAAACATGAAGAAAACGAGTCAGCTAAAGTGGGCAC**

**TTGGTGAGAACATGGCACCAGAGAAGGTAGACTTTGACAACTGTAGAGACGTAAGCGATTTGAAGCAATA**

**TGATAGTGACGAACCTGAATTAAGGTCACTTTCAAGCTGGATCCAGAATGAGTTCAACAAGGCATGCGAG**

**CTGACCGATTCAACTTGGATAGAGCTCGATGAGATTGGAGAAGACGTGGCTCCAATTGAATACATTGCAA**

**GCATGAGAAGGAATTACTTCACAGCAGAGGTGTCCCATTGCAGAGCCACAGAATATATAATGAAGGGGGT**

**ATACATTAATACTGCCTTGCTTAATGCATCCTGTGCAGCAATGGACGATTTCCAACTAATTCCCATGATA**

**AGCAAGTGTAGAACTAAAGAGGGAAGGCGAAAGACCAATTTATATGGCTTCATCATAAAAGGAAGATCTC**

**ACTTAAGGAATGACACCGACGTGGTAAACTTTGTGAGCATGGAGTTTTCTCTCACTGACCCGAGACTTGA**

**GCCACACAAATGGGAGAAATACTGTGTCCTTGAGATAGGAGATATGCTACTAAGAAGTGCTATAGGCCAG**

**ATGTCAAGGCCTATGTTCTTGTATGTGAGAACAAATGGAACATCAAAGATTAAAATGAAATGGGGAATGG**

**AGATGAGGCGTTGCCTCCTTCAGTCACTCCAACAAATCGAGAGTATGATTGAAGCAGAGTCATCTGTCAA**

**AGAAAAAGACATGACCAAAGAGTTTTTTGAGAATAAATCAGAAACATGGCCCATTGGGGAGTCCCCCAAG**

**GGAGTGGAAGATGGTTCCATTGGGAAGGTCTGCAGGACTTTATTGGCCAAGTCGGTATTCAATAGCCTGT**

**ATGCATCCCCGCAATTGGAAGGGTTTTCAGCTGAGTCAAGAAAACTGCTTCTTGTCGTTCAGGCTCTTAG**

**GGACAATCTTGAACCTGGAACCTTTGATCTTGAGGGGCTATATGAAGCAATTGAGGAGTGCCTGATTAAT**

**GATCCCTGGGTTTTGCTTAATGCGTCGTGGTTCAACTCCTTCCTAACACATGCATTAAGATAGTTGTGGC**

**AATGCTACTATTTGCTATCCATACTGTCCAAAAAAGTAGGATAATTCTATTAATCATGAAGACCATCATT**

**GCTTTGAGCTACATTTTCTGTCTGGCTCTCGGCCAAGA**

**CCTTCCAGGAAATGACAACAGCACAGCAACGCTGTGCCTGGGACATCATGCGGTGCCAAACGGAACACTA**

**GTGAAAACAATCACAGATGATCAGATTGAAGTGACTAATGCTACTGAGCTAGTTCAGAGCTCCTCAACGG**

**GGAAAATATGCAACAATCCTCATCGAATCCTTGATGGAATAGACTGCACACTGATAGATGCTCTATTGGG**

**GGACCCTCATTGTGATGTTTTTCAAAATGAGACATGGGACCTTTTCGTTGAACGCAGCAAAGCTTTCAGC**

**AACTGTTACCCTTATGATGTGCCAGATTATGCCTCCCTTAGGTCACTAGTTGCCTCGTCAGGCACTCTGG**

**AGTTTATCACTGAGGGTTTCACTTGGACTGGGGTCACTCAGAATGGGGGAAGCAATGCTTGCAAAAGGGG**

**ACCTGGTAGCGGTTTTTTCAGTAGACTGAACTGGTTGACCAAATCAGGAAGCACATATCCAGTGCTGAAC**

**GTGACTATGCCAAACAATGACAATTTTGACAAACTATACATTTGGGGGGTTCACCACCCGAGCACGAACC**

**AAGAACAAACCAGCCTGTATGTTCAAGCATCAGGGAGAGTCACAGTCTCTACCAGGAGAAGCCAGCAAAC**

**TATAATCCCGAATATCGGGTCCAGACCCTGGGTAAGGGGTCTGTCTAGTAGAATAAGCATCTATTGGACA**

**ATAGTTAAGCCGGGAGACGTACTGGTAATTAATAGTAATGGGAACCTAATCGCTCCTCGGGGTTATTTCA**

**AAATGCGCACTGGGAAAAGCTCAATAATGAGGTCAGATGCACCTATTGATACCTGTATTTCTGAATGCAT**

**CACTCCAAATGGAAGCATTCCCAATGACAAGCCCTTTCAAAACGTAAACAAGATCACATATGGAGCATGC**

**CCCAAGTATGTTAAGCAAAACACCCTGAAGTTGGCAACAGGGATGCGGAATGTACCAGAGAAACAAACTA**

**GAGGCCTATTCGGCGCAATAGCAGGTTTCATAGAAAATGGTTGGGAGGGAATGATAGACGGTTGGTACGG**

**TTTCAGGCATCAAAATTCTGAGGGCACAGGACAAGCAGCAGATCTTAAAAGCACTCAAGCAGCCATCGAC**

**CAAATCAATGGGAAATTGAACAGGGTAATCGAGAAGACGAACGAGAAATTCCATCAAATCGAAAAGGAAT**

**TCTCAGAAGTAGAAGGGAGAATTCAGGACCTCGAGAAATACGTTGAAGACACTAAAATAGATCTCTGGTC**

**TTACAATGCGGAGCTTCTTGTCGCTCTGGAGAATCAACATACAATTGACCTGACTGACTCGGAAATGAAC**

**AAGCTGTTTGAAAAAACAAGGAGGCAACTGAGGGAAAATGCTGAAGACATGGGCAATGGTTGCTTCAAAA**

**TATACCACAAATGTGACAACGCTTGCATAGAGTCAATCAGAAATGGGACTTATGACCATGATGTATACAG**

**AGACGAAGCATTAAACAACCGGTTTCAGATCAAAGGTGTTGAACTGAAGTCTGGATACAAAGACTGGATC**

**CTGTGGATTTCCTTTGCCATATCATGCTTTTTGCTTTGTGTTGTTTTGCTGGGGTTCATCATGTGGGCCT**

**GCCAGAGAGGCAACATTAGGTGCAACATTTGCATTTGAGTGTATTAGTAATTAAAAACACGTAGATAATC**

**ACTCACTGAGTGACATCAAAATCATGGCGTCCCAAGGCACCAAACGGTCTTATGAACAGA**

**TGGAAACTGATGGGGAACGCCAGAATGCAACTGAGATCAGAGCATCCGTCGGGAAGATGATTGATGGAAT**

**TGGACGATTCTACATCCAAATGTGCACTGAACTTAAACTCAGTGATTATGAGGGGCGACTGATCCAGAAC**

**AGCTTAACAATAGAGAGAATGGTGCTCTCTGCTTTTGACGAAAGAAGGAATAAATATCTGGAAGAACATC**

**CCAGCGCGGGGAAGGATCCTAAGAAAACTGGAGGACCCATATACAAGAGAGTAGATAGAAAGTGGATGAG**

**GGAACTCGTCCTTTATGACAAAGAAGAAATAAGGCGAATCTGGCGCCAAGCCAATAATGGTGATGATGCA**

**ACAGCTGGTCTGACTCACATGATGATCTGGCATTCCAATTTGAATGATACAACATACCAGAGGACAAGAG**

**CTCTTGTTCGCACCGGCATGGATCCCAGGATGTGCTCTCTGATGCAGGGTTCGACTCTCCCTAGGAGGTC**

**TGGAGCTGCAGGCGCTGCAGTCAAAGGAGTTGGGACAATGGTGATGGAGTTGATAAGGATGATCAAACGT**

**GGGATCAATGATCGGAACTTCTGGAGAGGTGAAAATGGACGAAAAACAAGGAGTGCTTACGAGAGAATGT**

**GCAACATTCTCAAAGGAAAATTTCAAACAGCTGCACAAAGGGCAATGATGGATCAAGTGAGAGAAAGTCG**

**GAACCCAGGAAATGCTGAGATCGAAGATCTCATCTTTCTGGCACGGTCTGCACTCATATTGAGAGGGTCA**

**GTTGCTCACAAATCTTGTCTGCCCGCCTGTGTGTATGGACCTGCCGTAGCCAGTGGCTACGACTTCGAAA**

**AAGAGGGATACTCTTTAGTGGGAATAGACCCTTTCAAACTGCTTCAAAACAGCCAAGTATACAGCCTAAT**

**CAGACCGAACGAGAATCCAGCACACAAGAGTCAGCTGGTGTGGATGGCATGCAATTCTGCTGCATTTGAA**

**GATCTAAGAGTATTAAGCTTCATCAGAGGGACCAAAGTATCCCCAAGGGGGAAACTTTCCACTAGAGGAG**

**TACAAATTGCTTCAAATGAAAACATGGATGCTATGGAATCAAGTACTCTTGAACTGAGAAGCAGGTACTG**

**GGCCATAAGAACCAGAAGTGGAGGAAACACTAATCAACAGAGGGCCTCTGCAGGTCAAATCAGTGTGCAA**

**CCTGCATTTTCTGTGCAAAGAAACCTCCCATTTGACAAACCAACCATCATGGCAGCATTCACTGGGAATA**

**CAGAGGGAAGAACATCAGACATGAGGGCAGAAATTATAAGGATGATGGAAGGTGCAAAACCAGAAGAAAT**

**GTCCTTCCAGGGGCGGGGAGTCTTCGAGCTCTCGGACGAAAGGGCAGCGAACCCGATCGTGCCCTCTTTT**

**GACATGAGTAATGAAGGATCTTATTTCTTCGGAGACAATGCAGAGGAGTACGACAATTAAGGAAAAATAC**

**AGTGAAAATGAATCCAAATCAAAAGATAATAACAATTGGCTCTGTCTCTCTCACCATTGCAACAGTATGC**

**TTCCTCATGCAGATTGCCATCCTGGTAACTACTGTAACATTGCATTTTAAGCAATATGAGTGCGACTCCC**

**CCGCGAGCAACCAAGTAATGCCGTGTGAACCAATAATAATAGAAAGGAACATAACAGAGATAGTGTATTT**

**GAATAACACCACCATAGAGAAAGAGATATGCCCCAAAGTAGTGGAATACAGAAATTGGTCAAAGCCGCAA**

**TGTCAAATTACAGGATTTGCACCTTTTTCTAAGGACAATTCAATCCGGCTTTCTGCTGGTGGGGACATTT**

**GGGTGACGAGAGAACCTTATGTGTCATGCGATCATGGCAAGTGTTATCAATTTGCACTCGGGCAGGGGAC**

**CACACTAGACAACAAACATTCAAATGACACAATACATGATAGAATCCCTCATCGAACCCTATTAATGAAT**

**GAGTTGGGTGTTCCATTTCATTTAGGAACCAGGCAAGTGTGTATAGCATGGTCCAGCTCAAGTTGTCACG**

**ATGGAAAAGCATGGCTGCATGTTTGTATCACTGGGGATGACAAAAATGCAACTGCTAGCTTCATTTATGA**

**CGGGAGGCTTGTGGACAGTATTGGTTCATGGTCTCAAAATATCCTCAGAACCCAGGAGTCGGAATGCGTT**

**TGTATCAATGGGACTTGCACAGTAGTAATGACTGATGGAAGTGCTTCAGGAAGAGCCGATACTAGAATAC**

**TATTCATTGAAGAGGGGAAAATTGTCCATATTAGCCCATTGTCAGGAAGTGCTCAGCATGTAGAAGAGTG**

**TTCCTGTTATCCTAGATATCCTGGCGTCAGATGTATCTGCAGAGACAACTGGAAAGGCTCTAATAGGCCC**

**GTCGTAGACATAAATATGGAAGATTATAGCATTGATTCCAGTTATGTGTGCTCAGGGCTTGTTGGCGACA**

**CACCTAGAAACGACGACAGATCTAGCAATAGCAATTGCAGGAATCCTAATAATGAGAGAGGGAATCAAGG**

**AGTGAAAGGCTGGGCCTTTGACAATGGAGATGACGTGTGGATGGGAAGAACGATCAGCAAGGATTTACGC**

**TCAGGTTATGAAACTTTCAAAGTCATTGGTGGTTGGTCCACACCTAATTCCAAATCGCAGATCAATAGAC**

**AAGTCATAGTTGACAGCGATAATCGGTCAGGTTACTCTGGTATTTTCTCTGTTGAGGGCAAAAGCTGCAT**

**CAATAGGTGCTTTTATGTGGAGTTGATAAGGGGAAGGAAACAGGAGACTAGAGTGTGGTGGACCTCAAAC**

**AGTATTGTTGTGTTTTGTGGCACTTCAGGTACCTATGGAACAGGCTCATGGCCTGATGGGGCGAACATCA**

**ATTTCATGCCTATATAAGCTTTCGCAATTTTAGAAATAGATATTGAAAGATGAGCCTTCTAACCGAGGTCGAAACGTACGTTCTCTCTATCGTCCCGTCAGGCCCC**

**CTCAAAGCCGAGATCGCACAGAGACTTGAAGATGTCTTTGCTGGGAAGAACACAGATCTTGAGGCTCTCA**

**TGGAATGGCTAAAGACAAGACCAATCCTGTCACCTCTGACTAAGGGGATTTTGGGATTTGTATTCACGCT**

**CACCGTGCCCAGTGAGCGAGGACTGCAGCGTAGACGCTTTGTCCAAAATGCCCTCAATGGGAATGGGGAT**

**CCAAATAACATGGACAGAGCAGTTAAACTGTATAGAAAACTTAAGAGGGAGATAACATTCCATGGGGCCA**

**AAGAAATAGCACTCAGTTATTCTGCTGGTGCACTTGCCAGTTGCATGGGCCTCATATACAACAGGATGGG**

**GGCTGTGACCACTGAAGTGGCCTTTGGCCTGGTATGTGCAACCTGTGAACAGATTGCTGACTCCCAGCAT**

**AGGTCTCATAGGCAAATGGTGACAACAACCAATCCACTAATAAGACATGAGAACAGAATGGTTCTGGCCA**

**GCACTACAGCTAAGGCTATGGAGCAAATGGCTGGATCGAGTGAGCAGGCAGCCGAGGCCATGGAGGTTGC**

**TAGTCAGGCCAGGCAAATGGTGCAGGCAATGAGAGCCATTGGGACTCATCCTAGATCCAGTGCTGGTCTA**

**AAAGATGATCTTCTTGAAAATTTGCAGGCCTATCAGAAACGAATGGGGGTGCAGATGCAACGATTCAAGT**

**GACCCTCTTGTTGTTGCTGCGAGTATCATTGGGATCTTGCACTTGATATTGTGGATTCTTGATCGTCTTT**

**TTTTCAAATGCATTTATCGATTCTTTGAACACGGTCTGAAAAGAGGGCCTTCTACGGAAGGAGTACCTGA**

**GTCTATGAGGGAAGAATATCGAAAGGAACAGCAGAGTGCTGTGGATGCTGACGATAGTCATTTTGTCAGC**

**ATAGAGCTGGAGTAAAAAACTAGTGACAAAGACATAATGGATTCTAACACTGTGTCAAGTTTTCAGGTAG**

**ATTGCTTCCTTTGGCATGTCCG**

**AAAACAAGTTGTAGACCAAGAACTAGGTGATGCCCCATTCCTTGATCGGCTTCGCCGAGATCAGAAGTCC**

**CTAAGGGGAAGAGGCAGCACTCTCGGTCTAAACATCGAAGCAGCCACCCGTGTTGGAAAGCAGATAGTAG**

**AGAGGATTCTGAAGGAAGAATCCGATGAGGCACTTAAAATGACCATGGCCTCCGCACCTGCTTCGCGATA**

**CCTAACTGACATGACTATTGAGGAATTGTCAAGGGACTGGTTCATGCTAATGCCCAAGCAGAAAGTGGAA**

**GGACCTCTTTGCATCAGAATAGACCAGGCAATCATGGATAAGAACGTCATGTTGAAAGCGAATTTCAGTG**

**TGATTTTTGACCGGCTAGAGACCCTAATATTACTAAGGGCTTTCACCGAAGAGGGAGCAATTGTTGGCGA**

**AATCTCACCATTGCCTTCTCTTCCAGGACATACTATTGAGGATGTCAAAAATGCAATTGGGGTCCTCATC**

**GGAGGACTTGAATGGAATGATAACACAGTTCGAGTCTCTAAAACTCTACAGAGATTCGCTTGGGGAAGCA**

**GTAATGAGAATGGGAGACCTCCACTCACTCCAAAACAGAAACGGAAAATGGCGAGAACAGTTAGGTCAAA**

**AGTTCGAAGAGATAAGATGGCTGATTGAAGAAGTGAGACACAGATTGAAGACAACAGAGAATAGTTTTGA**

**GCAAATAACATTTATGCAAGCCTTACAGCTACTATTTGAAGTGGAACAGGAGATAAGAACTTTCTCGTTT**

**CAGCTTATTTAATGATAAAAAACAC**

**SARS-CoV-1**

**ATATTAGGTTTTTACCTACCCAGGAAAAGCCAACCAACCTCGATCTCTTGTAGATCTGTT**

**CTCTAAACGAACTTTAAAATCTGTGTAGCTGTCGCTCGGCTGCATGCCTAGTGCACCTAC**

**GCAGTATAAACAATAATAAATTTTACTGTCGTTGACAAGAAACGAGTAACTCGTCCCTCT**

**TCTGCAGACTGCTTACGGTTTCGTCCGTGTTGCAGTCGATCATCAGCATACCTAGGTTTC**

**GTCCGGGTGTGACCGAAAGGTAAGATGGAGAGCCTTGTTCTTGGTGTCAACGAGAAAACA**

**CACGTCCAACTCAGTTTGCCTGTCCTTCAGGTTAGAGACGTGCTAGTGCGTGGCTTCGGG**

**GACTCTGTGGAAGAGGCCCTATCGGAGGCACGTGAACACCTCAAAAATGGCACTTGTGGT**

**CTAGTAGAGCTGGAAAAAGGCGTACTGCCCCAGCTTGAACAGCCCTATGTGTTCATTAAA**

**CGTTCTGATGCCTTAAGCACCAATCACGGCCACAAGGTCGTTGAGCTGGTTGCAGAAATG**

**GACGGCATTCAGTACGGTCGTAGCGGTATAACACTGGGAGTACTCGTGCCACATGTGGGC**

**GAAACCCCAATTGCATACCGCAATGTTCTTCTTCGTAAGAACGGTAATAAGGGAGCCGGT**

**GGTCATAGCTATGGCATCGATCTAAAGTCTTATGACTTAGGTGACGAGCTTGGCACTGAT**

**CCCATTGAAGATTATGAACAAAACTGGAACACTAAGCATGGCAGTGGTGCACTCCGTGAA**

**CTCACTCGTGAGCTCAATGGAGGTGCAGTCACTCGCTATGTCGACAACAATTTCTGTGGC**

**CCAGATGGGTACCCTCTTGATTGCATCAAAGATTTTCTCGCACGCGCGGGCAAGTCAATG**

**TGCACTCTTTCCGAACAACTTGATTACATCGAGTCGAAGAGAGGTGTCTACTGCTGCCGT**

**GACCATGAGCATGAAATTGCCTGGTTCACTGAGCGCTCTGATAAGAGCTACGAGCACCAG**

**ACACCCTTCGAAATTAAGAGTGCCAAGAAATTTGACACTTTCAAAGGGGAATGCCCAAAG**

**TTTGTGTTTCCTCTTAACTCAAAAGTCAAAGTCATTCAACCACGTGTTGAAAAGAAAAAG**

**ACTGAGGGTTTCATGGGGCGTATACGCTCTGTGTACCCTGTTGCATCTCCACAGGAGTGT**

**AACAATATGCACTTGTCTACCTTGATGAAATGTAATCATTGCGATGAAGTTTCATGGCAG**

**ACGTGCGACTTTCTGAAAGCCACTTGTGAACATTGTGGCACTGAAAATTTAGTTATTGAA**

**GGACCTACTACATGTGGGTACCTACCTACTAATGCTGTAGTGAAAATGCCATGTCCTGCC**

**TGTCAAGACCCAGAGATTGGACCTGAGCATAGTGTTGCAGATTATCACAACCACTCAAAC**

**ATTGAAACTCGACTCCGCAAGGGAGGTAGGACTAGATGTTTTGGAGGCTGTGTGTTTGCC**

**TATGTTGGCTGCTATAATAAGCGTGCCTACTGGGTTCCTCGTGCTAGTGCTGATATTGGC**

**TCAGGCCATACTGGCATTACTGGTGACAATGTGGAGACCTTGAATGAGGATCTCCTTGAG**

**ATACTGAGTCGTGAACGTGTTAACATTAACATTGTTGGCGATTTTCATTTGAATGAAGAG**

**GTTGCCATCATTTTGGCATCTTTCTCTGCTTCTACAAGTGCCTTTATTGACACTATAAAG**

**AGTCTTGATTACAAGTCTTTCAAAACCATTGTTGAGTCCTGCGGTAACTATAAAGTTACC**

**AAGGGAAAGCCCGTAAAAGGTGCTTGGAACATTGGACAACAGAGATCAGTTTTAACACCA**

**CTGTGTGGTTTTCCCTCACAGGCTGCTGGTGTTATCAGATCAATTTTTGCGCGCACACTT**

**GATGCAGCAAACCACTCAATTCCTGATTTGCAAAGAGCAGCTGTCACCATACTTGATGGT**

**ATTTCTGAACAGTCATTACGTCTTGTCGACGCCATGGTTTATACTTCAGACCTGCTCACC**

**AACAGTGTCATTATTATGGCATATGTAACTGGTGGTCTTGTACAACAGACTTCTCAGTGG**

**TTGTCTAATCTTTTGGGCACTACTGTTGAAAAACTCAGGCCTATCTTTGAATGGATTGAG**

**GCGAAACTTAGTGCAGGAGTTGAATTTCTCAAGGATGCTTGGGAGATTCTCAAATTTCTC**

**ATTACAGGTGTTTTTGACATCGTCAAGGGTCAAATACAGGTTGCTTCAGATAACATCAAG**

**GATTGTGTAAAATGCTTCATTGATGTTGTTAACAAGGCACTCGAAATGTGCATTGATCAA**

**GTCACTATCGCTGGCGCAAAGTTGCGATCACTCAACTTAGGTGAAGTCTTCATCGCTCAA**

**AGCAAGGGACTTTACCGTCAGTGTATACGTGGCAAGGAGCAGCTGCAACTACTCATGCCT**

**CTTAAGGCACCAAAAGAAGTAACCTTTCTTGAAGGTGATTCACATGACACAGTACTTACC**

**TCTGAGGAGGTTGTTCTCAAGAACGGTGAACTCGAAGCACTCGAGACGCCCGTTGATAGC**

**TTCACAAATGGAGCTATCGTTGGCACACCAGTCTGTGTAAATGGCCTCATGCTCTTAGAG**

**ATTAAGGACAAAGAACAATACTGCGCATTGTCTCCTGGTTTACTGGCTACAAACAATGTC**

**TTTCGCTTAAAAGGGGGTGCACCAATTAAAGGTGTAACCTTTGGAGAAGATACTGTTTGG**

**GAAGTTCAAGGTTACAAGAATGTGAGAATCACATTTGAGCTTGATGAACGTGTTGACAAA**

**GTGCTTAATGAAAAGTGCTCTGTCTACACTGTTGAATCCGGTACCGAAGTTACTGAGTTT**

**GCATGTGTTGTAGCAGAGGCTGTTGTGAAGACTTTACAACCAGTTTCTGATCTCCTTACC**

**AACATGGGTATTGATCTTGATGAGTGGAGTGTAGCTACATTCTACTTATTTGATGATGCT**

**GGTGAAGAAAACTTTTCATCACGTATGTATTGTTCCTTTTACCCTCCAGATGAGGAAGAA**

**GAGGACGATGCAGAGTGTGAGGAAGAAGAAATTGATGAAACCTGTGAACATGAGTACGGT**

**ACAGAGGATGATTATCAAGGTCTCCCTCTGGAATTTGGTGCCTCAGCTGAAACAGTTCGA**

**GTTGAGGAAGAAGAAGAGGAAGACTGGCTGGATGATACTACTGAGCAATCAGAGATTGAG**

**CCAGAACCAGAACCTACACCTGAAGAACCAGTTAATCAGTTTACTGGTTATTTAAAACTT**

**ACTGACAATGTTGCCATTAAATGTGTTGACATCGTTAAGGAGGCACAAAGTGCTAATCCT**

**ATGGTGATTGTAAATGCTGCTAACATACACCTGAAACATGGTGGTGGTGTAGCAGGTGCA**

**CTCAACAAGGCAACCAATGGTGCCATGCAAAAGGAGAGTGATGATTACATTAAGCTAAAT**

**GGCCCTCTTACAGTAGGAGGGTCTTGTTTGCTTTCTGGACATAATCTTGCTAAGAAGTGT**

**CTGCATGTTGTTGGACCTAACCTAAATGCAGGTGAGGACATCCAGCTTCTTAAGGCAGCA**

**TATGAAAATTTCAATTCACAGGACATCTTACTTGCACCATTGTTGTCAGCAGGCATATTT**

**GGTGCTAAACCACTTCAGTCTTTACAAGTGTGCGTGCAGACGGTTCGTACACAGGTTTAT**

**ATTGCAGTCAATGACAAAGCTCTTTATGAGCAGGTTGTCATGGATTATCTTGATAACCTG**

**AAGCCTAGAGTGGAAGCACCTAAACAAGAGGAGCCACCAAACACAGAAGATTCCAAAACT**

**GAGGAGAAATCTGTCGTACAGAAGCCTGTCGATGTGAAGCCAAAAATTAAGGCCTGCATT**

**GATGAGGTTACCACAACACTGGAAGAAACTAAGTTTCTTACCAATAAGTTACTCTTGTTT**

**GCTGATATCAATGGTAAGCTTTACCATGATTCTCAGAACATGCTTAGAGGTGAAGATATG**

**TCTTTCCTTGAGAAGGATGCACCTTACATGGTAGGTGATGTTATCACTAGTGGTGATATC**

**ACTTGTGTTGTAATACCCTCCAAAAAGGCTGGTGGCACTACTGAGATGCTCTCAAGAGCT**

**TTGAAGAAAGTGCCAGTTGATGAGTATATAACCACGTACCCTGGACAAGGATGTGCTGGT**

**TATACACTTGAGGAAGCTAAGACTGCTCTTAAGAAATGCAAATCTGCATTTTATGTACTA**

**CCTTCAGAAGCACCTAATGCTAAGGAAGAGATTCTAGGAACTGTATCCTGGAATTTGAGA**

**GAAATGCTTGCTCATGCTGAAGAGACAAGAAAATTAATGCCTATATGCATGGATGTTAGA**

**GCCATAATGGCAACCATCCAACGTAAGTATAAAGGAATTAAAATTCAAGAGGGCATCGTT**

**GACTATGGTGTCCGATTCTTCTTTTATACTAGTAAAGAGCCTGTAGCTTCTATTATTACG**

**AAGCTGAACTCTCTAAATGAGCCGCTTGTCACAATGCCAATTGGTTATGTGACACATGGT**

**TTTAATCTTGAAGAGGCTGCGCGCTGTATGCGTTCTCTTAAAGCTCCTGCCGTAGTGTCA**

**GTATCATCACCAGATGCTGTTACTACATATAATGGATACCTCACTTCGTCATCAAAGACA**

**TCTGAGGAGCACTTTGTAGAAACAGTTTCTTTGGCTGGCTCTTACAGAGATTGGTCCTAT**

**TCAGGACAGCGTACAGAGTTAGGTGTTGAATTTCTTAAGCGTGGTGACAAAATTGTGTAC**

**CACACTCTGGAGAGCCCCGTCGAGTTTCATCTTGACGGTGAGGTTCTTTCACTTGACAAA**

**CTAAAGAGTCTCTTATCCCTGCGGGAGGTTAAGACTATAAAAGTGTTCACAACTGTGGAC**

**AACACTAATCTCCACACACAGCTTGTGGATATGTCTATGACATATGGACAGCAGTTTGGT**

**CCAACATACTTGGATGGTGCTGATGTTACAAAAATTAAACCTCATGTAAATCATGAGGGT**

**AAGACTTTCTTTGTACTACCTAGTGATGACACACTACGTAGTGAAGCTTTCGAGTACTAC**

**CATACTCTTGATGAGAGTTTTCTTGGTAGGTACATGTCTGCTTTAAACCACACAAAGAAA**

**TGGAAATTTCCTCAAGTTGGTGGTTTAACTTCAATTAAATGGGCTGATAACAATTGTTAT**

**TTGTCTAGTGTTTTATTAGCACTTCAACAGCTTGAAGTCAAATTCAATGCACCAGCACTT**

**CAAGAGGCTTATTATAGAGCCCGTGCTGGTGATGCTGCTAACTTTTGTGCACTCATACTC**

**GCTTACAGTAATAAAACTGTTGGCGAGCTTGGTGATGTCAGAGAAACTATGACCCATCTT**

**CTACAGCATGCTAATTTGGAATCTGCAAAGCGAGTTCTTAATGTGGTGTGTAAACATTGT**

**GGTCAGAAAACTACTACCTTAACGGGTGTAGAAGCTGTGATGTATATGGGTACTCTATCT**

**TATGATAATCTTAAGACAGGTGTTTCCATTCCATGTGTGTGTGGTCGTGATGCTACACAA**

**TATCTAGTACAACAAGAGTCTTCTTTTGTTATGATGTCTGCACCACCTGCTGAGTATAAA**

**TTACAGCAAGGTACATTCTTATGTGCGAATGAGTACACTGGTAACTATCAGTGTGGTCAT**

**TACACTCATATAACTGCTAAGGAGACCCTCTATCGTATTGACGGAGCTCACCTTACAAAG**

**ATGTCAGAGTACAAAGGACCAGTGACTGATGTTTTCTACAAGGAAACATCTTACACTACA**

**ACCATCAAGCCTGTGTCGTATAAACTCGATGGAGTTACTTACACAGAGATTGAACCAAAA**

**TTGGATGGGTATTATAAAAAGGATAATGCTTACTATACAGAGCAGCCTATAGACCTTGTA**

**CCAACTCAACCATTACCAAATGCGAGTTTTGATAATTTCAAACTCACATGTTCTAACACA**

**AAATTTGCTGATGATTTAAATCAAATGACAGGCTTCACAAAGCCAGCTTCACGAGAGCTA**

**TCTGTCACATTCTTCCCAGACTTGAATGGCGATGTAGTGGCTATTGACTATAGACACTAT**

**TCAGCGAGTTTCAAGAAAGGTGCTAAATTACTGCATAAGCCAATTGTTTGGCACATTAAC**

**CAGGCTACAACCAAGACAACGTTCAAACCAAACACTTGGTGTTTACGTTGTCTTTGGAGT**

**ACAAAGCCAGTAGATACTTCAAATTCATTTGAAGTTCTGGCAGTAGAAGACACACAAGGA**

**ATGGACAATCTTGCTTGTGAAAGTCAACAACCCACCTCTGAAGAAGTAGTGGAAAATCCT**

**ACCATACAGAAGGAAGTCATAGAGTGTGACGTGAAAACTACCGAAGTTGTAGGCAATGTC**

**ATACTTAAACCATCAGATGAAGGTGTTAAAGTAACACAAGAGTTAGGTCATGAGGATCTT**

**ATGGCTGCTTATGTGGAAAACACAAGCATTACCATTAAGAAACCTAATGAGCTTTCACTA**

**GCCTTAGGTTTAAAAACAATTGCCACTCATGGTATTGCTGCAATTAATAGTGTTCCTTGG**

**AGTAAAATTTTGGCTTATGTCAAACCATTCTTAGGACAAGCAGCAATTACAACATCAAAT**

**TGCGCTAAGAGATTAGCACAACGTGTGTTTAACAATTATATGCCTTATGTGTTTACATTA**

**TTGTTCCAATTGTGTACTTTTACTAAAAGTACCAATTCTAGAATTAGAGCTTCACTACCT**

**ACAACTATTGCTAAAAATAGTGTTAAGAGTGTTGCTAAATTATGTTTGGATGCCGGCATT**

**AATTATGTGAAGTCACCCAAATTTTCTAAATTGTTCACAATCGCTATGTGGCTATTGTTG**

**TTAAGTATTTGCTTAGGTTCTCTAATCTGTGTAACTGCTGCTTTTGGTGTACTCTTATCT**

**AATTTTGGTGCTCCTTCTTATTGTAATGGCGTTAGAGAATTGTATCTTAATTCGTCTAAC**

**GTTACTACTATGGATTTCTGTGAAGGTTCTTTTCCTTGCAGCATTTGTTTAAGTGGATTA**

**GACTCCCTTGATTCTTATCCAGCTCTTGAAACCATTCAGGTGACGATTTCATCGTACAAG**

**CTAGACTTGACAATTTTAGGTCTGGCCGCTGAGTGGGTTTTGGCATATATGTTGTTCACA**

**AAATTCTTTTATTTATTAGGTCTTTCAGCTATAATGCAGGTGTTCTTTGGCTATTTTGCT**

**AGTCATTTCATCAGCAATTCTTGGCTCATGTGGTTTATCATTAGTATTGTACAAATGGCA**

**CCCGTTTCTGCAATGGTTAGGATGTACATCTTCTTTGCTTCTTTCTACTACATATGGAAG**

**AGCTATGTTCATATCATGGATGGTTGCACCTCTTCGACTTGCATGATGTGCTATAAGCGC**

**AATCGTGCCACACGCGTTGAGTGTACAACTATTGTTAATGGCATGAAGAGATCTTTCTAT**

**GTCTATGCAAATGGAGGCCGTGGCTTCTGCAAGACTCACAATTGGAATTGTCTCAATTGT**

**GACACATTTTGCACTGGTAGTACATTCATTAGTGATGAAGTTGCTCGTGATTTGTCACTC**

**CAGTTTAAAAGACCAATCAACCCTACTGACCAGTCATCGTATATTGTTGATAGTGTTGCT**

**GTGAAAAATGGCGCGCTTCACCTCTACTTTGACAAGGCTGGTCAAAAGACCTATGAGAGA**

**CATCCGCTCTCCCATTTTGTCAATTTAGACAATTTGAGAGCTAACAACACTAAAGGTTCA**

**CTGCCTATTAATGTCATAGTTTTTGATGGCAAGTCCAAATGCGACGAGTCTGCTTCTAAG**

**TCTGCTTCTGTGTACTACAGTCAGCTGATGTGCCAACCTATTCTGTTGCTTGACCAAGCT**

**CTTGTATCAGACGTTGGAGATAGTACTGAAGTTTCCGTTAAGATGTTTGATGCTTATGTC**

**GACACCTTTTCAGCAACTTTTAGTGTTCCTATGGAAAAACTTAAGGCACTTGTTGCTACA**

**GCTCACAGCGAGTTAGCAAAGGGTGTAGCTTTAGATGGTGTCCTTTCTACATTCGTGTCA**

**GCTGCCCGACAAGGTGTTGTTGATACCGATGTTGACACAAAGGATGTTATTGAATGTCTC**

**AAACTTTCACATCACTCTGACTTAGAAGTGACAGGTGACAGTTGTAACAATTTCATGCTC**

**ACCTATAATAAGGTTGAAAACATGACGCCCAGAGATCTTGGCGCATGTATTGACTGTAAT**

**GCAAGGCATATCAATGCCCAAGTAGCAAAAAGTCACAATGTTTCACTCATCTGGAATGTA**

**AAAGACTACATGTCTTTATCTGAACAGCTGCGTAAACAAATTCGTAGTGCTGCCAAGAAG**

**AACAACATACCTTTTAGACTAACTTGTGCTACAACTAGACAGGTTGTCAATGTCATAACT**

**ACTAAAATCTCACTCAAGGGTGGTAAGATTGTTAGTACTTGTTTTAAACTTATGCTTAAG**

**GCCACATTATTGTGCGTTCTTGCTGCATTGGTTTGTTATATCGTTATGCCAGTACATACA**

**TTGTCAATCCATGATGGTTACACAAATGAAATCATTGGTTACAAAGCCATTCAGGATGGT**

**GTCACTCGTGACATCATTTCTACTGATGATTGTTTTGCAAATAAACATGCTGGTTTTGAC**

**GCATGGTTTAGCCAGCGTGGTGGTTCATACAAAAATGACAAAAGCTGCCCTGTAGTAGCT**

**GCTATCATTACAAGAGAGATTGGTTTCATAGTGCCTGGCTTACCGGGTACTGTGCTGAGA**

**GCAATCAATGGTGACTTCTTGCATTTTCTACCTCGTGTTTTTAGTGCTGTTGGCAACATT**

**TGCTACACACCTTCCAAACTCATTGAGTATAGTGATTTTGCTACCTCTGCTTGCGTTCTT**

**GCTGCTGAGTGTACAATTTTTAAGGATGCTATGGGCAAACCTGTGCCATATTGTTATGAC**

**ACTAATTTGCTAGAGGGTTCTATTTCTTATAGTGAGCTTCGTCCAGACACTCGTTATGTG**

**CTTATGGATGGTTCCATCATACAGTTTCCTAACACTTACCTGGAGGGTTCTGTTAGAGTA**

**GTAACAACTTTTGATGCTGAGTACTGTAGACATGGTACATGCGAAAGGTCAGAAGTAGGT**

**ATTTGCCTATCTACCAGTGGTAGATGGGTTCTTAATAATGAGCATTACAGAGCTCTATCA**

**GGAGTTTTCTGTGGTGTTGATGCGATGAATCTCATAGCTAACATCTTTACTCCTCTTGTG**

**CAACCTGTGGGTGCTTTAGATGTGTCTGCTTCAGTAGTGGCTGGTGGTATTATTGCCATA**

**TTGGTGACTTGTGCTGCCTACTACTTTATGAAATTCAGACGTGTTTTTGGTGAGTACAAC**

**CATGTTGTTGCTGCTAATGCACTTTTGTTTTTGATGTCTTTCACTATACTCTGTCTGGTA**

**CCAGCTTACAGCTTTCTGCCGGGAGTCTACTCAGTCTTTTACTTGTACTTGACATTCTAT**

**TTCACCAATGATGTTTCATTCTTGGCTCACCTTCAATGGTTTGCCATGTTTTCTCCTATT**

**GTGCCTTTTTGGATAACAGCAATCTATGTATTCTGTATTTCTCTGAAGCACTGCCATTGG**

**TTCTTTAACAACTATCTTAGGAAAAGAGTCATGTTTAATGGAGTTACATTTAGTACCTTC**

**GAGGAGGCTGCTTTGTGTACCTTTTTGCTCAACAAGGAAATGTACCTAAAATTGCGTAGC**

**GAGACACTGTTGCCACTTACACAGTATAACAGGTATCTTGCTCTATATAACAAGTACAAG**

**TATTTCAGTGGAGCCTTAGATACTACCAGCTATCGTGAAGCAGCTTGCTGCCACTTAGCA**

**AAGGCTCTAAATGACTTTAGCAACTCAGGTGCTGATGTTCTCTACCAACCACCACAGACA**

**TCAATCACTTCTGCTGTTCTGCAGAGTGGTTTTAGGAAAATGGCATTCCCGTCAGGCAAA**

**GTTGAAGGGTGCATGGTACAAGTAACCTGTGGAACTACAACTCTTAATGGATTGTGGTTG**

**GATGACACAGTATACTGTCCAAGACATGTCATTTGCACAGCAGAAGACATGCTTAATCCT**

**AACTATGAAGATCTGCTCATTCGCAAATCCAACCATAGCTTTCTTGTTCAGGCTGGCAAT**

**GTTCAACTTCGTGTTATTGGCCATTCTATGCAAAATTGTCTGCTTAGGCTTAAAGTTGAT**

**ACTTCTAACCCTAAGACACCCAAGTATAAATTTGTCCGTATCCAACCTGGTCAAACATTT**

**TCAGTTCTAGCATGCTACAATGGTTCACCATCTGGTGTTTATCAGTGTGCCATGAGACCT**

**AATCATACCATTAAAGGTTCTTTCCTTAATGGATCATGTGGTAGTGTTGGTTTTAACATT**

**GATTATGATTGCGTGTCTTTCTGCTATATGCATCATATGGAGCTTCCAACAGGAGTACAC**

**GCTGGTACTGACTTAGAAGGTAAATTCTATGGTCCATTTGTTGACAGACAAACTGCACAG**

**GCTGCAGGTACAGACACAACCATAACATTAAATGTTTTGGCATGGCTGTATGCTGCTGTT**

**ATCAATGGTGATAGGTGGTTTCTTAATAGATTCACCACTACTTTGAATGACTTTAACCTT**

**GTGGCAATGAAGTACAACTATGAACCTTTGACACAAGATCATGTTGACATATTGGGACCT**

**CTTTCTGCTCAAACAGGAATTGCCGTCTTAGATATGTGTGCTGCTTTGAAAGAGCTGCTG**

**CAGAATGGTATGAATGGTCGTACTATCCTTGGTAGCACTATTTTAGAAGATGAGTTTACA**

**CCATTTGATGTTGTTAGACAATGCTCTGGTGTTACCTTCCAAGGTAAGTTCAAGAAAATT**

**GTTAAGGGCACTCATCATTGGATGCTTTTAACTTTCTTGACATCACTATTGATTCTTGTT**

**CAAAGTACACAGTGGTCACTGTTTTTCTTTGTTTACGAGAATGCTTTCTTGCCATTTACT**

**CTTGGTATTATGGCAATTGCTGCATGTGCTATGCTGCTTGTTAAGCATAAGCACGCATTC**

**TTGTGCTTGTTTCTGTTACCTTCTCTTGCAACAGTTGCTTACTTTAATATGGTCTACATG**

**CCTGCTAGCTGGGTGATGCGTATCATGACATGGCTTGAATTGGCTGACACTAGCTTGTCT**

**GGTTATAGGCTTAAGGATTGTGTTATGTATGCTTCAGCTTTAGTTTTGCTTATTCTCATG**

**ACAGCTCGCACTGTTTATGATGATGCTGCTAGACGTGTTTGGACACTGATGAATGTCATT**

**ACACTTGTTTACAAAGTCTACTATGGTAATGCTTTAGATCAAGCTATTTCCATGTGGGCC**

**TTAGTTATTTCTGTAACCTCTAACTATTCTGGTGTCGTTACGACTATCATGTTTTTAGCT**

**AGAGCTATAGTGTTTGTGTGTGTTGAGTATTACCCATTGTTATTTATTACTGGCAACACC**

**TTACAGTGTATCATGCTTGTTTATTGTTTCTTAGGCTATTGTTGCTGCTGCTACTTTGGC**

**CTTTTCTGTTTACTCAACCGTTACTTCAGGCTTACTCTTGGTGTTTATGACTACTTGGTC**

**TCTACACAAGAATTTAGGTATATGAACTCCCAGGGGCTTTTGCCTCCTAAGAGTAGTATT**

**GATGCTTTCAAGCTTAACATTAAGTTGTTGGGTATTGGAGGTAAACCATGTATCAAGGTT**

**GCTACTGTACAGTCTAAAATGTCTGACGTAAAGTGCACATCTGTGGTACTGCTCTCGGTT**

**CTTCAACAACTTAGAGTAGAGTCATCTTCTAAATTGTGGGCACAATGTGTACAACTCCAC**

**AATGATATTCTTCTTGCAAAAGACACAACTGAAGCTTTCGAGAAGATGGTTTCTCTTTTG**

**TCTGTTTTGCTATCCATGCAGGGTGCTGTAGACATTAATAGGTTGTGCGAGGAAATGCTC**

**GATAACCGTGCTACTCTTCAGGCTATTGCTTCAGAATTTAGTTCTTTACCATCATATGCC**

**GCTTATGCCACTGCCCAGGAGGCCTATGAGCAGGCTGTAGCTAATGGTGATTCTGAAGTC**

**GTTCTCAAAAAGTTAAAGAAATCTTTGAATGTGGCTAAATCTGAGTTTGACCGTGATGCT**

**GCCATGCAACGCAAGTTGGAAAAGATGGCAGATCAGGCTATGACCCAAATGTACAAACAG**

**GCAAGATCTGAGGACAAGAGGGCAAAAGTAACTAGTGCTATGCAAACAATGCTCTTCACT**

**ATGCTTAGGAAGCTTGATAATGATGCACTTAACAACATTATCAACAATGCGCGTGATGGT**

**TGTGTTCCACTCAACATCATACCATTGACTACAGCAGCCAAACTCATGGTTGTTGTCCCT**

**GATTATGGTACCTACAAGAACACTTGTGATGGTAACACCTTTACATATGCATCTGCACTC**

**TGGGAAATCCAGCAAGTTGTTGATGCGGATAGCAAGATTGTTCAACTTAGTGAAATTAAC**

**ATGGACAATTCACCAAATTTGGCTTGGCCTCTTATTGTTACAGCTCTAAGAGCCAACTCA**

**GCTGTTAAACTACAGAATAATGAACTGAGTCCAGTAGCACTACGACAGATGTCCTGTGCG**

**GCTGGTACCACACAAACAGCTTGTACTGATGACAATGCACTTGCCTACTATAACAATTCG**

**AAGGGAGGTAGGTTTGTGCTGGCATTACTATCAGACCACCAAGATCTCAAATGGGCTAGA**

**TTCCCTAAGAGTGATGGTACAGGTACAATTTACACAGAACTGGAACCACCTTGTAGGTTT**

**GTTACAGACACACCAAAAGGGCCTAAAGTGAAATACTTGTACTTCATCAAAGGCTTAAAC**

**AACCTAAATAGAGGTATGGTGCTGGGCAGTTTAGCTGCTACAGTACGTCTTCAGGCTGGA**

**AATGCTACAGAAGTACCTGCCAATTCAACTGTGCTTTCCTTCTGTGCTTTTGCAGTAGAC**

**CCTGCTAAAGCATATAAGGATTACCTAGCAAGTGGAGGACAACCAATCACCAACTGTGTG**

**AAGATGTTGTGTACACACACTGGTACAGGACAGGCAATTACTGTAACACCAGAAGCTAAC**

**ATGGACCAAGAGTCCTTTGGTGGTGCTTCATGTTGTCTGTATTGTAGATGCCACATTGAC**

**CATCCAAATCCTAAAGGATTCTGTGACTTGAAAGGTAAGTACGTCCAAATACCTACCACT**

**TGTGCTAATGACCCAGTGGGTTTTACACTTAGAAACACAGTCTGTACCGTCTGCGGAATG**

**TGGAAAGGTTATGGCTGTAGTTGTGACCAACTCCGCGAACCCTTGATGCAGTCTGCGGAT**

**GCATCAACGTTTTTAAACGGGTTTGCGGTGTAAGTGCAGCCCGTCTTACACCGTGCGGCA**

**CAGGCACTAGTACTGATGTCGTCTACAGGGCTTTTGATATTTACAACGAAAAAGTTGCTG**

**GTTTTGCAAAGTTCCTAAAAACTAATTGCTGTCGCTTCCAGGAGAAGGATGAGGAAGGCA**

**ATTTATTAGACTCTTACTTTGTAGTTAAGAGGCATACTATGTCTAACTACCAACATGAAG**

**AGACTATTTATAACTTGGTTAAAGATTGTCCAGCGGTTGCTGTCCATGACTTTTTCAAGT**

**TTAGAGTAGATGGTGACATGGTACCACATATATCACGTCAGCGTCTAACTAAATACACAA**

**TGGCTGATTTAGTCTATGCTCTACGTCATTTTGATGAGGGTAATTGTGATACATTAAAAG**

**AAATACTCGTCACATACAATTGCTGTGATGATGATTATTTCAATAAGAAGGATTGGTATG**

**ACTTCGTAGAGAATCCTGACATCTTACGCGTATATGCTAACTTAGGTGAGCGTGTACGCC**

**AATCATTATTAAAGACTGTACAATTCTGCGATGCTATGCGTGATGCAGGCATTGTAGGCG**

**TACTGACATTAGATAATCAGGATCTTAATGGGAACTGGTACGATTTCGGTGATTTCGTAC**

**AAGTAGCACCAGGCTGCGGAGTTCCTATTGTGGATTCATATTACTCATTGCTGATGCCCA**

**TCCTCACTTTGACTAGGGCATTGGCTGCTGAGTCCCATATGGATGCTGATCTCGCAAAAC**

**CACTTATTAAGTGGGATTTGCTGAAATATGATTTTACGGAAGAGAGACTTTGTCTCTTCG**

**ACCGTTATTTTAAATATTGGGACCAGACATACCATCCCAATTGTATTAACTGTTTGGATG**

**ATAGGTGTATCCTTCATTGTGCAAACTTTAATGTGTTATTTTCTACTGTGTTTCCACCTA**

**CAAGTTTTGGACCACTAGTAAGAAAAATATTTGTAGATGGTGTTCCTTTTGTTGTTTCAA**

**CTGGATACCATTTTCGTGAGTTAGGAGTCGTACATAATCAGGATGTAAACTTACATAGCT**

**CGCGTCTCAGTTTCAAGGAACTTTTAGTGTATGCTGCTGATCCAGCTATGCATGCAGCTT**

**CTGGCAATTTATTGCTAGATAAACGCACTACATGCTTTTCAGTAGCTGCACTAACAAACA**

**ATGTTGCTTTTCAAACTGTCAAACCCGGTAATTTTAATAAAGACTTTTATGACTTTGCTG**

**TGTCTAAAGGTTTCTTTAAGGAAGGAAGTTCTGTTGAACTAAAACACTTCTTCTTTGCTC**

**AGGATGGCAACGCTGCTATCAGTGATTATGACTATTATCGTTATAATCTGCCAACAATGT**

**GTGATATCAGACAACTCCTATTCGTAGTTGAAGTTGTTGATAAATACTTTGATTGTTACG**

**ATGGTGGCTGTATTAATGCCAACCAAGTAATCGTTAACAATCTGGATAAATCAGCTGGTT**

**TCCCATTTAATAAATGGGGTAAGGCTAGACTTTATTATGACTCAATGAGTTATGAGGATC**

**AAGATGCACTTTTCGCGTATACTAAGCGTAATGTCATCCCTACTATAACTCAAATGAATC**

**TTAAGTATGCCATTAGTGCAAAGAATAGAGCTCGCACCGTAGCTGGTGTCTCTATCTGTA**

**GTACTATGACAAATAGACAGTTTCATCAGAAATTATTGAAGTCAATAGCCGCCACTAGAG**

**GAGCTACTGTGGTAATTGGAACAAGCAAGTTTTACGGTGGCTGGCATAATATGTTAAAAA**

**CTGTTTACAGTGATGTAGAAACTCCACACCTTATGGGTTGGGATTATCCAAAATGTGACA**

**GAGCCATGCCTAACATGCTTAGGATAATGGCCTCTCTTGTTCTTGCTCGCAAACATAACA**

**CTTGCTGTAACTTATCACACCGTTTCTACAGGTTAGCTAACGAGTGTGCGCAAGTATTAA**

**GTGAGATGGTCATGTGTGGCGGCTCACTATATGTTAAACCAGGTGGAACATCATCCGGTG**

**ATGCTACAACTGCTTATGCTAATAGTGTCTTTAACATTTGTCAAGCTGTTACAGCCAATG**

**TAAATGCACTTCTTTCAACTGATGGTAATAAGATAGCTGACAAGTATGTCCGCAATCTAC**

**AACACAGGCTCTATGAGTGTCTCTATAGAAATAGGGATGTTGATCATGAATTCGTGGATG**

**AGTTTTACGCTTACCTGCGTAAACATTTCTCCATGATGATTCTTTCTGATGATGCCGTTG**

**TGTGCTATAACAGTAACTATGCGGCTCAAGGTTTAGTAGCTAGCATTAAGAACTTTAAGG**

**CAGTTCTTTATTATCAAAATAATGTGTTCATGTCTGAGGCAAAATGTTGGACTGAGACTG**

**ACCTTACTAAAGGACCTCACGAATTTTGCTCACAGCATACAATGCTAGTTAAACAAGGAG**

**ATGATTACGTGTACCTGCCTTACCCAGATCCATCAAGAATATTAGGCGCAGGCTGTTTTG**

**TCGATGATATTGTCAAAACAGATGGTACACTTATGATTGAAAGGTTCGTGTCACTGGCTA**

**TTGATGCTTACCCACTTACAAAACATCCTAATCAGGAGTATGCTGATGTCTTTCACTTGT**

**ATTTACAATACATTAGAAAGTTACATGATGAGCTTACTGGCCACATGTTGGACATGTATT**

**CCGTAATGCTAACTAATGATAACACCTCACGGTACTGGGAACCTGAGTTTTATGAGGCTA**

**TGTACACACCACATACAGTCTTGCAGGCTGTAGGTGCTTGTGTATTGTGCAATTCACAGA**

**CTTCACTTCGTTGCGGTGCCTGTATTAGGAGACCATTCCTATGTTGCAAGTGCTGCTATG**

**ACCATGTCATTTCAACATCACACAAATTAGTGTTGTCTGTTAATCCCTATGTTTGCAATG**

**CCCCAGGTTGTGATGTCACTGATGTGACACAACTGTATCTAGGAGGTATGAGCTATTATT**

**GCAAGTCACATAAGCCTCCCATTAGTTTTCCATTATGTGCTAATGGTCAGGTTTTTGGTT**

**TATACAAAAACACATGTGTAGGCAGTGACAATGTCACTGACTTCAATGCGATAGCAACAT**

**GTGATTGGACTAATGCTGGCGATTACATACTTGCCAACACTTGTACTGAGAGACTCAAGC**

**TTTTCGCAGCAGAAACGCTCAAAGCCACTGAGGAAACATTTAAGCTGTCATATGGTATTG**

**CCACTGTACGCGAAGTACTCTCTGACAGAGAATTGCATCTTTCATGGGAGGTTGGAAAAC**

**CTAGACCACCATTGAACAGAAACTATGTCTTTACTGGTTACCGTGTAACTAAAAATAGTA**

**AAGTACAGATTGGAGAGTACACCTTTGAAAAAGGTGACTATGGTGATGCTGTTGTGTACA**

**GAGGTACTACGACATACAAGTTGAATGTTGGTGATTACTTTGTGTTGACATCTCACACTG**

**TAATGCCACTTAGTGCACCTACTCTAGTGCCACAAGAGCACTATGTGAGAATTACTGGCT**

**TGTACCCAACACTCAACATCTCAGATGAGTTTTCTAGCAATGTTGCAAATTATCAAAAGG**

**TCGGCATGCAAAAGTACTCTACACTCCAAGGACCACCTGGTACTGGTAAGAGTCATTTTG**

**CCATCGGACTTGCTCTCTATTACCCATCTGCTCGCATAGTGTATACGGCATGCTCTCATG**

**CAGCTGTTGATGCCCTATGTGAAAAGGCATTAAAATATTTGCCCATAGATAAATGTAGTA**

**GAATCATACCTGCGCGTGCGCGCGTAGAGTGTTTTGATAAATTCAAAGTGAATTCAACAC**

**TAGAACAGTATGTTTTCTGCACTGTAAATGCATTGCCAGAAACAACTGCTGACATTGTAG**

**TCTTTGATGAAATCTCTATGGCTACTAATTATGACTTGAGTGTTGTCAATGCTAGACTTC**

**GTGCAAAACACTACGTCTATATTGGCGATCCTGCTCAATTACCAGCCCCCCGCACATTGC**

**TGACTAAAGGCACACTAGAACCAGAATATTTTAATTCAGTGTGCAGACTTATGAAAACAA**

**TAGGTCCAGACATGTTCCTTGGAACTTGTCGCCGTTGTCCTGCTGAAATTGTTGACACTG**

**TGAGTGCTTTAGTTTATGACAATAAGCTAAAAGCACACAAGGATAAGTCAGCTCAATGCT**

**TCAAAATGTTCTACAAAGGTGTTATTACACATGATGTTTCATCTGCAATCAACAGACCTC**

**AAATAGGCGTTGTAAGAGAATTTCTTACACGCAATCCTGCTTGGAGAAAAGCTGTTTTTA**

**TCTCACCTTATAATTCACAGAACGCTGTAGCTTCAAAAATCTTAGGATTGCCTACGCAGA**

**CTGTTGATTCATCACAGGGTTCTGAATATGACTATGTCATATTCACACAAACTACTGAAA**

**CAGCACACTCTTGTAATGTCAACCGCTTCAATGTGGCTATCACAAGGGCAAAAATTGGCA**

**TTTTGTGCATAATGTCTGATAGAGATCTTTATGACAAACTGCAATTTACAAGTCTAGAAA**

**TACCACGTCGCAATGTGGCTACATTACAAGCAGAAAATGTAACTGGACTTTTTAAGGACT**

**GTAGTAAGATCATTACTGGTCTTCATCCTACACAGGCACCTACACACCTCAGCGTTGATA**

**TAAAGTTCAAGACTGAAGGATTATGTGTTGACATACCAGGCATACCAAAGGACATGACCT**

**ACCGTAGACTCATCTCTATGATGGGTTTCAAAATGAATTACCAAGTCAATGGTTACCCTA**

**ATATGTTTATCACCCGCGAAGAAGCTATTCGTCACGTTCGTGCGTGGATTGGCTTTGATG**

**TAGAGGGCTGTCATGCAACTAGAGATGCTGTGGGTACTAACCTACCTCTCCAGCTAGGAT**

**TTTCTACAGGTGTTAACTTAGTAGCTGTACCGACTGGTTATGTTGACACTGAAAATAACA**

**CAGAATTCACCAGAGTTAATGCAAAACCTCCACCAGGTGACCAGTTTAAACATCTTATAC**

**CACTCATGTATAAAGGCTTGCCCTGGAATGTAGTGCGTATTAAGATAGTACAAATGCTCA**

**GTGATACACTGAAAGGATTGTCAGACAGAGTCGTGTTCGTCCTTTGGGCGCATGGCTTTG**

**AGCTTACATCAATGAAGTACTTTGTCAAGATTGGACCTGAAAGAACGTGTTGTCTGTGTG**

**ACAAACGTGCAACTTGCTTTTCTACTTCATCAGATACTTATGCCTGCTGGAATCATTCTG**

**TGGGTTTTGACTATGTCTATAACCCATTTATGATTGATGTTCAGCAGTGGGGCTTTACGG**

**GTAACCTTCAGAGTAACCATGACCAACATTGCCAGGTACATGGAAATGCACATGTGGCTA**

**GTTGTGATGCTATCATGACTAGATGTTTAGCAGTCCATGAGTGCTTTGTTAAGCGCGTTG**

**ATTGGTCTGTTGAATACCCTATTATAGGAGATGAACTGAGGGTTAATTCTGCTTGCAGAA**

**AAGTACAACACATGGTTGTGAAGTCTGCATTGCTTGCTGATAAGTTTCCAGTTCTTCATG**

**ACATTGGAAATCCAAAGGCTATCAAGTGTGTGCCTCAGGCTGAAGTAGAATGGAAGTTCT**

**ACGATGCTCAGCCATGTAGTGACAAAGCTTACAAAATAGAGGAACTCTTCTATTCTTATG**

**CTACACATCACGATAAATTCACTGATGGTGTTTGTTTGTTTTGGAATTGTAACGTTGATC**

**GTTACCCAGCCAATGCAATTGTGTGTAGGTTTGACACAAGAGTCTTGTCAAACTTGAACT**

**TACCAGGCTGTGATGGTGGTAGTTTGTATGTGAATAAGCATGCATTCCACACTCCAGCTT**

**TCGATAAAAGTGCATTTACTAATTTAAAGCAATTGCCTTTCTTTTACTATTCTGATAGTC**

**CTTGTGAGTCTCATGGCAAACAAGTAGTGTCGGATATTGATTATGTTCCACTCAAATCTG**

**CTACGTGTATTACACGATGCAATTTAGGTGGTGCTGTTTGCAGACACCATGCAAATGAGT**

**ACCGACAGTACTTGGATGCATATAATATGATGATTTCTGCTGGATTTAGCCTATGGATTT**

**ACAAACAATTTGATACTTATAACCTGTGGAATACATTTACCAGGTTACAGAGTTTAGAAA**

**ATGTGGCTTATAATGTTGTTAATAAAGGACACTTTGATGGACACGCCGGCGAAGCACCTG**

**TTTCCATCATTAATAATGCTGTTTACACAAAGGTAGATGGTATTGATGTGGAGATCTTTG**

**AAAATAAGACAACACTTCCTGTTAATGTTGCATTTGAGCTTTGGGCTAAGCGTAACATTA**

**AACCAGTGCCAGAGATTAAGATACTCAATAATTTGGGTGTTGATATCGCTGCTAATACTG**

**TAATCTGGGACTACAAAAGAGAAGCCCCAGCACATGTATCTACAATAGGTGTCTGCACAA**

**TGACTGACATTGCCAAGAAACCTACTGAGAGTGCTTGTTCTTCACTTACTGTCTTGTTTG**

**ATGGTAGAGTGGAAGGACAGGTAGACCTTTTTAGAAACGCCCGTAATGGTGTTTTAATAA**

**CAGAAGGTTCAGTCAAAGGTCTAACACCTTCAAAGGGACCAGCACAAGCTAGCGTCAATG**

**GAGTCACATTAATTGGAGAATCAGTAAAAACACAGTTTAACTACTTTAAGAAAGTAGACG**

**GCATTATTCAACAGTTGCCTGAAACCTACTTTACTCAGAGCAGAGACTTAGAGGATTTTA**

**AGCCCAGATCACAAATGGAAACTGACTTTCTCGAGCTCGCTATGGATGAATTCATACAGC**

**GATATAAGCTCGAGGGCTATGCCTTCGAACACATCGTTTATGGAGATTTCAGTCATGGAC**

**AACTTGGCGGTCTTCATTTAATGATAGGCTTAGCCAAGCGCTCACAAGATTCACCACTTA**

**AATTAGAGGATTTTATCCCTATGGACAGCACAGTGAAAAATTACTTCATAACAGATGCGC**

**AAACAGGTTCATCAAAATGTGTGTGTTCTGTGATTGATCTTTTACTTGATGACTTTGTCG**

**AGATAATAAAGTCACAAGATTTGTCAGTGATTTCAAAAGTGGTCAAGGTTACAATTGACT**

**ATGCTGAAATTTCATTCATGCTTTGGTGTAAGGATGGACATGTTGAAACCTTCTACCCAA**

**AACTACAAGCAAGTCAAGCGTGGCAACCAGGTGTTGCGATGCCTAACTTGTACAAGATGC**

**AAAGAATGCTTCTTGAAAAGTGTGACCTTCAGAATTATGGTGAAAATGCTGTTATACCAA**

**AAGGAATAATGATGAATGTCGCAAAGTATACTCAACTGTGTCAATACTTAAATACACTTA**

**CTTTAGCTGTACCCTACAACATGAGAGTTATTCACTTTGGTGCTGGCTCTGATAAAGGAG**

**TTGCACCAGGTACAGCTGTGCTCAGACAATGGTTGCCAACTGGCACACTACTTGTCGATT**

**CAGATCTTAATGACTTCGTCTCCGACGCAGATTCTACTTTAATTGGAGACTGTGCAACAG**

**TACATACGGCTAATAAATGGGACCTTATTATTAGCGATATGTATGACCCTAGGACCAAAC**

**ATGTGACAAAAGAGAATGACTCTAAAGAAGGGTTTTTCACTTATCTGTGTGGATTTATAA**

**AGCAAAAACTAGCCCTGGGTGGTTCTATAGCTGTAAAGATAACAGAGCATTCTTGGAATG**

**CTGACCTTTACAAGCTTATGGGCCATTTCTCATGGTGGACAGCTTTTGTTACAAATGTAA**

**ATGCATCATCATCGGAAGCATTTTTAATTGGGGCTAACTATCTTGGCAAGCCGAAGGAAC**

**AAATTGATGGCTATACCATGCATGCTAACTACATTTTCTGGAGGAACACAAATCCTATCC**

**AGTTGTCTTCCTATTCACTCTTTGACATGAGCAAATTTCCTCTTAAATTAAGAGGAACTG**

**CTGTAATGTCTCTTAAGGAGAATCAAATCAATGATATGATTTATTCTCTTCTGGAAAAAG**

**GTAGGCTTATCATTAGAGAAAACAACAGAGTTGTGGTTTCAAGTGATATTCTTGTTAACA**

**ACTAAACGAACATGTTTATTTTCTTATTATTTCTTACTCTCACTAGTGGTAGTGACCTTG**

**ACCGGTGCACCACTTTTGATGATGTTCAAGCTCCTAATTACACTCAACATACTTCATCTA**

**TGAGGGGGGTTTACTATCCTGATGAAATTTTTAGATCAGACACTCTTTATTTAACTCAGG**

**ATTTATTTCTTCCATTTTATTCTAATGTTACAGGGTTTCATACTATTAATCATACGTTTG**

**GCAACCCTGTCATACCTTTTAAGGATGGTATTTATTTTGCTGCCACAGAGAAATCAAATG**

**TTGTCCGTGGTTGGGTTTTTGGTTCTACCATGAACAACAAGTCACAGTCGGTGATTATTA**

**TTAACAATTCTACTAATGTTGTTATACGAGCATGTAACTTTGAATTGTGTGACAACCCTT**

**TCTTTGCTGTTTCTAAACCCATGGGTACACAGACACATACTATGATATTCGATAATGCAT**

**TTAATTGCACTTTCGAGTACATATCTGATGCCTTTTCGCTTGATGTTTCAGAAAAGTCAG**

**GTAATTTTAAACACTTACGAGAGTTTGTGTTTAAAAATAAAGATGGGTTTCTCTATGTTT**

**ATAAGGGCTATCAACCTATAGATGTAGTTCGTGATCTACCTTCTGGTTTTAACACTTTGA**

**AACCTATTTTTAAGTTGCCTCTTGGTATTAACATTACAAATTTTAGAGCCATTCTTACAG**

**CCTTTTCACCTGCTCAAGACATTTGGGGCACGTCAGCTGCAGCCTATTTTGTTGGCTATT**

**TAAAGCCAACTACATTTATGCTCAAGTATGATGAAAATGGTACAATCACAGATGCTGTTG**

**ATTGTTCTCAAAATCCACTTGCTGAACTCAAATGCTCTGTTAAGAGCTTTGAGATTGACA**

**AAGGAATTTACCAGACCTCTAATTTCAGGGTTGTTCCCTCAGGAGATGTTGTGAGATTCC**

**CTAATATTACAAACTTGTGTCCTTTTGGAGAGGTTTTTAATGCTACTAAATTCCCTTCTG**

**TCTATGCATGGGAGAGAAAAAAAATTTCTAATTGTGTTGCTGATTACTCTGTGCTCTACA**

**ACTCAACATTTTTTTCAACCTTTAAGTGCTATGGCGTTTCTGCCACTAAGTTGAATGATC**

**TTTGCTTCTCCAATGTCTATGCAGATTCTTTTGTAGTCAAGGGAGATGATGTAAGACAAA**

**TAGCGCCAGGACAAACTGGTGTTATTGCTGATTATAATTATAAATTGCCAGATGATTTCA**

**TGGGTTGTGTCCTTGCTTGGAATACTAGGAACATTGATGCTACTTCAACTGGTAATTATA**

**ATTATAAATATAGGTATCTTAGACATGGCAAGCTTAGGCCCTTTGAGAGAGACATATCTA**

**ATGTGCCTTTCTCCCCTGATGGCAAACCTTGCACCCCACCTGCTCTTAATTGTTATTGGC**

**CATTAAATGATTATGGTTTTTACACCACTACTGGCATTGGCTACCAACCTTACAGAGTTG**

**TAGTACTTTCTTTTGAACTTTTAAATGCACCGGCCACGGTTTGTGGACCAAAATTATCCA**

**CTGACCTTATTAAGAACCAGTGTGTCAATTTTAATTTTAATGGACTCACTGGTACTGGTG**

**TGTTAACTCCTTCTTCAAAGAGATTTCAACCATTTCAACAATTTGGCCGTGATGTTTCTG**

**ATTTCACTGATTCCGTTCGAGATCCTAAAACATCTGAAATATTAGACATTTCACCTTGCG**

**CTTTTGGGGGTGTAAGTGTAATTACACCTGGAACAAATGCTTCATCTGAAGTTGCTGTTC**

**TATATCAAGATGTTAACTGCACTGATGTTTCTACAGCAATTCATGCAGATCAACTCACAC**

**CAGCTTGGCGCATATATTCTACTGGAAACAATGTATTCCAGACTCAAGCAGGCTGTCTTA**

**TAGGAGCTGAGCATGTCGACACTTCTTATGAGTGCGACATTCCTATTGGAGCTGGCATTT**

**GTGCTAGTTACCATACAGTTTCTTTATTACGTAGTACTAGCCAAAAATCTATTGTGGCTT**

**ATACTATGTCTTTAGGTGCTGATAGTTCAATTGCTTACTCTAATAACACCATTGCTATAC**

**CTACTAACTTTTCAATTAGCATTACTACAGAAGTAATGCCTGTTTCTATGGCTAAAACCT**

**CCGTAGATTGTAATATGTACATCTGCGGAGATTCTACTGAATGTGCTAATTTGCTTCTCC**

**AATATGGTAGCTTTTGCACACAACTAAATCGTGCACTCTCAGGTATTGCTGCTGAACAGG**

**ATCGCAACACACGTGAAGTGTTCGCTCAAGTCAAACAAATGTACAAAACCCCAACTTTGA**

**AATATTTTGGTGGTTTTAATTTTTCACAAATATTACCTGACCCTCTAAAGCCAACTAAGA**

**GGTCTTTTATTGAGGACTTGCTCTTTAATAAGGTGACACTCGCTGATGCTGGCTTCATGA**

**AGCAATATGGCGAATGCCTAGGTGATATTAATGCTAGAGATCTCATTTGTGCGCAGAAGT**

**TCAATGGACTTACAGTGTTGCCACCTCTGCTCACTGATGATATGATTGCTGCCTACACTG**

**CTGCTCTAGTTAGTGGTACTGCCACTGCTGGATGGACATTTGGTGCTGGCGCTGCTCTTC**

**AAATACCTTTTGCTATGCAAATGGCATATAGGTTCAATGGCATTGGAGTTACCCAAAATG**

**TTCTCTATGAGAACCAAAAACAAATCGCCAACCAATTTAACAAGGCGATTAGTCAAATTC**

**AAGAATCACTTACAACAACATCAACTGCATTGGGCAAGCTGCAAGACGTTGTTAACCAGA**

**ATGCTCAAGCATTAAACACACTTGTTAAACAACTTAGCTCTAATTTTGGTGCAATTTCAA**

**GTGTGCTAAATGATATCCTTTCGCGACTTGATAAAGTCGAGGCGGAGGTACAAATTGACA**

**GGTTAATTACAGGCAGACTTCAAAGCCTTCAAACCTATGTAACACAACAACTAATCAGGG**

**CTGCTGAAATCAGGGCTTCTGCTAATCTTGCTGCTACTAAAATGTCTGAGTGTGTTCTTG**

**GACAATCAAAAAGAGTTGACTTTTGTGGAAAGGGCTACCACCTTATGTCCTTCCCACAAG**

**CAGCCCCGCATGGTGTTGTCTTCCTACATGTCACGTATGTGCCATCCCAGGAGAGGAACT**

**TCACCACAGCGCCAGCAATTTGTCATGAAGGCAAAGCATACTTCCCTCGTGAAGGTGTTT**

**TTGTGTTTAATGGCACTTCTTGGTTTATTACACAGAGGAACTTCTTTTCTCCACAAATAA**

**TTACTACAGACAATACATTTGTCTCAGGAAATTGTGATGTCGTTATTGGCATCATTAACA**

**ACACAGTTTATGATCCTCTGCAACCTGAGCTTGACTCATTCAAAGAAGAGCTGGACAAGT**

**ACTTCAAAAATCATACATCACCAGATGTTGATCTTGGCGACATTTCAGGCATTAACGCTT**

**CTGTCGTCAACATTCAAAAAGAAATTGACCGCCTCAATGAGGTCGCTAAAAATTTAAATG**

**AATCACTCATTGACCTTCAAGAATTGGGAAAATATGAGCAATATATTAAATGGCCTTGGT**

**ATGTTTGGCTCGGCTTCATTGCTGGACTAATTGCCATCGTCATGGTTACAATCTTGCTTT**

**GTTGCATGACTAGTTGTTGCAGTTGCCTCAAGGGTGCATGCTCTTGTGGTTCTTGCTGCA**

**AGTTTGATGAGGATGACTCTGAGCCAGTTCTCAAGGGTGTCAAATTACATTACACATAAA**

**CGAACTTATGGATTTGTTTATGAGATTTTTTACTCTTAGATCAATTACTGCACAGCCAGT**

**AAAAATTGACAATGCTTCTCCTGCAAGTACTGTTCATGCTACAGCAACGATACCGCTACA**

**AGCCTCACTCCCTTTCGGATGGCTTGTTATTGGCGTTGCATTTCTTGCTGTTTTTCAGAG**

**CGCTACCAAAATAATTGCGCTCAATAAAAGATGGCAGCTAGCCCTTTATAAGGGCTTCCA**

**GTTCATTTGCAATTTACTGCTGCTATTTGTTACCATCTATTCACATCTTTTGCTTGTCGC**

**TGCAGGTATGGAGGCGCAATTTTTGTACCTCTATGCCTTGATATATTTTCTACAATGCAT**

**CAACGCATGTAGAATTATTATGAGATGTTGGCTTTGTTGGAAGTGCAAATCCAAGAACCC**

**ATTACTTTATGATGCCAACTACTTTGTTTGCTGGCACACACATAACTATGACTACTGTAT**

**ACCATATAACAGTGTCACAGATACAATTGTCGTTACTGAAGGTGACGGCATTTCAACACC**

**AAAACTCAAAGAAGACTACCAAATTGGTGGTTATTCTGAGGATAGGCACTCAGGTGTTAA**

**AGACTATGTCGTTGTACATGGCTATTTCACCGAAGTTTACTACCAGCTTGAGTCTACACA**

**AATTACTACAGACACTGGTATTGAAAATGCTACATTCTTCATCTTTAACAAGCTTGTTAA**

**AGACCCACCGAATGTGCAAATACACACAATCGACGGCTCTTCAGGAGTTGCTAATCCAGC**

**AATGGATCCAATTTATGATGAGCCGACGACGACTACTAGCGTGCCTTTGTAAGCACAAGA**

**AAGTGAGTACGAACTTATGTACTCATTCGTTTCGGAAGAAACAGGTACGTTAATAGTTAA**

**TAGCGTACTTCTTTTTCTTGCTTTCGTGGTATTCTTGCTAGTCACACTAGCCATCCTTAC**

**TGCGCTTCGATTGTGTGCGTACTGCTGCAATATTGTTAACGTGAGTTTAGTAAAACCAAC**

**GGTTTACGTCTACTCGCGTGTTAAAAATCTGAACTCTTCTGAAGGAGTTCCTGATCTTCT**

**GGTCTAAACGAACTAACTATTATTATTATTCTGTTTGGAACTTTAACATTGCTTATCATG**

**GCAGACAACGGTACTATTACCGTTGAGGAGCTTAAACAACTCCTGGAACAATGGAACCTA**

**GTAATAGGTTTCCTATTCCTAGCCTGGATTATGTTACTACAATTTGCCTATTCTAATCGG**

**AACAGGTTTTTGTACATAATAAAGCTTGTTTTCCTCTGGCTCTTGTGGCCAGTAACACTT**

**GCTTGTTTTGTGCTTGCTGCTGTCTACAGAATTAATTGGGTGACTGGCGGGATTGCGATT**

**GCAATGGCTTGTATTGTAGGCTTGATGTGGCTTAGCTACTTCGTTGCTTCCTTCAGGCTG**

**TTTGCTCGTACCCGCTCAATGTGGTCATTCAACCCAGAAACAAACATTCTTCTCAATGTG**

**CCTCTCCGGGGGACAATTGTGACCAGACCGCTCATGGAAAGTGAACTTGTCATTGGTGCT**

**GTGATCATTCGTGGTCACTTGCGAATGGCCGGACACTCCCTAGGGCGCTGTGACATTAAG**

**GACCTGCCAAAAGAGATCACTGTGGCTACATCACGAACGCTTTCTTATTACAAATTAGGA**

**GCGTCGCAGCGTGTAGGCACTGATTCAGGTTTTGCTGCATACAACCGCTACCGTATTGGA**

**AACTATAAATTAAATACAGACCACGCCGGTAGCAACGACAATATTGCTTTGCTAGTACAG**

**TAAGTGACAACAGATGTTTCATCTTGTTGACTTCCAGGTTACAATAGCAGAGATATTGAT**

**TATCATTATGAGGACTTTCAGGATTGCTATTTGGAATCTTGACGTTATAATAAGTTCAAT**

**AGTGAGACAATTATTTAAGCCTCTAACTAAGAAGAATTATTCGGAGTTAGATGATGAAGA**

**ACCTATGGAGTTAGATTATCCATAAAACGAACATGAAAATTATTCTCTTCCTGACATTGA**

**TTGTATTTACATCTTGCGAGCTATATCACTATCAGGAGTGTGTTAGAGGTACGACTGTAC**

**TACTAAAAGAACCTTGCCCATCAGGAACATACGAGGGCAATTCACCATTTCACCCTCTTG**

**CTGACAATAAATTTGCACTAACTTGCACTAGCACACACTTTGCTTTTGCTTGTGCTGACG**

**GTACTCGACATACCTATCAGCTGCGTGCAAGATCAGTTTCACCAAAACTTTTCATCAGAC**

**AAGAGGAGGTTCAACAAGAGCTCTACTCGCCACTTTTTCTCATTGTTGCTGCTCTAGTAT**

**TTTTAATACTTTGCTTCACCATTAAGAGAAAGACAGAATGAATGAGCTCACTTTAATTGA**

**CTTCTATTTGTGCTTTTTAGCCTTTCTGCTATTCCTTGTTTTAATAATGCTTATTATATT**

**TTGGTTTTCACTCGAAATCCAGGATCTAGAAGAACCTTGTACCAAAGTCTAAACGAACAT**

**GAAACTTCTCATTGTTTTGACTTGTATTTCTCTATGCAGTTGCATATGCACTGTAGTACA**

**GCGCTGTGCATCTAATAAACCTCATGTGCTTGAAGATCCTTGTAAGGTACAACACTAGGG**

**GTAATACTTATAGCACTGCTTGGCTTTGTGCTCTAGGAAAGGTTTTACCTTTTCATAGAT**

**GGCACACTATGGTTCAAACATGCACACCTAATGTTACTATCAACTGTCAAGATCCAGCTG**

**GTGGTGCGCTTATAGCTAGGTGTTGGTACCTTCATGAAGGTCACCAAACTGCTGCATTTA**

**GAGACGTACTTGTTGTTTTAAATAAACGAACAAATTAAAATGTCTGATAATGGACCCCAA**

**TCAAACCAACGTAGTGCCCCCCGCATTACATTTGGTGGACCCACAGATTCAACTGACAAT**

**AACCAGAATGGAGGACGCAATGGGGCAAGGCCAAAACAGCGCCGACCCCAAGGTTTACCC**

**AATAATACTGCGTCTTGGTTCACAGCTCTCACTCAGCATGGCAAGGAGGAACTTAGATTC**

**CCTCGAGGCCAGGGCGTTCCAATCAACACCAATAGTGGTCCAGATGACCAAATTGGCTAC**

**TACCGAAGAGCTACCCGACGAGTTCGTGGTGGTGACGGCAAAATGAAAGAGCTCAGCCCC**

**AGATGGTACTTCTATTACCTAGGAACTGGCCCAGAAGCTTCACTTCCCTACGGCGCTAAC**

**AAAGAAGGCATCGTATGGGTTGCAACTGAGGGAGCCTTGAATACACCCAAAGACCACATT**

**GGCACCCGCAATCCTAATAACAATGCTGCCACCGTGCTACAACTTCCTCAAGGAACAACA**

**TTGCCAAAAGGCTTCTACGCAGAGGGAAGCAGAGGCGGCAGTCAAGCCTCTTCTCGCTCC**

**TCATCACGTAGTCGCGGTAATTCAAGAAATTCAACTCCTGGCAGCAGTAGGGGAAATTCT**

**CCTGCTCGAATGGCTAGCGGAGGTGGTGAAACTGCCCTCGCGCTATTGCTGCTAGACAGA**

**TTGAACCAGCTTGAGAGCAAAGTTTCTGGTAAAGGCCAACAACAACAAGGCCAAACTGTC**

**ACTAAGAAATCTGCTGCTGAGGCATCTAAAAAGCCTCGCCAAAAACGTACTGCCACAAAA**

**CAGTACAACGTCACTCAAGCATTTGGGAGACGTGGTCCAGAACAAACCCAAGGAAATTTC**

**GGGGACCAAGACCTAATCAGACAAGGAACTGATTACAAACATTGGCCGCAAATTGCACAA**

**TTTGCTCCAAGTGCCTCTGCATTCTTTGGAATGTCACGCATTGGCATGGAAGTCACACCT**

**TCGGGAACATGGCTGACTTATCATGGAGCCATTAAATTGGATGACAAAGATCCACAATTC**

**AAAGACAACGTCATACTGCTGAACAAGCACATTGACGCATACAAAACATTCCCACCAACA**

**GAGCCTAAAAAGGACAAAAAGAAAAAGACTGATGAAGCTCAGCCTTTGCCGCAGAGACAA**

**AAGAAGCAGCCCACTGTGACTCTTCTTCCTGCGGCTGACATGGATGATTTCTCCAGACAA**

**CTTCAAAATTCCATGAGTGGAGCTTCTGCTGATTCAACTCAGGCATAAACACTCATGATG**

**ACCACACAAGGCAGATGGGCTATGTAAACGTTTTCGCAATTCCGTTTACGATACATAGTC**

**TACTCTTGTGCAGAATGAATTCTCGTAACTAAACAGCACAAGTAGGTTTAGTTAACTTTA**

**ATCTCACATAGCAATCTTTAATCAATGTGTAACATTAGGGAGGACTTGAAAGAGCCACCA**

**CATTTTCATCGAGGCCACGCGGAGTACGATCGAGGGTACAGTGAATAATGCTAGGGAGAG**

**CTGCCTATATGGAAGAGCCCTAATGTGTAAAATTAATTTTAGTAGTGCTATCCCCATGTG**

**ATTTTAATAGCTTCTTAGGAGAATGACAAAAAAAAAAAAAAAAAAAAAAAA**

**SARS-CoV-2**

**ATTAAAGGTTTATACCTTCCCAGGTAACAAACCAACCAACTTTCGATCTCTTGTAGATCTGTTCTCTAAA\**

**CGAACTTTAAAATCTGTGTGGCTGTCACTCGGCTGCATGCTTAGTGCACTCACGCAGTATAATTAATAAC\**

**TAATTACTGTCGTTGACAGGACACGAGTAACTCGTCTATCTTCTGCAGGCTGCTTACGGTTTCGTCCGTG\**

**TTGCAGCCGATCATCAGCACATCTAGGTTTCGTCCGGGTGTGACCGAAAGGTAAGATGGAGAGCCTTGTC\**

**CCTGGTTTCAACGAGAAAACACACGTCCAACTCAGTTTGCCTGTTTTACAGGTTCGCGACGTGCTCGTAC\**

**GTGGCTTTGGAGACTCCGTGGAGGAGGTCTTATCAGAGGCACGTCAACATCTTAAAGATGGCACTTGTGG\**

**CTTAGTAGAAGTTGAAAAAGGCGTTTTGCCTCAACTTGAACAGCCCTATGTGTTCATCAAACGTTCGGAT\**

**GCTCGAACTGCACCTCATGGTCATGTTATGGTTGAGCTGGTAGCAGAACTCGAAGGCATTCAGTACGGTC\**

**GTAGTGGTGAGACACTTGGTGTCCTTGTCCCTCATGTGGGCGAAATACCAGTGGCTTACCGCAAGGTTCT\**

**TCTTCGTAAGAACGGTAATAAAGGAGCTGGTGGCCATAGTTACGGCGCCGATCTAAAGTCATTTGACTTA\**

**GGCGACGAGCTTGGCACTGATCCTTATGAAGATTTTCAAGAAAACTGGAACACTAAACATAGCAGTGGTG\**

**TTACCCGTGAACTCATGCGTGAGCTTAACGGAGGGGCATACACTCGCTATGTCGATAACAACTTCTGTGG\**

**CCCTGATGGCTACCCTCTTGAGTGCATTAAAGACCTTCTAGCACGTGCTGGTAAAGCTTCATGCACTTTG\**

**TCCGAACAACTGGACTTTATTGACACTAAGAGGGGTGTATACTGCTGCCGTGAACATGAGCATGAAATTG\**

**CTTGGTACACGGAACGTTCTGAAAAGAGCTATGAATTGCAGACACCTTTTGAAATTAAATTGGCAAAGAA\**

**ATTTGACACCTTCAATGGGGAATGTCCAAATTTTGTATTTCCCTTAAATTCCATAATCAAGACTATTCAA\**

**CCAAGGGTTGAAAAGAAAAAGCTTGATGGCTTTATGGGTAGAATTCGATCTGTCTATCCAGTTGCGTCAC\**

**CAAATGAATGCAACCAAATGTGCCTTTCAACTCTCATGAAGTGTGATCATTGTGGTGAAACTTCATGGCA\**

**GACGGGCGATTTTGTTAAAGCCACTTGCGAATTTTGTGGCACTGAGAATTTGACTAAAGAAGGTGCCACT\**

**ACTTGTGGTTACTTACCCCAAAATGCTGTTGTTAAAATTTATTGTCCAGCATGTCACAATTCAGAAGTAG\**

**GACCTGAGCATAGTCTTGCCGAATACCATAATGAATCTGGCTTGAAAACCATTCTTCGTAAGGGTGGTCG\**

**CACTATTGCCTTTGGAGGCTGTGTGTTCTCTTATGTTGGTTGCCATAACAAGTGTGCCTATTGGGTTCCA\**

**CGTGCTAGCGCTAACATAGGTTGTAACCATACAGGTGTTGTTGGAGAAGGTTCCGAAGGTCTTAATGACA\**

**ACCTTCTTGAAATACTCCAAAAAGAGAAAGTCAACATCAATATTGTTGGTGACTTTAAACTTAATGAAGA\**

**GATCGCCATTATTTTGGCATCTTTTTCTGCTTCCACAAGTGCTTTTGTGGAAACTGTGAAAGGTTTGGAT\**

**TATAAAGCATTCAAACAAATTGTTGAATCCTGTGGTAATTTTAAAGTTACAAAAGGAAAAGCTAAAAAAG\**

**GTGCCTGGAATATTGGTGAACAGAAATCAATACTGAGTCCTCTTTATGCATTTGCATCAGAGGCTGCTCG\**

**TGTTGTACGATCAATTTTCTCCCGCACTCTTGAAACTGCTCAAAATTCTGTGCGTGTTTTACAGAAGGCC\**

**GCTATAACAATACTAGATGGAATTTCACAGTATTCACTGAGACTCATTGATGCTATGATGTTCACATCTG\**

**ATTTGGCTACTAACAATCTAGTTGTAATGGCCTACATTACAGGTGGTGTTGTTCAGTTGACTTCGCAGTG\**

**GCTAACTAACATCTTTGGCACTGTTTATGAAAAACTCAAACCCGTCCTTGATTGGCTTGAAGAGAAGTTT\**

**AAGGAAGGTGTAGAGTTTCTTAGAGACGGTTGGGAAATTGTTAAATTTATCTCAACCTGTGCTTGTGAAA\**

**TTGTCGGTGGACAAATTGTCACCTGTGCAAAGGAAATTAAGGAGAGTGTTCAGACATTCTTTAAGCTTGT\**

**AAATAAATTTTTGGCTTTGTGTGCTGACTCTATCATTATTGGTGGAGCTAAACTTAAAGCCTTGAATTTA\**

**GGTGAAACATTTGTCACGCACTCAAAGGGATTGTACAGAAAGTGTGTTAAATCCAGAGAAGAAACTGGCC\**

**TACTCATGCCTCTAAAAGCCCCAAAAGAAATTATCTTCTTAGAGGGAGAAACACTTCCCACAGAAGTGTT\**

**AACAGAGGAAGTTGTCTTGAAAACTGGTGATTTACAACCATTAGAACAACCTACTAGTGAAGCTGTTGAA\**

**GCTCCATTGGTTGGTACACCAGTTTGTATTAACGGGCTTATGTTGCTCGAAATCAAAGACACAGAAAAGT\**

**ACTGTGCCCTTGCACCTAATATGATGGTAACAAACAATACCTTCACACTCAAAGGCGGTGCACCAACAAA\**

**GGTTACTTTTGGTGATGACACTGTGATAGAAGTGCAAGGTTACAAGAGTGTGAATATCACTTTTGAACTT\**

**GATGAAAGGATTGATAAAGTACTTAATGAGAAGTGCTCTGCCTATACAGTTGAACTCGGTACAGAAGTAA\**

**ATGAGTTCGCCTGTGTTGTGGCAGATGCTGTCATAAAAACTTTGCAACCAGTATCTGAATTACTTACACC\**

**ACTGGGCATTGATTTAGATGAGTGGAGTATGGCTACATACTACTTATTTGATGAGTCTGGTGAGTTTAAA\**

**TTGGCTTCACATATGTATTGTTCTTTCTACCCTCCAGATGAGGATGAAGAAGAAGGTGATTGTGAAGAAG\**

**AAGAGTTTGAGCCATCAACTCAATATGAGTATGGTACTGAAGATGATTACCAAGGTAAACCTTTGGAATT\**

**TGGTGCCACTTCTGCTGCTCTTCAACCTGAAGAAGAGCAAGAAGAAGATTGGTTAGATGATGATAGTCAA\**

**CAAACTGTTGGTCAACAAGACGGCAGTGAGGACAATCAGACAACTACTATTCAAACAATTGTTGAGGTTC\**

**AACCTCAATTAGAGATGGAACTTACACCAGTTGTTCAGACTATTGAAGTGAATAGTTTTAGTGGTTATTT\**

**AAAACTTACTGACAATGTATACATTAAAAATGCAGACATTGTGGAAGAAGCTAAAAAGGTAAAACCAACA\**

**GTGGTTGTTAATGCAGCCAATGTTTACCTTAAACATGGAGGAGGTGTTGCAGGAGCCTTAAATAAGGCTA\**

**CTAACAATGCCATGCAAGTTGAATCTGATGATTACATAGCTACTAATGGACCACTTAAAGTGGGTGGTAG\**

**TTGTGTTTTAAGCGGACACAATCTTGCTAAACACTGTCTTCATGTTGTCGGCCCAAATGTTAACAAAGGT\**

**GAAGACATTCAACTTCTTAAGAGTGCTTATGAAAATTTTAATCAGCACGAAGTTCTACTTGCACCATTAT\**

**TATCAGCTGGTATTTTTGGTGCTGACCCTATACATTCTTTAAGAGTTTGTGTAGATACTGTTCGCACAAA\**

**TGTCTACTTAGCTGTCTTTGATAAAAATCTCTATGACAAACTTGTTTCAAGCTTTTTGGAAATGAAGAGT\**

**GAAAAGCAAGTTGAACAAAAGATCGCTGAGATTCCTAAAGAGGAAGTTAAGCCATTTATAACTGAAAGTA\**

**AACCTTCAGTTGAACAGAGAAAACAAGATGATAAGAAAATCAAAGCTTGTGTTGAAGAAGTTACAACAAC\**

**TCTGGAAGAAACTAAGTTCCTCACAGAAAACTTGTTACTTTATATTGACATTAATGGCAATCTTCATCCA\**

**GATTCTGCCACTCTTGTTAGTGACATTGACATCACTTTCTTAAAGAAAGATGCTCCATATATAGTGGGTG\**

**ATGTTGTTCAAGAGGGTGTTTTAACTGCTGTGGTTATACCTACTAAAAAGGCTGGTGGCACTACTGAAAT\**

**GCTAGCGAAAGCTTTGAGAAAAGTGCCAACAGACAATTATATAACCACTTACCCGGGTCAGGGTTTAAAT\**

**GGTTACACTGTAGAGGAGGCAAAGACAGTGCTTAAAAAGTGTAAAAGTGCCTTTTACATTCTACCATCTA\**

**TTATCTCTAATGAGAAGCAAGAAATTCTTGGAACTGTTTCTTGGAATTTGCGAGAAATGCTTGCACATGC\**

**AGAAGAAACACGCAAATTAATGCCTGTCTGTGTGGAAACTAAAGCCATAGTTTCAACTATACAGCGTAAA\**

**TATAAGGGTATTAAAATACAAGAGGGTGTGGTTGATTATGGTGCTAGATTTTACTTTTACACCAGTAAAA\**

**CAACTGTAGCGTCACTTATCAACACACTTAACGATCTAAATGAAACTCTTGTTACAATGCCACTTGGCTA\**

**TGTAACACATGGCTTAAATTTGGAAGAAGCTGCTCGGTATATGAGATCTCTCAAAGTGCCAGCTACAGTT\**

**TCTGTTTCTTCACCTGATGCTGTTACAGCGTATAATGGTTATCTTACTTCTTCTTCTAAAACACCTGAAG\**

**AACATTTTATTGAAACCATCTCACTTGCTGGTTCCTATAAAGATTGGTCCTATTCTGGACAATCTACACA\**

**ACTAGGTATAGAATTTCTTAAGAGAGGTGATAAAAGTGTATATTACACTAGTAATCCTACCACATTCCAC\**

**CTAGATGGTGAAGTTATCACCTTTGACAATCTTAAGACACTTCTTTCTTTGAGAGAAGTGAGGACTATTA\**

**AGGTGTTTACAACAGTAGACAACATTAACCTCCACACGCAAGTTGTGGACATGTCAATGACATATGGACA\**

**ACAGTTTGGTCCAACTTATTTGGATGGAGCTGATGTTACTAAAATAAAACCTCATAATTCACATGAAGGT\**

**AAAACATTTTATGTTTTACCTAATGATGACACTCTACGTGTTGAGGCTTTTGAGTACTACCACACAACTG\**

**ATCCTAGTTTTCTGGGTAGGTACATGTCAGCATTAAATCACACTAAAAAGTGGAAATACCCACAAGTTAA\**

**TGGTTTAACTTCTATTAAATGGGCAGATAACAACTGTTATCTTGCCACTGCATTGTTAACACTCCAACAA\**

**ATAGAGTTGAAGTTTAATCCACCTGCTCTACAAGATGCTTATTACAGAGCAAGGGCTGGTGAAGCTGCTA\**

**ACTTTTGTGCACTTATCTTAGCCTACTGTAATAAGACAGTAGGTGAGTTAGGTGATGTTAGAGAAACAAT\**

**GAGTTACTTGTTTCAACATGCCAATTTAGATTCTTGCAAAAGAGTCTTGAACGTGGTGTGTAAAACTTGT\**

**GGACAACAGCAGACAACCCTTAAGGGTGTAGAAGCTGTTATGTACATGGGCACACTTTCTTATGAACAAT\**

**TTAAGAAAGGTGTTCAGATACCTTGTACGTGTGGTAAACAAGCTACAAAATATCTAGTACAACAGGAGTC\**

**ACCTTTTGTTATGATGTCAGCACCACCTGCTCAGTATGAACTTAAGCATGGTACATTTACTTGTGCTAGT\**

**GAGTACACTGGTAATTACCAGTGTGGTCACTATAAACATATAACTTCTAAAGAAACTTTGTATTGCATAG\**

**ACGGTGCTTTACTTACAAAGTCCTCAGAATACAAAGGTCCTATTACGGATGTTTTCTACAAAGAAAACAG\**

**TTACACAACAACCATAAAACCAGTTACTTATAAATTGGATGGTGTTGTTTGTACAGAAATTGACCCTAAG\**

**TTGGACAATTATTATAAGAAAGACAATTCTTATTTCACAGAGCAACCAATTGATCTTGTACCAAACCAAC\**

**CATATCCAAACGCAAGCTTCGATAATTTTAAGTTTGTATGTGATAATATCAAATTTGCTGATGATTTAAA\**

**CCAGTTAACTGGTTATAAGAAACCTGCTTCAAGAGAGCTTAAAGTTACATTTTTCCCTGACTTAAATGGT\**

**GATGTGGTGGCTATTGATTATAAACACTACACACCCTCTTTTAAGAAAGGAGCTAAATTGTTACATAAAC\**

**CTATTGTTTGGCATGTTAACAATGCAACTAATAAAGCCACGTATAAACCAAATACCTGGTGTATACGTTG\**

**TCTTTGGAGCACAAAACCAGTTGAAACATCAAATTCGTTTGATGTACTGAAGTCAGAGGACGCGCAGGGA\**

**ATGGATAATCTTGCCTGCGAAGATCTAAAACCAGTCTCTGAAGAAGTAGTGGAAAATCCTACCATACAGA\**

**AAGACGTTCTTGAGTGTAATGTGAAAACTACCGAAGTTGTAGGAGACATTATACTTAAACCAGCAAATAA\**

**TAGTTTAAAAATTACAGAAGAGGTTGGCCACACAGATCTAATGGCTGCTTATGTAGACAATTCTAGTCTT\**

**ACTATTAAGAAACCTAATGAATTATCTAGAGTATTAGGTTTGAAAACCCTTGCTACTCATGGTTTAGCTG\**

**CTGTTAATAGTGTCCCTTGGGATACTATAGCTAATTATGCTAAGCCTTTTCTTAACAAAGTTGTTAGTAC\**

**AACTACTAACATAGTTACACGGTGTTTAAACCGTGTTTGTACTAATTATATGCCTTATTTCTTTACTTTA\**

**TTGCTACAATTGTGTACTTTTACTAGAAGTACAAATTCTAGAATTAAAGCATCTATGCCGACTACTATAG\**

**CAAAGAATACTGTTAAGAGTGTCGGTAAATTTTGTCTAGAGGCTTCATTTAATTATTTGAAGTCACCTAA\**

**TTTTTCTAAACTGATAAATATTATAATTTGGTTTTTACTATTAAGTGTTTGCCTAGGTTCTTTAATCTAC\**

**TCAACCGCTGCTTTAGGTGTTTTAATGTCTAATTTAGGCATGCCTTCTTACTGTACTGGTTACAGAGAAG\**

**GCTATTTGAACTCTACTAATGTCACTATTGCAACCTACTGTACTGGTTCTATACCTTGTAGTGTTTGTCT\**

**TAGTGGTTTAGATTCTTTAGACACCTATCCTTCTTTAGAAACTATACAAATTACCATTTCATCTTTTAAA\**

**TGGGATTTAACTGCTTTTGGCTTAGTTGCAGAGTGGTTTTTGGCATATATTCTTTTCACTAGGTTTTTCT\**

**ATGTACTTGGATTGGCTGCAATCATGCAATTGTTTTTCAGCTATTTTGCAGTACATTTTATTAGTAATTC\**

**TTGGCTTATGTGGTTAATAATTAATCTTGTACAAATGGCCCCGATTTCAGCTATGGTTAGAATGTACATC\**

**TTCTTTGCATCATTTTATTATGTATGGAAAAGTTATGTGCATGTTGTAGACGGTTGTAATTCATCAACTT\**

**GTATGATGTGTTACAAACGTAATAGAGCAACAAGAGTCGAATGTACAACTATTGTTAATGGTGTTAGAAG\**

**GTCCTTTTATGTCTATGCTAATGGAGGTAAAGGCTTTTGCAAACTACACAATTGGAATTGTGTTAATTGT\**

**GATACATTCTGTGCTGGTAGTACATTTATTAGTGATGAAGTTGCGAGAGACTTGTCACTACAGTTTAAAA\**

**GACCAATAAATCCTACTGACCAGTCTTCTTACATCGTTGATAGTGTTACAGTGAAGAATGGTTCCATCCA\**

**TCTTTACTTTGATAAAGCTGGTCAAAAGACTTATGAAAGACATTCTCTCTCTCATTTTGTTAACTTAGAC\**

**AACCTGAGAGCTAATAACACTAAAGGTTCATTGCCTATTAATGTTATAGTTTTTGATGGTAAATCAAAAT\**

**GTGAAGAATCATCTGCAAAATCAGCGTCTGTTTACTACAGTCAGCTTATGTGTCAACCTATACTGTTACT\**

**AGATCAGGCATTAGTGTCTGATGTTGGTGATAGTGCGGAAGTTGCAGTTAAAATGTTTGATGCTTACGTT\**

**AATACGTTTTCATCAACTTTTAACGTACCAATGGAAAAACTCAAAACACTAGTTGCAACTGCAGAAGCTG\**

**AACTTGCAAAGAATGTGTCCTTAGACAATGTCTTATCTACTTTTATTTCAGCAGCTCGGCAAGGGTTTGT\**

**TGATTCAGATGTAGAAACTAAAGATGTTGTTGAATGTCTTAAATTGTCACATCAATCTGACATAGAAGTT\**

**ACTGGCGATAGTTGTAATAACTATATGCTCACCTATAACAAAGTTGAAAACATGACACCCCGTGACCTTG\**

**GTGCTTGTATTGACTGTAGTGCGCGTCATATTAATGCGCAGGTAGCAAAAAGTCACAACATTGCTTTGAT\**

**ATGGAACGTTAAAGATTTCATGTCATTGTCTGAACAACTACGAAAACAAATACGTAGTGCTGCTAAAAAG\**

**AATAACTTACCTTTTAAGTTGACATGTGCAACTACTAGACAAGTTGTTAATGTTGTAACAACAAAGATAG\**

**CACTTAAGGGTGGTAAAATTGTTAATAATTGGTTGAAGCAGTTAATTAAAGTTACACTTGTGTTCCTTTT\**

**TGTTGCTGCTATTTTCTATTTAATAACACCTGTTCATGTCATGTCTAAACATACTGACTTTTCAAGTGAA\**

**ATCATAGGATACAAGGCTATTGATGGTGGTGTCACTCGTGACATAGCATCTACAGATACTTGTTTTGCTA\**

**ACAAACATGCTGATTTTGACACATGGTTTAGCCAGCGTGGTGGTAGTTATACTAATGACAAAGCTTGCCC\**

**ATTGATTGCTGCAGTCATAACAAGAGAAGTGGGTTTTGTCGTGCCTGGTTTGCCTGGCACGATATTACGC\**

**ACAACTAATGGTGACTTTTTGCATTTCTTACCTAGAGTTTTTAGTGCAGTTGGTAACATCTGTTACACAC\**

**CATCAAAACTTATAGAGTACACTGACTTTGCAACATCAGCTTGTGTTTTGGCTGCTGAATGTACAATTTT\**

**TAAAGATGCTTCTGGTAAGCCAGTACCATATTGTTATGATACCAATGTACTAGAAGGTTCTGTTGCTTAT\**

**GAAAGTTTACGCCCTGACACACGTTATGTGCTCATGGATGGCTCTATTATTCAATTTCCTAACACCTACC\**

**TTGAAGGTTCTGTTAGAGTGGTAACAACTTTTGATTCTGAGTACTGTAGGCACGGCACTTGTGAAAGATC\**

**AGAAGCTGGTGTTTGTGTATCTACTAGTGGTAGATGGGTACTTAACAATGATTATTACAGATCTTTACCA\**

**GGAGTTTTCTGTGGTGTAGATGCTGTAAATTTACTTACTAATATGTTTACACCACTAATTCAACCTATTG\**

**GTGCTTTGGACATATCAGCATCTATAGTAGCTGGTGGTATTGTAGCTATCGTAGTAACATGCCTTGCCTA\**

**CTATTTTATGAGGTTTAGAAGAGCTTTTGGTGAATACAGTCATGTAGTTGCCTTTAATACTTTACTATTC\**

**CTTATGTCATTCACTGTACTCTGTTTAACACCAGTTTACTCATTCTTACCTGGTGTTTATTCTGTTATTT\**

**ACTTGTACTTGACATTTTATCTTACTAATGATGTTTCTTTTTTAGCACATATTCAGTGGATGGTTATGTT\**

**CACACCTTTAGTACCTTTCTGGATAACAATTGCTTATATCATTTGTATTTCCACAAAGCATTTCTATTGG\**

**TTCTTTAGTAATTACCTAAAGAGACGTGTAGTCTTTAATGGTGTTTCCTTTAGTACTTTTGAAGAAGCTG\**

**CGCTGTGCACCTTTTTGTTAAATAAAGAAATGTATCTAAAGTTGCGTAGTGATGTGCTATTACCTCTTAC\**

**GCAATATAATAGATACTTAGCTCTTTATAATAAGTACAAGTATTTTAGTGGAGCAATGGATACAACTAGC\**

**TACAGAGAAGCTGCTTGTTGTCATCTCGCAAAGGCTCTCAATGACTTCAGTAACTCAGGTTCTGATGTTC\**

**TTTACCAACCACCACAAACCTCTATCACCTCAGCTGTTTTGCAGAGTGGTTTTAGAAAAATGGCATTCCC\**

**ATCTGGTAAAGTTGAGGGTTGTATGGTACAAGTAACTTGTGGTACAACTACACTTAACGGTCTTTGGCTT\**

**GATGACGTAGTTTACTGTCCAAGACATGTGATCTGCACCTCTGAAGACATGCTTAACCCTAATTATGAAG\**

**ATTTACTCATTCGTAAGTCTAATCATAATTTCTTGGTACAGGCTGGTAATGTTCAACTCAGGGTTATTGG\**

**ACATTCTATGCAAAATTGTGTACTTAAGCTTAAGGTTGATACAGCCAATCCTAAGACACCTAAGTATAAG\**

**TTTGTTCGCATTCAACCAGGACAGACTTTTTCAGTGTTAGCTTGTTACAATGGTTCACCATCTGGTGTTT\**

**ACCAATGTGCTATGAGGCCCAATTTCACTATTAAGGGTTCATTCCTTAATGGTTCATGTGGTAGTGTTGG\**

**TTTTAACATAGATTATGACTGTGTCTCTTTTTGTTACATGCACCATATGGAATTACCAACTGGAGTTCAT\**

**GCTGGCACAGACTTAGAAGGTAACTTTTATGGACCTTTTGTTGACAGGCAAACAGCACAAGCAGCTGGTA\**

**CGGACACAACTATTACAGTTAATGTTTTAGCTTGGTTGTACGCTGCTGTTATAAATGGAGACAGGTGGTT\**

**TCTCAATCGATTTACCACAACTCTTAATGACTTTAACCTTGTGGCTATGAAGTACAATTATGAACCTCTA\**

**ACACAAGACCATGTTGACATACTAGGACCTCTTTCTGCTCAAACTGGAATTGCCGTTTTAGATATGTGTG\**

**CTTCATTAAAAGAATTACTGCAAAATGGTATGAATGGACGTACCATATTGGGTAGTGCTTTATTAGAAGA\**

**TGAATTTACACCTTTTGATGTTGTTAGACAATGCTCAGGTGTTACTTTCCAAAGTGCAGTGAAAAGAACA\**

**ATCAAGGGTACACACCACTGGTTGTTACTCACAATTTTGACTTCACTTTTAGTTTTAGTCCAGAGTACTC\**

**AATGGTCTTTGTTCTTTTTTTTGTATGAAAATGCCTTTTTACCTTTTGCTATGGGTATTATTGCTATGTC\**

**TGCTTTTGCAATGATGTTTGTCAAACATAAGCATGCATTTCTCTGTTTGTTTTTGTTACCTTCTCTTGCC\**

**ACTGTAGCTTATTTTAATATGGTCTATATGCCTGCTAGTTGGGTGATGCGTATTATGACATGGTTGGATA\**

**TGGTTGATACTAGTTTGTCTGGTTTTAAGCTAAAAGACTGTGTTATGTATGCATCAGCTGTAGTGTTACT\**

**AATCCTTATGACAGCAAGAACTGTGTATGATGATGGTGCTAGGAGAGTGTGGACACTTATGAATGTCTTG\**

**ACACTCGTTTATAAAGTTTATTATGGTAATGCTTTAGATCAAGCCATTTCCATGTGGGCTCTTATAATCT\**

**CTGTTACTTCTAACTACTCAGGTGTAGTTACAACTGTCATGTTTTTGGCCAGAGGTATTGTTTTTATGTG\**

**TGTTGAGTATTGCCCTATTTTCTTCATAACTGGTAATACACTTCAGTGTATAATGCTAGTTTATTGTTTC\**

**TTAGGCTATTTTTGTACTTGTTACTTTGGCCTCTTTTGTTTACTCAACCGCTACTTTAGACTGACTCTTG\**

**GTGTTTATGATTACTTAGTTTCTACACAGGAGTTTAGATATATGAATTCACAGGGACTACTCCCACCCAA\**

**GAATAGCATAGATGCCTTCAAACTCAACATTAAATTGTTGGGTGTTGGTGGCAAACCTTGTATCAAAGTA\**

**GCCACTGTACAGTCTAAAATGTCAGATGTAAAGTGCACATCAGTAGTCTTACTCTCAGTTTTGCAACAAC\**

**TCAGAGTAGAATCATCATCTAAATTGTGGGCTCAATGTGTCCAGTTACACAATGACATTCTCTTAGCTAA\**

**AGATACTACTGAAGCCTTTGAAAAAATGGTTTCACTACTTTCTGTTTTGCTTTCCATGCAGGGTGCTGTA\**

**GACATAAACAAGCTTTGTGAAGAAATGCTGGACAACAGGGCAACCTTACAAGCTATAGCCTCAGAGTTTA\**

**GTTCCCTTCCATCATATGCAGCTTTTGCTACTGCTCAAGAAGCTTATGAGCAGGCTGTTGCTAATGGTGA\**

**TTCTGAAGTTGTTCTTAAAAAGTTGAAGAAGTCTTTGAATGTGGCTAAATCTGAATTTGACCGTGATGCA\**

**GCCATGCAACGTAAGTTGGAAAAGATGGCTGATCAAGCTATGACCCAAATGTATAAACAGGCTAGATCTG\**

**AGGACAAGAGGGCAAAAGTTACTAGTGCTATGCAGACAATGCTTTTCACTATGCTTAGAAAGTTGGATAA\**

**TGATGCACTCAACAACATTATCAACAATGCAAGAGATGGTTGTGTTCCCTTGAACATAATACCTCTTACA\**

**ACAGCAGCCAAACTAATGGTTGTCATACCAGACTATAACACATATAAAAATACGTGTGATGGTACAACAT\**

**TTACTTATGCATCAGCATTGTGGGAAATCCAACAGGTTGTAGATGCAGATAGTAAAATTGTTCAACTTAG\**

**TGAAATTAGTATGGACAATTCACCTAATTTAGCATGGCCTCTTATTGTAACAGCTTTAAGGGCCAATTCT\**

**GCTGTCAAATTACAGAATAATGAGCTTAGTCCTGTTGCACTACGACAGATGTCTTGTGCTGCCGGTACTA\**

**CACAAACTGCTTGCACTGATGACAATGCGTTAGCTTACTACAACACAACAAAGGGAGGTAGGTTTGTACT\**

**TGCACTGTTATCCGATTTACAGGATTTGAAATGGGCTAGATTCCCTAAGAGTGATGGAACTGGTACTATC\**

**TATACAGAACTGGAACCACCTTGTAGGTTTGTTACAGACACACCTAAAGGTCCTAAAGTGAAGTATTTAT\**

**ACTTTATTAAAGGATTAAACAACCTAAATAGAGGTATGGTACTTGGTAGTTTAGCTGCCACAGTACGTCT\**

**ACAAGCTGGTAATGCAACAGAAGTGCCTGCCAATTCAACTGTATTATCTTTCTGTGCTTTTGCTGTAGAT\**

**GCTGCTAAAGCTTACAAAGATTATCTAGCTAGTGGGGGACAACCAATCACTAATTGTGTTAAGATGTTGT\**

**GTACACACACTGGTACTGGTCAGGCAATAACAGTTACACCGGAAGCCAATATGGATCAAGAATCCTTTGG\**

**TGGTGCATCGTGTTGTCTGTACTGCCGTTGCCACATAGATCATCCAAATCCTAAAGGATTTTGTGACTTA\**

**AAAGGTAAGTATGTACAAATACCTACAACTTGTGCTAATGACCCTGTGGGTTTTACACTTAAAAACACAG\**

**TCTGTACCGTCTGCGGTATGTGGAAAGGTTATGGCTGTAGTTGTGATCAACTCCGCGAACCCATGCTTCA\**

**GTCAGCTGATGCACAATCGTTTTTAAACGGGTTTGCGGTGTAAGTGCAGCCCGTCTTACACCGTGCGGCA\**

**CAGGCACTAGTACTGATGTCGTATACAGGGCTTTTGACATCTACAATGATAAAGTAGCTGGTTTTGCTAA\**

**ATTCCTAAAAACTAATTGTTGTCGCTTCCAAGAAAAGGACGAAGATGACAATTTAATTGATTCTTACTTT\**

**GTAGTTAAGAGACACACTTTCTCTAACTACCAACATGAAGAAACAATTTATAATTTACTTAAGGATTGTC\**

**CAGCTGTTGCTAAACATGACTTCTTTAAGTTTAGAATAGACGGTGACATGGTACCACATATATCACGTCA\**

**ACGTCTTACTAAATACACAATGGCAGACCTCGTCTATGCTTTAAGGCATTTTGATGAAGGTAATTGTGAC\**

**ACATTAAAAGAAATACTTGTCACATACAATTGTTGTGATGATGATTATTTCAATAAAAAGGACTGGTATG\**

**ATTTTGTAGAAAACCCAGATATATTACGCGTATACGCCAACTTAGGTGAACGTGTACGCCAAGCTTTGTT\**

**AAAAACAGTACAATTCTGTGATGCCATGCGAAATGCTGGTATTGTTGGTGTACTGACATTAGATAATCAA\**

**GATCTCAATGGTAACTGGTATGATTTCGGTGATTTCATACAAACCACGCCAGGTAGTGGAGTTCCTGTTG\**

**TAGATTCTTATTATTCATTGTTAATGCCTATATTAACCTTGACCAGGGCTTTAACTGCAGAGTCACATGT\**

**TGACACTGACTTAACAAAGCCTTACATTAAGTGGGATTTGTTAAAATATGACTTCACGGAAGAGAGGTTA\**

**AAACTCTTTGACCGTTATTTTAAATATTGGGATCAGACATACCACCCAAATTGTGTTAACTGTTTGGATG\**

**ACAGATGCATTCTGCATTGTGCAAACTTTAATGTTTTATTCTCTACAGTGTTCCCACCTACAAGTTTTGG\**

**ACCACTAGTGAGAAAAATATTTGTTGATGGTGTTCCATTTGTAGTTTCAACTGGATACCACTTCAGAGAG\**

**CTAGGTGTTGTACATAATCAGGATGTAAACTTACATAGCTCTAGACTTAGTTTTAAGGAATTACTTGTGT\**

**ATGCTGCTGACCCTGCTATGCACGCTGCTTCTGGTAATCTATTACTAGATAAACGCACTACGTGCTTTTC\**

**AGTAGCTGCACTTACTAACAATGTTGCTTTTCAAACTGTCAAACCCGGTAATTTTAACAAAGACTTCTAT\**

**GACTTTGCTGTGTCTAAGGGTTTCTTTAAGGAAGGAAGTTCTGTTGAATTAAAACACTTCTTCTTTGCTC\**

**AGGATGGTAATGCTGCTATCAGCGATTATGACTACTATCGTTATAATCTACCAACAATGTGTGATATCAG\**

**ACAACTACTATTTGTAGTTGAAGTTGTTGATAAGTACTTTGATTGTTACGATGGTGGCTGTATTAATGCT\**

**AACCAAGTCATCGTCAACAACCTAGACAAATCAGCTGGTTTTCCATTTAATAAATGGGGTAAGGCTAGAC\**

**TTTATTATGATTCAATGAGTTATGAGGATCAAGATGCACTTTTCGCATATACAAAACGTAATGTCATCCC\**

**TACTATAACTCAAATGAATCTTAAGTATGCCATTAGTGCAAAGAATAGAGCTCGCACCGTAGCTGGTGTC\**

**TCTATCTGTAGTACTATGACCAATAGACAGTTTCATCAAAAATTATTGAAATCAATAGCCGCCACTAGAG\**

**GAGCTACTGTAGTAATTGGAACAAGCAAATTCTATGGTGGTTGGCACAACATGTTAAAAACTGTTTATAG\**

**TGATGTAGAAAACCCTCACCTTATGGGTTGGGATTATCCTAAATGTGATAGAGCCATGCCTAACATGCTT\**

**AGAATTATGGCCTCACTTGTTCTTGCTCGCAAACATACAACGTGTTGTAGCTTGTCACACCGTTTCTATA\**

**GATTAGCTAATGAGTGTGCTCAAGTATTGAGTGAAATGGTCATGTGTGGCGGTTCACTATATGTTAAACC\**

**AGGTGGAACCTCATCAGGAGATGCCACAACTGCTTATGCTAATAGTGTTTTTAACATTTGTCAAGCTGTC\**

**ACGGCCAATGTTAATGCACTTTTATCTACTGATGGTAACAAAATTGCCGATAAGTATGTCCGCAATTTAC\**

**AACACAGACTTTATGAGTGTCTCTATAGAAATAGAGATGTTGACACAGACTTTGTGAATGAGTTTTACGC\**

**ATATTTGCGTAAACATTTCTCAATGATGATACTCTCTGACGATGCTGTTGTGTGTTTCAATAGCACTTAT\**

**GCATCTCAAGGTCTAGTGGCTAGCATAAAGAACTTTAAGTCAGTTCTTTATTATCAAAACAATGTTTTTA\**

**TGTCTGAAGCAAAATGTTGGACTGAGACTGACCTTACTAAAGGACCTCATGAATTTTGCTCTCAACATAC\**

**AATGCTAGTTAAACAGGGTGATGATTATGTGTACCTTCCTTACCCAGATCCATCAAGAATCCTAGGGGCC\**

**GGCTGTTTTGTAGATGATATCGTAAAAACAGATGGTACACTTATGATTGAACGGTTCGTGTCTTTAGCTA\**

**TAGATGCTTACCCACTTACTAAACATCCTAATCAGGAGTATGCTGATGTCTTTCATTTGTACTTACAATA\**

**CATAAGAAAGCTACATGATGAGTTAACAGGACACATGTTAGACATGTATTCTGTTATGCTTACTAATGAT\**

**AACACTTCAAGGTATTGGGAACCTGAGTTTTATGAGGCTATGTACACACCGCATACAGTCTTACAGGCTG\**

**TTGGGGCTTGTGTTCTTTGCAATTCACAGACTTCATTAAGATGTGGTGCTTGCATACGTAGACCATTCTT\**

**ATGTTGTAAATGCTGTTACGACCATGTCATATCAACATCACATAAATTAGTCTTGTCTGTTAATCCGTAT\**

**GTTTGCAATGCTCCAGGTTGTGATGTCACAGATGTGACTCAACTTTACTTAGGAGGTATGAGCTATTATT\**

**GTAAATCACATAAACCACCCATTAGTTTTCCATTGTGTGCTAATGGACAAGTTTTTGGTTTATATAAAAA\**

**TACATGTGTTGGTAGCGATAATGTTACTGACTTTAATGCAATTGCAACATGTGACTGGACAAATGCTGGT\**

**GATTACATTTTAGCTAACACCTGTACTGAAAGACTCAAGCTTTTTGCAGCAGAAACGCTCAAAGCTACTG\**

**AGGAGACATTTAAACTGTCTTATGGTATTGCTACTGTACGTGAAGTGCTGTCTGACAGAGAATTACATCT\**

**TTCATGGGAAGTTGGTAAACCTAGACCACCACTTAACCGAAATTATGTCTTTACTGGTTATCGTGTAACT\**

**AAAAACAGTAAAGTACAAATAGGAGAGTACACCTTTGAAAAAGGTGACTATGGTGATGCTGTTGTTTACC\**

**GAGGTACAACAACTTACAAATTAAATGTTGGTGATTATTTTGTGCTGACATCACATACAGTAATGCCATT\**

**AAGTGCACCTACACTAGTGCCACAAGAGCACTATGTTAGAATTACTGGCTTATACCCAACACTCAATATC\**

**TCAGATGAGTTTTCTAGCAATGTTGCAAATTATCAAAAGGTTGGTATGCAAAAGTATTCTACACTCCAGG\**

**GACCACCTGGTACTGGTAAGAGTCATTTTGCTATTGGCCTAGCTCTCTACTACCCTTCTGCTCGCATAGT\**

**GTATACAGCTTGCTCTCATGCCGCTGTTGATGCACTATGTGAGAAGGCATTAAAATATTTGCCTATAGAT\**

**AAATGTAGTAGAATTATACCTGCACGTGCTCGTGTAGAGTGTTTTGATAAATTCAAAGTGAATTCAACAT\**

**TAGAACAGTATGTCTTTTGTACTGTAAATGCATTGCCTGAGACGACAGCAGATATAGTTGTCTTTGATGA\**

**AATTTCAATGGCCACAAATTATGATTTGAGTGTTGTCAATGCCAGATTACGTGCTAAGCACTATGTGTAC\**

**ATTGGCGACCCTGCTCAATTACCTGCACCACGCACATTGCTAACTAAGGGCACACTAGAACCAGAATATT\**

**TCAATTCAGTGTGTAGACTTATGAAAACTATAGGTCCAGACATGTTCCTCGGAACTTGTCGGCGTTGTCC\**

**TGCTGAAATTGTTGACACTGTGAGTGCTTTGGTTTATGATAATAAGCTTAAAGCACATAAAGACAAATCA\**

**GCTCAATGCTTTAAAATGTTTTATAAGGGTGTTATCACGCATGATGTTTCATCTGCAATTAACAGGCCAC\**

**AAATAGGCGTGGTAAGAGAATTCCTTACACGTAACCCTGCTTGGAGAAAAGCTGTCTTTATTTCACCTTA\**

**TAATTCACAGAATGCTGTAGCCTCAAAGATTTTGGGACTACCAACTCAAACTGTTGATTCATCACAGGGC\**

**TCAGAATATGACTATGTCATATTCACTCAAACCACTGAAACAGCTCACTCTTGTAATGTAAACAGATTTA\**

**ATGTTGCTATTACCAGAGCAAAAGTAGGCATACTTTGCATAATGTCTGATAGAGACCTTTATGACAAGTT\**

**GCAATTTACAAGTCTTGAAATTCCACGTAGGAATGTGGCAACTTTACAAGCTGAAAATGTAACAGGACTC\**

**TTTAAAGATTGTAGTAAGGTAATCACTGGGTTACATCCTACACAGGCACCTACACACCTCAGTGTTGACA\**

**CTAAATTCAAAACTGAAGGTTTATGTGTTGACATACCTGGCATACCTAAGGACATGACCTATAGAAGACT\**

**CATCTCTATGATGGGTTTTAAAATGAATTATCAAGTTAATGGTTACCCTAACATGTTTATCACCCGCGAA\**

**GAAGCTATAAGACATGTACGTGCATGGATTGGCTTCGATGTCGAGGGGTGTCATGCTACTAGAGAAGCTG\**

**TTGGTACCAATTTACCTTTACAGCTAGGTTTTTCTACAGGTGTTAACCTAGTTGCTGTACCTACAGGTTA\**

**TGTTGATACACCTAATAATACAGATTTTTCCAGAGTTAGTGCTAAACCACCGCCTGGAGATCAATTTAAA\**

**CACCTCATACCACTTATGTACAAAGGACTTCCTTGGAATGTAGTGCGTATAAAGATTGTACAAATGTTAA\**

**GTGACACACTTAAAAATCTCTCTGACAGAGTCGTATTTGTCTTATGGGCACATGGCTTTGAGTTGACATC\**

**TATGAAGTATTTTGTGAAAATAGGACCTGAGCGCACCTGTTGTCTATGTGATAGACGTGCCACATGCTTT\**

**TCCACTGCTTCAGACACTTATGCCTGTTGGCATCATTCTATTGGATTTGATTACGTCTATAATCCGTTTA\**

**TGATTGATGTTCAACAATGGGGTTTTACAGGTAACCTACAAAGCAACCATGATCTGTATTGTCAAGTCCA\**

**TGGTAATGCACATGTAGCTAGTTGTGATGCAATCATGACTAGGTGTCTAGCTGTCCACGAGTGCTTTGTT\**

**AAGCGTGTTGACTGGACTATTGAATATCCTATAATTGGTGATGAACTGAAGATTAATGCGGCTTGTAGAA\**

**AGGTTCAACACATGGTTGTTAAAGCTGCATTATTAGCAGACAAATTCCCAGTTCTTCACGACATTGGTAA\**

**CCCTAAAGCTATTAAGTGTGTACCTCAAGCTGATGTAGAATGGAAGTTCTATGATGCACAGCCTTGTAGT\**

**GACAAAGCTTATAAAATAGAAGAATTATTCTATTCTTATGCCACACATTCTGACAAATTCACAGATGGTG\**

**TATGCCTATTTTGGAATTGCAATGTCGATAGATATCCTGCTAATTCCATTGTTTGTAGATTTGACACTAG\**

**AGTGCTATCTAACCTTAACTTGCCTGGTTGTGATGGTGGCAGTTTGTATGTAAATAAACATGCATTCCAC\**

**ACACCAGCTTTTGATAAAAGTGCTTTTGTTAATTTAAAACAATTACCATTTTTCTATTACTCTGACAGTC\**

**CATGTGAGTCTCATGGAAAACAAGTAGTGTCAGATATAGATTATGTACCACTAAAGTCTGCTACGTGTAT\**

**AACACGTTGCAATTTAGGTGGTGCTGTCTGTAGACATCATGCTAATGAGTACAGATTGTATCTCGATGCT\**

**TATAACATGATGATCTCAGCTGGCTTTAGCTTGTGGGTTTACAAACAATTTGATACTTATAACCTCTGGA\**

**ACACTTTTACAAGACTTCAGAGTTTAGAAAATGTGGCTTTTAATGTTGTAAATAAGGGACACTTTGATGG\**

**ACAACAGGGTGAAGTACCAGTTTCTATCATTAATAACACTGTTTACACAAAAGTTGATGGTGTTGATGTA\**

**GAATTGTTTGAAAATAAAACAACATTACCTGTTAATGTAGCATTTGAGCTTTGGGCTAAGCGCAACATTA\**

**AACCAGTACCAGAGGTGAAAATACTCAATAATTTGGGTGTGGACATTGCTGCTAATACTGTGATCTGGGA\**

**CTACAAAAGAGATGCTCCAGCACATATATCTACTATTGGTGTTTGTTCTATGACTGACATAGCCAAGAAA\**

**CCAACTGAAACGATTTGTGCACCACTCACTGTCTTTTTTGATGGTAGAGTTGATGGTCAAGTAGACTTAT\**

**TTAGAAATGCCCGTAATGGTGTTCTTATTACAGAAGGTAGTGTTAAAGGTTTACAACCATCTGTAGGTCC\**

**CAAACAAGCTAGTCTTAATGGAGTCACATTAATTGGAGAAGCCGTAAAAACACAGTTCAATTATTATAAG\**

**AAAGTTGATGGTGTTGTCCAACAATTACCTGAAACTTACTTTACTCAGAGTAGAAATTTACAAGAATTTA\**

**AACCCAGGAGTCAAATGGAAATTGATTTCTTAGAATTAGCTATGGATGAATTCATTGAACGGTATAAATT\**

**AGAAGGCTATGCCTTCGAACATATCGTTTATGGAGATTTTAGTCATAGTCAGTTAGGTGGTTTACATCTA\**

**CTGATTGGACTAGCTAAACGTTTTAAGGAATCACCTTTTGAATTAGAAGATTTTATTCCTATGGACAGTA\**

**CAGTTAAAAACTATTTCATAACAGATGCGCAAACAGGTTCATCTAAGTGTGTGTGTTCTGTTATTGATTT\**

**ATTACTTGATGATTTTGTTGAAATAATAAAATCCCAAGATTTATCTGTAGTTTCTAAGGTTGTCAAAGTG\**

**ACTATTGACTATACAGAAATTTCATTTATGCTTTGGTGTAAAGATGGCCATGTAGAAACATTTTACCCAA\**

**AATTACAATCTAGTCAAGCGTGGCAACCGGGTGTTGCTATGCCTAATCTTTACAAAATGCAAAGAATGCT\**

**ATTAGAAAAGTGTGACCTTCAAAATTATGGTGATAGTGCAACATTACCTAAAGGCATAATGATGAATGTC\**

**GCAAAATATACTCAACTGTGTCAATATTTAAACACATTAACATTAGCTGTACCCTATAATATGAGAGTTA\**

**TACATTTTGGTGCTGGTTCTGATAAAGGAGTTGCACCAGGTACAGCTGTTTTAAGACAGTGGTTGCCTAC\**

**GGGTACGCTGCTTGTCGATTCAGATCTTAATGACTTTGTCTCTGATGCAGATTCAACTTTGATTGGTGAT\**

**TGTGCAACTGTACATACAGCTAATAAATGGGATCTCATTATTAGTGATATGTACGACCCTAAGACTAAAA\**

**ATGTTACAAAAGAAAATGACTCTAAAGAGGGTTTTTTCACTTACATTTGTGGGTTTATACAACAAAAGCT\**

**AGCTCTTGGAGGTTCCGTGGCTATAAAGATAACAGAACATTCTTGGAATGCTGATCTTTATAAGCTCATG\**

**GGACACTTCGCATGGTGGACAGCCTTTGTTACTAATGTGAATGCGTCATCATCTGAAGCATTTTTAATTG\**

**GATGTAATTATCTTGGCAAACCACGCGAACAAATAGATGGTTATGTCATGCATGCAAATTACATATTTTG\**

**GAGGAATACAAATCCAATTCAGTTGTCTTCCTATTCTTTATTTGACATGAGTAAATTTCCCCTTAAATTA\**

**AGGGGTACTGCTGTTATGTCTTTAAAAGAAGGTCAAATCAATGATATGATTTTATCTCTTCTTAGTAAAG\**

**GTAGACTTATAATTAGAGAAAACAACAGAGTTGTTATTTCTAGTGATGTTCTTGTTAACAACTAAACGAA\**

**CAATGTTTGTTTTTCTTGTTTTATTGCCACTAGTCTCTAGTCAGTGTGTTAATCTTACAACCAGAACTCA\**

**ATTACCCCCTGCATACACTAATTCTTTCACACGTGGTGTTTATTACCCTGACAAAGTTTTCAGATCCTCA\**

**GTTTTACATTCAACTCAGGACTTGTTCTTACCTTTCTTTTCCAATGTTACTTGGTTCCATGCTATACATG\**

**TCTCTGGGACCAATGGTACTAAGAGGTTTGATAACCCTGTCCTACCATTTAATGATGGTGTTTATTTTGC\**

**TTCCACTGAGAAGTCTAACATAATAAGAGGCTGGATTTTTGGTACTACTTTAGATTCGAAGACCCAGTCC\**

**CTACTTATTGTTAATAACGCTACTAATGTTGTTATTAAAGTCTGTGAATTTCAATTTTGTAATGATCCAT\**

**TTTTGGGTGTTTATTACCACAAAAACAACAAAAGTTGGATGGAAAGTGAGTTCAGAGTTTATTCTAGTGC\**

**GAATAATTGCACTTTTGAATATGTCTCTCAGCCTTTTCTTATGGACCTTGAAGGAAAACAGGGTAATTTC\**

**AAAAATCTTAGGGAATTTGTGTTTAAGAATATTGATGGTTATTTTAAAATATATTCTAAGCACACGCCTA\**

**TTAATTTAGTGCGTGATCTCCCTCAGGGTTTTTCGGCTTTAGAACCATTGGTAGATTTGCCAATAGGTAT\**

**TAACATCACTAGGTTTCAAACTTTACTTGCTTTACATAGAAGTTATTTGACTCCTGGTGATTCTTCTTCA\**

**GGTTGGACAGCTGGTGCTGCAGCTTATTATGTGGGTTATCTTCAACCTAGGACTTTTCTATTAAAATATA\**

**ATGAAAATGGAACCATTACAGATGCTGTAGACTGTGCACTTGACCCTCTCTCAGAAACAAAGTGTACGTT\**

**GAAATCCTTCACTGTAGAAAAAGGAATCTATCAAACTTCTAACTTTAGAGTCCAACCAACAGAATCTATT\**

**GTTAGATTTCCTAATATTACAAACTTGTGCCCTTTTGGTGAAGTTTTTAACGCCACCAGATTTGCATCTG\**

**TTTATGCTTGGAACAGGAAGAGAATCAGCAACTGTGTTGCTGATTATTCTGTCCTATATAATTCCGCATC\**

**ATTTTCCACTTTTAAGTGTTATGGAGTGTCTCCTACTAAATTAAATGATCTCTGCTTTACTAATGTCTAT\**

**GCAGATTCATTTGTAATTAGAGGTGATGAAGTCAGACAAATCGCTCCAGGGCAAACTGGAAAGATTGCTG\**

**ATTATAATTATAAATTACCAGATGATTTTACAGGCTGCGTTATAGCTTGGAATTCTAACAATCTTGATTC\**

**TAAGGTTGGTGGTAATTATAATTACCTGTATAGATTGTTTAGGAAGTCTAATCTCAAACCTTTTGAGAGA\**

**GATATTTCAACTGAAATCTATCAGGCCGGTAGCACACCTTGTAATGGTGTTGAAGGTTTTAATTGTTACT\**

**TTCCTTTACAATCATATGGTTTCCAACCCACTAATGGTGTTGGTTACCAACCATACAGAGTAGTAGTACT\**

**TTCTTTTGAACTTCTACATGCACCAGCAACTGTTTGTGGACCTAAAAAGTCTACTAATTTGGTTAAAAAC\**

**AAATGTGTCAATTTCAACTTCAATGGTTTAACAGGCACAGGTGTTCTTACTGAGTCTAACAAAAAGTTTC\**

**TGCCTTTCCAACAATTTGGCAGAGACATTGCTGACACTACTGATGCTGTCCGTGATCCACAGACACTTGA\**

**GATTCTTGACATTACACCATGTTCTTTTGGTGGTGTCAGTGTTATAACACCAGGAACAAATACTTCTAAC\**

**CAGGTTGCTGTTCTTTATCAGGATGTTAACTGCACAGAAGTCCCTGTTGCTATTCATGCAGATCAACTTA\**

**CTCCTACTTGGCGTGTTTATTCTACAGGTTCTAATGTTTTTCAAACACGTGCAGGCTGTTTAATAGGGGC\**

**TGAACATGTCAACAACTCATATGAGTGTGACATACCCATTGGTGCAGGTATATGCGCTAGTTATCAGACT\**

**CAGACTAATTCTCCTCGGCGGGCACGTAGTGTAGCTAGTCAATCCATCATTGCCTACACTATGTCACTTG\**

**GTGCAGAAAATTCAGTTGCTTACTCTAATAACTCTATTGCCATACCCACAAATTTTACTATTAGTGTTAC\**

**CACAGAAATTCTACCAGTGTCTATGACCAAGACATCAGTAGATTGTACAATGTACATTTGTGGTGATTCA\**

**ACTGAATGCAGCAATCTTTTGTTGCAATATGGCAGTTTTTGTACACAATTAAACCGTGCTTTAACTGGAA\**

**TAGCTGTTGAACAAGACAAAAACACCCAAGAAGTTTTTGCACAAGTCAAACAAATTTACAAAACACCACC\**

**AATTAAAGATTTTGGTGGTTTTAATTTTTCACAAATATTACCAGATCCATCAAAACCAAGCAAGAGGTCA\**

**TTTATTGAAGATCTACTTTTCAACAAAGTGACACTTGCAGATGCTGGCTTCATCAAACAATATGGTGATT\**

**GCCTTGGTGATATTGCTGCTAGAGACCTCATTTGTGCACAAAAGTTTAACGGCCTTACTGTTTTGCCACC\**

**TTTGCTCACAGATGAAATGATTGCTCAATACACTTCTGCACTGTTAGCGGGTACAATCACTTCTGGTTGG\**

**ACCTTTGGTGCAGGTGCTGCATTACAAATACCATTTGCTATGCAAATGGCTTATAGGTTTAATGGTATTG\**

**GAGTTACACAGAATGTTCTCTATGAGAACCAAAAATTGATTGCCAACCAATTTAATAGTGCTATTGGCAA\**

**AATTCAAGACTCACTTTCTTCCACAGCAAGTGCACTTGGAAAACTTCAAGATGTGGTCAACCAAAATGCA\**

**CAAGCTTTAAACACGCTTGTTAAACAACTTAGCTCCAATTTTGGTGCAATTTCAAGTGTTTTAAATGATA\**

**TCCTTTCACGTCTTGACAAAGTTGAGGCTGAAGTGCAAATTGATAGGTTGATCACAGGCAGACTTCAAAG\**

**TTTGCAGACATATGTGACTCAACAATTAATTAGAGCTGCAGAAATCAGAGCTTCTGCTAATCTTGCTGCT\**

**ACTAAAATGTCAGAGTGTGTACTTGGACAATCAAAAAGAGTTGATTTTTGTGGAAAGGGCTATCATCTTA\**

**TGTCCTTCCCTCAGTCAGCACCTCATGGTGTAGTCTTCTTGCATGTGACTTATGTCCCTGCACAAGAAAA\**

**GAACTTCACAACTGCTCCTGCCATTTGTCATGATGGAAAAGCACACTTTCCTCGTGAAGGTGTCTTTGTT\**

**TCAAATGGCACACACTGGTTTGTAACACAAAGGAATTTTTATGAACCACAAATCATTACTACAGACAACA\**

**CATTTGTGTCTGGTAACTGTGATGTTGTAATAGGAATTGTCAACAACACAGTTTATGATCCTTTGCAACC\**

**TGAATTAGACTCATTCAAGGAGGAGTTAGATAAATATTTTAAGAATCATACATCACCAGATGTTGATTTA\**

**GGTGACATCTCTGGCATTAATGCTTCAGTTGTAAACATTCAAAAAGAAATTGACCGCCTCAATGAGGTTG\**

**CCAAGAATTTAAATGAATCTCTCATCGATCTCCAAGAACTTGGAAAGTATGAGCAGTATATAAAATGGCC\**

**ATGGTACATTTGGCTAGGTTTTATAGCTGGCTTGATTGCCATAGTAATGGTGACAATTATGCTTTGCTGT\**

**ATGACCAGTTGCTGTAGTTGTCTCAAGGGCTGTTGTTCTTGTGGATCCTGCTGCAAATTTGATGAAGACG\**

**ACTCTGAGCCAGTGCTCAAAGGAGTCAAATTACATTACACATAAACGAACTTATGGATTTGTTTATGAGA\**

**ATCTTCACAATTGGAACTGTAACTTTGAAGCAAGGTGAAATCAAGGATGCTACTCCTTCAGATTTTGTTC\**

**GCGCTACTGCAACGATACCGATACAAGCCTCACTCCCTTTCGGATGGCTTATTGTTGGCGTTGCACTTCT\**

**TGCTGTTTTTCAGAGCGCTTCCAAAATCATAACCCTCAAAAAGAGATGGCAACTAGCACTCTCCAAGGGT\**

**GTTCACTTTGTTTGCAACTTGCTGTTGTTGTTTGTAACAGTTTACTCACACCTTTTGCTCGTTGCTGCTG\**

**GCCTTGAAGCCCCTTTTCTCTATCTTTATGCTTTAGTCTACTTCTTGCAGAGTATAAACTTTGTAAGAAT\**

**AATAATGAGGCTTTGGCTTTGCTGGAAATGCCGTTCCAAAAACCCATTACTTTATGATGCCAACTATTTT\**

**CTTTGCTGGCATACTAATTGTTACGACTATTGTATACCTTACAATAGTGTAACTTCTTCAATTGTCATTA\**

**CTTCAGGTGATGGCACAACAAGTCCTATTTCTGAACATGACTACCAGATTGGTGGTTATACTGAAAAATG\**

**GGAATCTGGAGTAAAAGACTGTGTTGTATTACACAGTTACTTCACTTCAGACTATTACCAGCTGTACTCA\**

**ACTCAATTGAGTACAGACACTGGTGTTGAACATGTTACCTTCTTCATCTACAATAAAATTGTTGATGAGC\**

**CTGAAGAACATGTCCAAATTCACACAATCGACGGTTCATCCGGAGTTGTTAATCCAGTAATGGAACCAAT\**

**TTATGATGAACCGACGACGACTACTAGCGTGCCTTTGTAAGCACAAGCTGATGAGTACGAACTTATGTAC\**

**TCATTCGTTTCGGAAGAGACAGGTACGTTAATAGTTAATAGCGTACTTCTTTTTCTTGCTTTCGTGGTAT\**

**TCTTGCTAGTTACACTAGCCATCCTTACTGCGCTTCGATTGTGTGCGTACTGCTGCAATATTGTTAACGT\**

**GAGTCTTGTAAAACCTTCTTTTTACGTTTACTCTCGTGTTAAAAATCTGAATTCTTCTAGAGTTCCTGAT\**

**CTTCTGGTCTAAACGAACTAAATATTATATTAGTTTTTCTGTTTGGAACTTTAATTTTAGCCATGGCAGA\**

**TTCCAACGGTACTATTACCGTTGAAGAGCTTAAAAAGCTCCTTGAACAATGGAACCTAGTAATAGGTTTC\**

**CTATTCCTTACATGGATTTGTCTTCTACAATTTGCCTATGCCAACAGGAATAGGTTTTTGTATATAATTA\**

**AGTTAATTTTCCTCTGGCTGTTATGGCCAGTAACTTTAGCTTGTTTTGTGCTTGCTGCTGTTTACAGAAT\**

**AAATTGGATCACCGGTGGAATTGCTATCGCAATGGCTTGTCTTGTAGGCTTGATGTGGCTCAGCTACTTC\**

**ATTGCTTCTTTCAGACTGTTTGCGCGTACGCGTTCCATGTGGTCATTCAATCCAGAAACTAACATTCTTC\**

**TCAACGTGCCACTCCATGGCACTATTCTGACCAGACCGCTTCTAGAAAGTGAACTCGTAATCGGAGCTGT\**

**GATCCTTCGTGGACATCTTCGTATTGCTGGACACCATCTAGGACGCTGTGACATCAAGGACCTGCCTAAA\**

**GAAATCACTGTTGCTACATCACGAACGCTTTCTTATTACAAATTGGGAGCTTCGCAGCGTGTAGCAGGTG\**

**ACTCAGGTTTTGCTGCATACAGTCGCTACAGGATTGGCAACTATAAATTAAACACAGACCATTCCAGTAG\**

**CAGTGACAATATTGCTTTGCTTGTACAGTAAGTGACAACAGATGTTTCATCTCGTTGACTTTCAGGTTAC\**

**TATAGCAGAGATATTACTAATTATTATGAGGACTTTTAAAGTTTCCATTTGGAATCTTGATTACATCATA\**

**AACCTCATAATTAAAAATTTATCTAAGTCACTAACTGAGAATAAATATTCTCAATTAGATGAAGAGCAAC\**

**CAATGGAGATTGATTAAACGAACATGAAAATTATTCTTTTCTTGGCACTGATAACACTCGCTACTTGTGA\**

**GCTTTATCACTACCAAGAGTGTGTTAGAGGTACAACAGTACTTTTAAAAGAACCTTGCTCTTCTGGAACA\**

**TACGAGGGCAATTCACCATTTCATCCTCTAGCTGATAACAAATTTGCACTGACTTGCTTTAGCACTCAAT\**

**TTGCTTTTGCTTGTCCTGACGGCGTAAAACACGTCTATCAGTTACGTGCCAGATCAGTTTCACCTAAACT\**

**GTTCATCAGACAAGAGGAAGTTCAAGAACTTTACTCTCCAATTTTTCTTATTGTTGCGGCAATAGTGTTT\**

**ATAACACTTTGCTTCACACTCAAAAGAAAGACAGAATGATTGAACTTTCATTAATTGACTTCTATTTGTG\**

**CTTTTTAGCCTTTCTGCTATTCCTTGTTTTAATTATGCTTATTATCTTTTGGTTCTCACTTGAACTGCAA\**

**GATCATAATGAAACTTGTCACGCCTAAACGAACATGAAATTTCTTGTTTTCTTAGGAATCATCACAACTG\**

**TAGCTGCATTTCACCAAGAATGTAGTTTACAGTCATGTACTCAACATCAACCATATGTAGTTGATGACCC\**

**GTGTCCTATTCACTTCTATTCTAAATGGTATATTAGAGTAGGAGCTAGAAAATCAGCACCTTTAATTGAA\**

**TTGTGCGTGGATGAGGCTGGTTCTAAATCACCCATTCAGTACATCGATATCGGTAATTATACAGTTTCCT\**

**GTTTACCTTTTACAATTAATTGCCAGGAACCTAAATTGGGTAGTCTTGTAGTGCGTTGTTCGTTCTATGA\**

**AGACTTTTTAGAGTATCATGACGTTCGTGTTGTTTTAGATTTCATCTAAACGAACAAACTAAAATGTCTG\**

**ATAATGGACCCCAAAATCAGCGAAATGCACCCCGCATTACGTTTGGTGGACCCTCAGATTCAACTGGCAG\**

**TAACCAGAATGGAGAACGCAGTGGGGCGCGATCAAAACAACGTCGGCCCCAAGGTTTACCCAATAATACT\**

**GCGTCTTGGTTCACCGCTCTCACTCAACATGGCAAGGAAGACCTTAAATTCCCTCGAGGACAAGGCGTTC\**

**CAATTAACACCAATAGCAGTCCAGATGACCAAATTGGCTACTACCGAAGAGCTACCAGACGAATTCGTGG\**

**TGGTGACGGTAAAATGAAAGATCTCAGTCCAAGATGGTATTTCTACTACCTAGGAACTGGGCCAGAAGCT\**

**GGACTTCCCTATGGTGCTAACAAAGACGGCATCATATGGGTTGCAACTGAGGGAGCCTTGAATACACCAA\**

**AAGATCACATTGGCACCCGCAATCCTGCTAACAATGCTGCAATCGTGCTACAACTTCCTCAAGGAACAAC\**

**ATTGCCAAAAGGCTTCTACGCAGAAGGGAGCAGAGGCGGCAGTCAAGCCTCTTCTCGTTCCTCATCACGT\**

**AGTCGCAACAGTTCAAGAAATTCAACTCCAGGCAGCAGTAGGGGAACTTCTCCTGCTAGAATGGCTGGCA\**

**ATGGCGGTGATGCTGCTCTTGCTTTGCTGCTGCTTGACAGATTGAACCAGCTTGAGAGCAAAATGTCTGG\**

**TAAAGGCCAACAACAACAAGGCCAAACTGTCACTAAGAAATCTGCTGCTGAGGCTTCTAAGAAGCCTCGG\**

**CAAAAACGTACTGCCACTAAAGCATACAATGTAACACAAGCTTTCGGCAGACGTGGTCCAGAACAAACCC\**

**AAGGAAATTTTGGGGACCAGGAACTAATCAGACAAGGAACTGATTACAAACATTGGCCGCAAATTGCACA\**

**ATTTGCCCCCAGCGCTTCAGCGTTCTTCGGAATGTCGCGCATTGGCATGGAAGTCACACCTTCGGGAACG\**

**TGGTTGACCTACACAGGTGCCATCAAATTGGATGACAAAGATCCAAATTTCAAAGATCAAGTCATTTTGC\**

**TGAATAAGCATATTGACGCATACAAAACATTCCCACCAACAGAGCCTAAAAAGGACAAAAAGAAGAAGGC\**

**TGATGAAACTCAAGCCTTACCGCAGAGACAGAAGAAACAGCAAACTGTGACTCTTCTTCCTGCTGCAGAT\**

**TTGGATGATTTCTCCAAACAATTGCAACAATCCATGAGCAGTGCTGACTCAACTCAGGCCTAAACTCATG\**

**CAGACCACACAAGGCAGATGGGCTATATAAACGTTTTCGCTTTTCCGTTTACGATATATAGTCTACTCTT\**

**GTGCAGAATGAATTCTCGTAACTACATAGCACAAGTAGATGTAGTTAACTTTAATCTCACATAGCAATCT\**

**TTAATCAGTGTGTAACATTAGGGAGGACTTGAAAGAGCCACCACATTTTCACCGAGGCCACGCGGAGTAC\**

**GATCGAGTGTACAGTGAACAATGCTAGGGAGAGCTGCCTATATGGAAGAGCCCTAATGTGTAAAATTAAT\**

**TTTAGTAGTGCTATCCCCATGTGATTTTAATAGCTTCTTAGGAGAATGACAAAAAAAAAAAAAAAAAAAA\**

**AAAAAAAAAAAAA\**

**MERS**

**ATTTAAGTGAATAGCTTGGCTATCTCACTTCCCCTCGTTCTCTTGCAGAACTTTGATTTT**

**AACGAACTTAAATAAAAGCCCTGTTGTTTAGCGTATTGTTGCACTTGTCTGGTGGGATTG**

**TGGCATTAATTTGCCTGCTCATCTAGGCAGTGGACATATGCTCAACACTGGGTATAATTC**

**TAATTGAATACTATTTTTCAGTTAGAGCGTCGTGTCTCTTGTACGTCTCGGTCACAATAC**

**ACGGTTTCGTCCGGTGCGTGGCAATTCGGGGCACATCATGTCTTTCGTGGCTGGTGTGAC**

**CGCGCAAGGTGCGCGCGGTACGTATCGAGCAGCGCTCAACTCTGAAAAACATCAAGACCA**

**TGTGTCTCTAACTGTGCCACTCTGTGGTTCAGGAAACCTGGTTGAAAAACTTTCACCATG**

**GTTCATGGATGGCGAAAATGCCTATGAAGTGGTGAAGGCCATGTTACTTAAAAAGGAGCC**

**ACTTCTCTATGTGCCCATCCGGCTGGCTGGACACACTAGACACCTCCCAGGTCCTCGTGT**

**ATACCTGGTTGAGAGGCTCATTGCTTGTGAAAATCCATTCATGGTTAACCAATTGGCTTA**

**TAGCTCTAGTGCAAATGGCAGCTTGGTTGGCACAACTTTGCAGGGCAAGCCTATTGGTAT**

**GTTCTTCCCTTATGACATCGAACTTGTCACAGGAAAGCAAAATATTCTCCTGCGCAAGTA**

**TGGCCGTGGTGGTTATCACTACACCCCATTCCACTATGAGCGAGACAACACCTCTTGCCC**

**TGAGTGGATGGACGATTTTGAGGCGGATCCTAAAGGCAAATATGCCCAGAATCTGCTTAA**

**GAAGTTGATTGGCGGTGATGTCACTCCAGTTGACCAATACATGTGTGGCGTTGATGGAAA**

**ACCCATTAGTGCCTACGCATTTTTAATGGCCAAGGATGGAATAACCAAACTGGCTGATGT**

**TGAAGCGGACGTCGCAGCACGTGCTGATGACGAAGGCTTCATCACATTAAAGAACAATCT**

**ATATAGATTGGTTTGGCATGTTGAGCGTAAAGACGTTCCATATCCTAAGCAATCTATTTT**

**TACTATTAATAGTGTGGTCCAAAAGGATGGTGTTGAAAACACTCCTCCTCACTATTTTAC**

**TCTTGGATGCAAAATTTTAACGCTCACCCCACGCAACAAGTGGAGTGGCGTTTCTGACTT**

**GTCCCTCAAACAAAAACTCCTTTACACCTTCTATGGTAAGGAGTCACTTGAGAACCCAAC**

**CTACATTTACCACTCCGCATTCATTGAGTGTGGAAGTTGTGGTAATGATTCCTGGCTTAC**

**AGGGAATGCTATCCAAGGGTTTGCCTGTGGATGTGGGGCATCATATACAGCTAATGATGT**

**CGAAGTCCAATCATCTGGCATGATTAAGCCAAATGCTCTTCTTTGTGCTACTTGCCCCTT**

**TGCTAAGGGTGATAGCTGTTCTTCTAATTGCAAACATTCAGTTGCTCAGTTGGTTAGTTA**

**CCTTTCTGAACGTTGTAATGTTATTGCTGATTCTAAGTCCTTCACACTTATCTTTGGTGG**

**CGTAGCTTACGCCTACTTTGGATGTGAGGAAGGTACTATGTACTTTGTGCCTAGAGCTAA**

**GTCTGTTGTCTCAAGGATTGGAGACTCCATCTTTACAGGCTGTACTGGCTCTTGGAACAA**

**GGTCACTCAAATTGCTAACATGTTCTTGGAACAGACTCAGCATTCCCTTAACTTTGTGGG**

**AGAGTTCGTTGTCAACGATGTTGTCCTCGCAATTCTCTCTGGAACCACAACTAATGTTGA**

**CAAAATACGCCAGCTTCTCAAAGGTGTCACCCTTGACAAGTTGCGTGATTATTTAGCTGA**

**CTATGACGTAGCAGTCACTGCCGGCCCATTCATGGATAATGCTATTAATGTTGGTGGTAC**

**AGGATTACAGTATGCCGCCATTACTGCACCTTATGTAGTTCTCACTGGCTTAGGTGAGTC**

**CTTTAAGAAAGTTGCAACCATACCGTACAAGGTTTGCAACTCTGTTAAGGATACTCTGAC**

**TTATTATGCTCACAGCGTGTTGTACAGAGTTTTTCCTTATGACATGGATTCTGGTGTGTC**

**ATCCTTTAGTGAACTACTTTTTGATTGCGTTGATCTTTCAGTAGCTTCTACCTATTTTTT**

**AGTCCGCCTCTTGCAAGATAAGACTGGCGACTTTATGTCTACAATTATTACTTCCTGCCA**

**AACTGCTGTTAGTAAGCTTCTAGATACATGTTTTGAAGCTACAGAAGCAACATTTAACTT**

**CTTGTTAGATTTGGCAGGATTGTTCAGAATCTTTCTTCGCAATGCCTATGTGTACACTTC**

**ACAAGGGTTTGTGGTGGTCAATGGCAAAGTTTCTACACTTGTCAAACAAGTGTTAGACTT**

**GCTTAATAAGGGTATGCAACTTTTGCATACAAAGGTCTCCTGGGCTGGTTCTAATATCAG**

**TGCTGTTATCTACAGCGGCAGGGAGTCTCTAATATTCCCATCGGGAACCTATTACTGTGT**

**CACCACTAAGGCTAAGTCCGTTCAACAAGATCTTGACGTTATTTTGCCTGGTGAGTTTTC**

**CAAGAAGCAGTTAGGACTGCTCCAACCTACTGACAATTCTACAACTGTTAGTGTTACTGT**

**ATCCAGTAACATGGTTGAAACTGTTGTGGGTCAACTTGAGCAAACTAATATGCATAGTCC**

**TGATGTTATAGTAGGTGACTATGTCATTATTAGTGAAAAATTGTTTGTGCGTAGTAAGGA**

**AGAAGACGGATTTGCCTTCTACCCTGCTTGCACTAATGGTCATGCTGTACCGACTCTCTT**

**TAGACTTAAGGGAGGTGCACCTGTAAAAAAAGTAGCCTTTGGCGGTGATCAAGTACATGA**

**GGTTGCTGCTGTAAGAAGTGTTACTGTCGAGTACAACATTCATGCTGTATTAGACACACT**

**ACTTGCTTCTTCTAGTCTTAGAACCTTTGTTGTAGATAAGTCTTTGTCAATTGAGGAGTT**

**TGCTGACGTAGTAAAGGAACAAGTCTCAGACTTGCTTGTTAAATTACTGCGTGGAATGCC**

**GATTCCAGATTTTGATTTAGACGATTTTATTGACGCACCATGCTATTGCTTTAACGCTGA**

**GGGTGATGCATCTTGGTCTTCTACTATGATCTTCTCTCTTCACCCCGTCGAGTGTGACGA**

**GGAGTGTTCTGAAGTAGAGGCTTCAGATTTAGAAGAAGGTGAATCAGAGTGCATTTCTGA**

**GACTTCAACTGAACAAGTTGACGTTTCTCATGAGATTTCTGACGACGAGTGGGCTGCTGC**

**AGTTGATGAAGCGTTCCCCCTCGATGAAGCAGAAGATGTTACTGAATCTGTGCAAGAAGA**

**AGCACAACCAGTAGAAGTACCTGTTGAAGATATTGCGCAGGTTGTCATAGCTGACACCTT**

**ACAGGAAACTCCTGTTGTGTCTGATACTGTTGAAGTCCCACCGCAAGTGGTGAAACTTCC**

**GTCTGAACCTCAGACTATCCAGCCCGAGGTAAAAGAAGTTGCACCTGTCTATGAGGCTGA**

**TACCGAACAGACACAGAGTGTTACTGTTAAACCTAAGAGGTTACGCAAAAAGCGTAATGT**

**TGACCCTTTGTCCAATTTTGAACATAAGGTTATTACAGAGTGCGTTACCATAGTTTTAGG**

**TGACGCAATTCAAGTAGCCAAGTGCTATGGGGAGTCTGTGTTAGTTAATGCTGCTAACAC**

**ACATCTTAAGCATGGCGGTGGTATCGCTGGTGCTATTAATGCGGCTTCAAAAGGGGCTGT**

**CCAAAAAGAGTCAGATGAGTATATTCTGGCTAAAGGGCCGTTACAAGTAGGAGATTCAGT**

**TCTCTTGCAAGGCCATTCTCTAGCTAAGAATATCCTGCATGTCGTAGGCCCAGATGCCCG**

**CGCTAAACAGGATGTTTCTCTCCTTAGTAAGTGCTATAAGGCTATGAATGCATATCCTCT**

**TGTAGTCACTCCTCTTGTTTCAGCAGGCATATTTGGTGTAAAACCAGCTGTGTCTTTTGA**

**TTATCTTATTAGAGAGGCTAAGACTAGAGTTTTAGTCGTCGTTAATTCCCAAGATGTCTA**

**TAAGAGTCTTACCATAGTTGACATTCCACAGAGTTTGACTTTTTCATATGATGGGTTACG**

**TGGCGCAATACGTAAAGCTAAAGATTATGGTTTTACTGTTTTTGTGTGCACAGACAACTC**

**TGCTAACACTAAAGTTCTTAGGAACAAGGGTGTTGATTATACTAAGAAGTTTCTTACAGT**

**TGACGGTGTGCAATATTATTGCTACACGTCTAAGGACACTTTAGATGATATCTTACAACA**

**GGCTAATAAGTCTGTTGGTATTATATCTATGCCTTTGGGATATGTGTCTCATGGTTTAGA**

**CTTAATTCAAGCAGGGAGTGTCGTGCGTAGAGTTAACGTGCCCTACGTGTGTCTCCTAGC**

**TAATAAAGAGCAAGAAGCTATTTTGATGTCTGAAGACGTTAAGTTAAACCCTTCAGAAGA**

**TTTTATAAAGCACGTCCGCACTAATGGTGGTTACAATTCTTGGCATTTAGTCGAGGGTGA**

**ACTATTGGTGCAAGACTTACGCTTAAATAAGCTCCTGCATTGGTCTGATCAAACCATATG**

**CTACAAGGATAGTGTGTTTTATGTTGTAAAGAATAGTACAGCTTTTCCATTTGAAACACT**

**TTCAGCATGTCGTGCGTATTTGGATTCACGCACGACACAGCAGTTAACAATCGAAGTCTT**

**AGTGACTGTCGATGGTGTAAATTTTAGAACAGTCGTTCTAAATAATAAGAACACTTATAG**

**ATCACAGCTTGGATGCGTTTTCTTTAATGGTGCTGATATTTCTGATACCATTCCTGATGA**

**GAAACAGAATGGTCACAGTTTATATCTAGCAGACAATTTGACTGCTGATGAAACAAAGGC**

**GCTTAAAGAGTTATATGGCCCCGTTGATCCTACTTTCTTACACAGATTCTATTCACTTAA**

**GGCTGCAGTCCATAAGTGGAAGATGGTTGTGTGTGATAAGGTACGTTCTCTCAAATTGAG**

**TGATAATAATTGTTATCTTAATGCAGTTATTATGACACTTGATTTATTGAAGGACATTAA**

**ATTTGTTATACCTGCTCTACAGCATGCATTTATGAAACATAAGGGCGGTGATTCAACTGA**

**CTTCATAGCCCTCATTATGGCTTATGGCAATTGCACATTTGGTGCTCCAGATGATGCCTC**

**TCGGTTACTTCATACCGTGCTTGCAAAGGCTGAGTTATGCTGTTCTGCACGCATGGTTTG**

**GAGAGAGTGGTGCAATGTCTGTGGCATAAAAGATGTTGTTCTACAAGGCTTAAAAGCTTG**

**TTGTTACGTGGGTGTGCAAACTGTTGAAGATCTGCGTGCTCGCATGACATATGTATGCCA**

**GTGTGGTGGTGAACGTCATCGGCAAATAGTCGAACACACCACCCCCTGGTTGCTGCTCTC**

**AGGCACACCAAATGAAAAATTGGTGACAACCTCCACGGCGCCTGATTTTGTAGCGTTTAA**

**TGTCTTTCAGGGCATTGAAACGGCTGTTGGCCATTATGTTCATGCTCGCCTGAAGGGTGG**

**TCTTATTTTAAAGTTTGACTCTGGCACCGTTAGCAAGACTTCAGACTGGAAGTGCAAGGT**

**GACAGATGTACTTTTCCCCGGCCAAAAATACAGTAGCGATTGTAATGTCGTACGGTATTC**

**TTTGGACGGTAATTTCAGAACAGAGGTTGATCCCGACCTATCTGCTTTCTATGTTAAGGA**

**TGGTAAATACTTTACAAGTGAACCACCCGTAACATATTCACCAGCTACAATTTTAGCTGG**

**TAGTGTCTACACTAATAGCTGCCTTGTATCGTCTGATGGACAACCTGGCGGTGATGCTAT**

**TAGTTTGAGTTTTAATAACCTTTTAGGGTTTGATTCTAGTAAACCAGTCACTAAGAAATA**

**CACTTACTCCTTCTTGCCTAAAGAAGACGGCGATGTGTTGTTGGCTGAGTTTGACACTTA**

**TGACCCTATTTATAAGAATGGTGCCATGTATAAAGGCAAACCAATTCTTTGGGTCAACAA**

**AGCATCTTATGATACTAATCTTAATAAGTTCAATAGAGCTAGTTTGCGTCAAATTTTTGA**

**CGTAGCCCCCATTGAACTCGAAAATAAATTCACACCTTTGAGTGTGGAGTCTACACCAGT**

**TGAACCTCCAACTGTAGATGTGGTAGCACTTCAACAGGAAATGACAATTGTCAAATGTAA**

**GGGTTTAAATAAACCTTTCGTGAAGGACAATGTCAGTTTCGTTGCTGATGACTCAGGTAC**

**TCCCGTTGTTGAGTATCTGTCTAAAGAAGATCTACATACATTGTATGTAGACCCTAAGTA**

**TCAAGTCATTGTCTTAAAAGACAATGTACTTTCTTCTATGCTTAGATTGCACACCGTTGA**

**GTCAGGTGATATTAACGTTGTTGCAGCTTCCGGATCTTTGACACGTAAAGTGAAGTTACT**

**ATTTAGGGCTTCATTTTATTTCAAAGAATTTGCTACCCGCACTTTCACTGCTACCACTGC**

**TGTAGGTAGTTGTATAAAGAGTGTAGTGCGGCATCTAGGTGTTACTAAAGGCATATTGAC**

**AGGCTGTTTTAGTTTTGTCAAGATGTTATTTATGCTTCCACTAGCTTACTTTAGTGATTC**

**AAAACTCGGCACCACAGAGGTTAAAGTGAGTGCTTTGAAAACAGCTGGCGTTGTGACAGG**

**TAATGTTGTAAAACAGTGTTGCACTGCTGCTGTTGATTTAAGTATGGATAAGTTGCGCCG**

**TGTGGATTGGAAATCAACCCTACGGTTGTTACTTATGTTATGCACAACTATGGTATTGTT**

**GTCTTCTGTGTATCACTTGTATGTCTTCAATCAGGTCTTATCAAGTGATGTTATGTTTGA**

**AGATGCCCAAGGTTTGAAAAAGTTCTACAAAGAAGTTAGAGCTTACCTAGGAATCTCTTC**

**TGCTTGTGACGGTCTTGCTTCAGCTTATAGGGCGAATTCCTTTGATGTACCTACATTCTG**

**CGCAAACCGTTCTGCAATGTGTAATTGGTGCTTGATTAGCCAAGATTCCATAACTCACTA**

**CCCAGCTCTTAAGATGGTTCAAACACATCTTAGCCACTATGTTCTTAACATAGATTGGTT**

**GTGGTTTGCATTTGAGACTGGTTTGGCATACATGCTCTATACCTCGGCCTTCAACTGGTT**

**GTTGTTGGCAGGTACATTGCATTATTTCTTTGCACAGACTTCCATATTTGTAGACTGGCG**

**GTCATACAATTATGCTGTGTCTAGTGCCTTCTGGTTATTCACCCACATTCCAATGGCGGG**

**TTTGGTACGAATGTATAATTTGTTAGCATGCCTTTGGCTTTTACGCAAGTTTTATCAGCA**

**TGTAATCAATGGTTGCAAAGATACGGCATGCTTGCTCTGCTATAAGAGGAACCGACTTAC**

**TAGAGTTGAAGCTTCTACCGTTGTCTGTGGTGGAAAACGTACGTTTTATATCACAGCAAA**

**TGGCGGTATTTCATTCTGTCGTAGGCATAATTGGAATTGTGTGGATTGTGACACTGCAGG**

**TGTGGGGAATACCTTCATCTGTGAAGAAGTCGCAAATGACCTCACTACCGCCCTACGCAG**

**GCCTATTAACGCTACGGATAGATCACATTATTATGTGGATTCCGTTACAGTTAAAGAGAC**

**TGTTGTTCAGTTTAATTATCGTAGAGACGGTCAACCATTCTACGAGCGGTTTCCCCTCTG**

**CGCTTTTACAAATCTAGATAAGTTGAAGTTCAAAGAGGTCTGTAAAACTACTACTGGTAT**

**ACCTGAATACAACTTTATCATCTACGACTCATCAGATCGTGGCCAGGAAAGTTTAGCTAG**

**GTCTGCATGTGTTTATTATTCTCAAGTCTTGTGTAAATCAATTCTTTTGGTTGACTCAAG**

**TTTGGTTACTTCTGTTGGTGATTCTAGTGAAATCGCCACTAAAATGTTTGATTCCTTTGT**

**TAATAGTTTCGTCTCGCTGTATAATGTCACACGCGATAAGTTGGAAAAACTTATCTCTAC**

**TGCTCGTGATGGCGTAAGGCGAGGCGATAACTTCCATAGTGTCTTAACAACATTCATTGA**

**CGCAGCACGAGGCCCCGCAGGTGTGGAGTCTGATGTTGAGACCAATGAAATTGTTGACTC**

**TGTGCAGTATGCTCATAAACATGACATACAAATTACTAATGAGAGTTACAATAATTATGT**

**ACCCTCATATGTTAAACCTGATAGTGTGTCTACCAGTGATTTAGGTAGTCTCATTGATTG**

**TAATGCGGCTTCAGTTAACCAAATTGTCTTGCGTAATTCTAATGGTGCTTGTATTTGGAA**

**CGCTGCTGCATATATGAAACTCTCGGATGCACTTAAACGACAGATTCGCATTGCATGCCG**

**TAAGTGTAATTTAGCTTTCCGGTTAACCACCTCAAAGCTACGCGCTAATGATAATATCTT**

**ATCAGTTAGATTCACTGCTAACAAAATTGTTGGTGGTGCTCCTACATGGTTTAATGCGTT**

**GCGTGACTTTACGTTAAAGGGTTACGTTCTTGCTACCATTATTGTGTTTCTGTGTGCTGT**

**ACTGATGTATTTGTGTTTACCTACATTTTCTATGGTACCTGTTGAATTTTATGAAGACCG**

**CATCTTGGACTTTAAAGTTCTTGATAATGGTATCATTAGGGATGTAAATCCTGATGATAA**

**GTGCTTTGCTAATAAGCACCGGTCCTTCACACAATGGTATCATGAGCATGTTGGTGGTGT**

**CTATGACAACTCTATCACATGCCCATTGACAGTTGCAGTAATTGCTGGAGTTGCTGGTGC**

**TCGCATTCCAGACGTACCTACTACATTGGCTTGGGTGAACAATCAGATAATTTTCTTTGT**

**TTCTCGAGTCTTTGCTAATACAGGCAGTGTTTGCTACACTCCTATAGATGAGATACCCTA**

**TAAGAGTTTCTCTGATAGTGGTTGCATTCTTCCATCTGAGTGCACTATGTTTAGGGATGC**

**AGAGGGCCGTATGACACCATACTGCCATGATCCTACTGTTTTGCCTGGGGCTTTTGCGTA**

**CAGTCAGATGAGGCCTCATGTTCGTTACGACTTGTATGATGGTAACATGTTTATTAAATT**

**TCCTGAAGTAGTATTTGAAAGTACACTTAGGATTACTAGAACTCTGTCAACTCAGTACTG**

**CCGGTTCGGTAGTTGTGAGTATGCACAAGAGGGTGTTTGTATTACCACAAATGGCTCGTG**

**GGCCATTTTTAATGACCACCATCTTAATAGACCTGGTGTCTATTGTGGCTCTGATTTTAT**

**TGACATTGTCAGGCGGTTAGCAGTATCACTGTTCCAGCCTATTACTTATTTCCAATTGAC**

**TACCTCATTGGTCTTGGGTATAGGTTTGTGTGCGTTCCTGACTTTGCTCTTCTATTATAT**

**TAATAAAGTAAAACGTGCTTTTGCAGATTACACCCAGTGTGCTGTAATTGCTGTTGTTGC**

**TGCTGTTCTTAATAGCTTGTGCATCTGCTTTGTTGCCTCTATACCATTGTGTATAGTACC**

**TTACACTGCATTGTACTATTATGCTACATTCTATTTTACTAATGAGCCTGCATTTATTAT**

**GCATGTTTCTTGGTACATTATGTTCGGGCCTATCGTTCCCATATGGATGACCTGCGTCTA**

**TACAGTTGCAATGTGCTTTAGACACTTCTTCTGGGTTTTAGCTTATTTTAGTAAGAAACA**

**TGTAGAAGTTTTTACTGATGGTAAGCTTAATTGTAGTTTCCAGGACGCTGCCTCTAATAT**

**CTTTGTTATTAACAAGGACACTTATGCAGCTCTTAGAAACTCTTTAACTAATGATGCCTA**

**TTCACGATTTTTGGGGTTGTTTAACAAGTATAAGTACTTCTCTGGTGCTATGGAAACAGC**

**CGCTTATCGTGAAGCTGCAGCATGTCATCTTGCTAAAGCCTTACAAACATACAGCGAGAC**

**TGGTAGTGATCTTCTTTACCAACCACCCAACTGTAGCATAACCTCTGGCGTGTTGCAAAG**

**CGGTTTGGTGAAAATGTCACATCCCAGTGGAGATGTTGAGGCTTGTATGGTTCAGGTTAC**

**CTGCGGTAGCATGACTCTTAATGGTCTTTGGCTTGACAACACAGTCTGGTGCCCACGACA**

**CGTAATGTGCCCGGCTGACCAGTTGTCTGATCCTAATTATGATGCCTTGTTGATTTCTAT**

**GACTAATCATAGTTTCAGTGTGCAAAAACACATTGGCGCTCCAGCAAACTTGCGTGTTGT**

**TGGTCATGCCATGCAAGGCACTCTTTTGAAGTTGACTGTCGATGTTGCTAACCCTAGCAC**

**TCCAGCCTACACTTTTACAACAGTGAAACCTGGCGCAGCATTTAGTGTGTTAGCATGCTA**

**TAATGGTCGTCCGACTGGTACATTCACTGTTGTAATGCGCCCTAACTACACAATTAAGGG**

**TTCCTTTCTGTGTGGTTCTTGTGGTAGTGTTGGTTACACCAAGGAGGGTAGTGTGATCAA**

**TTTTTGTTACATGCATCAAATGGAACTTGCTAATGGTACACATACCGGTTCAGCATTTGA**

**TGGTACTATGTATGGTGCCTTTATGGATAAACAAGTGCACCAAGTTCAGTTAACAGACAA**

**ATACTGCAGTGTTAATGTAGTAGCTTGGCTTTACGCAGCAATACTTAATGGTTGCGCTTG**

**GTTTGTAAAACCTAATCGCACTAGTGTTGTTTCTTTTAATGAATGGGCTCTTGCCAACCA**

**ATTCACTGAATTTGTTGGCACTCAATCCGTTGACATGTTAGCTGTCAAAACAGGCGTTGC**

**TATTGAACAGCTGCTTTATGCGATCCAACAACTTTATACTGGGTTCCAGGGAAAGCAAAT**

**CCTTGGCAGTACCATGTTGGAAGATGAATTCACACCTGAGGATGTTAATATGCAGATTAT**

**GGGTGTGGTTATGCAGAGTGGTGTGAGAAAAGTTACATATGGTACTGCGCATTGGTTGTT**

**CGCGACCCTTGTCTCAACCTATGTGATAATCTTACAAGCCACTAAATTTACTTTGTGGAA**

**CTACTTGTTTGAGACTATTCCCACACAGTTGTTCCCACTCTTATTTGTGACTATGGCCTT**

**CGTTATGTTGTTGGTTAAACACAAACACACCTTTTTGACACTTTTCTTGTTGCCTGTGGC**

**TATTTGTTTGACTTATGCAAACATAGTCTACGAGCCCACTACTCCCATTTCGTCAGCGCT**

**GATTGCAGTTGCAAATTGGCTTGCCCCCACTAATGCTTATATGCGCACTACACATACTGA**

**TATTGGTGTCTACATTAGTATGTCACTTGTATTAGTCATTGTAGTGAAGAGATTGTACAA**

**CCCATCACTTTCTAACTTTGCGTTAGCATTGTGCAGTGGTGTAATGTGGTTGTACACTTA**

**TAGCATTGGAGAAGCCTCAAGCCCCATTGCCTATCTGGTTTTTGTCACTACACTCACTAG**

**TGATTATACGATTACAGTCTTTGTTACTGTTAACCTTGCAAAAGTTTGCACTTATGCCAT**

**CTTTGCTTACTCGCCACAGCTTACACTTGTGTTTCCGGAAGTGAAGATGATACTTTTATT**

**ATACACATGTTTAGGTTTCATGTGTACTTGCTATTTTGGTGTCTTCTCTCTTTTGAACCT**

**TAAGCTTAGAGCACCTATGGGTGTCTATGACTTTAAGGTCTCAACACAAGAGTTCAGATT**

**CATGACAGCTAACAATCTAACTGCACCTAGAAATTCTTGGGAGGCTATGGCTCTGAACTT**

**TAAGTTAATAGGTATTGGCGGTACACCTTGTATAAAGGTTGCTGCTATGCAGTCTAAACT**

**TACAGATCTTAAATGCACATCTGTGGTTCTCCTCTCTGTGCTCCAACAGTTACACTTAGA**

**GGCTAATAGTAGGGCCTGGGCTTTCTGTGTTAAATGCCATAATGATATATTGGCAGCAAC**

**AGACCCCAGTGAGGCTTTCGAGAAATTCGTAAGTCTCTTTGCCACTTTAATGACTTTTTC**

**TGGTAATGTAGATCTTGATGCGTTAGCTAGTGATATTTTTGACACTCCTAGCGTACTTCA**

**AGCTACTCTTTCTGAGTTTTCACACTTAGCTACCTTTGCTGAGTTGGAAGCTGCGCAGAA**

**AGCCTATCAGGAAGCTATGGACTCTGGTGACACCTCACCACAAGTTCTTAAGGCTTTGCA**

**GAAGGCTGTTAATATAGCTAAAAACGCCTATGAGAAGGATAAGGCAGTGGCCCGTAAGTT**

**AGAACGTATGGCTGATCAGGCTATGACTTCTATGTATAAGCAAGCACGTGCTGAAGACAA**

**GAAAGCAAAAATTGTCAGTGCTATGCAAACTATGTTGTTTGGTATGATTAAGAAGCTCGA**

**CAACGATGTTCTTAATGGTATCATTTCTAACGCTAGGAATGGTTGTATACCTCTTAGTGT**

**CATTCCACTGTGTGCTTCAAATAAACTTCGCGTTGTAATTCCTGACTTCACCGTCTGGAA**

**TCAGGTAGTCACATATCCCTCGCTTAACTACGCTGGGGCTTTGTGGGACATTACAGTTAT**

**AAACAATGTGGACAATGAAATTGTTAAGTCTTCAGATGTTGTAGACAGCAATGAAAATTT**

**AACATGGCCACTTGTTTTAGAATGCACTAGGGCATCCACTTCTGCCGTTAAGTTGCAAAA**

**TAATGAGATCAAACCTTCAGGTTTAAAAACCATGGTTGTGTCTGCAGGTCAAGAGCAAAC**

**TAACTGTAATACTAGTTCCTTAGCTTATTACGAACCTGTGCAGGGTCGTAAAATGCTGAT**

**GGCTCTTCTTTCTGATAATGCCTATCTCAAATGGGCGCGTGTTGAAGGTAAGGACGGATT**

**TGTTAGTGTAGAGCTACAACCTCCTTGCAAATTCTTGATTGCGGGACCAAAAGGACCTGA**

**AATCCGATATCTCTATTTTGTTAAAAATCTTAACAACCTTCATCGCGGGCAAGTGTTAGG**

**GCACATTGCTGCGACTGTTAGATTGCAAGCTGGTTCTAACACCGAGTTTGCCTCTAATTC**

**TTCGGTGTTGTCACTTGTTAACTTCACCGTTGATCCTCAAAAAGCTTATCTCGATTTCGT**

**CAATGCGGGAGGTGCCCCATTGACAAATTGTGTTAAGATGCTTACTCCTAAAACTGGTAC**

**AGGTATAGCTATATCTGTTAAACCAGAGAGTACAGCTGATCAAGAGACTTATGGTGGAGC**

**TTCAGTGTGTCTCTATTGCCGTGCGCATATAGAACATCCTGATGTCTCTGGTGTTTGTAA**

**ATATAAGGGTAAGTTTGTCCAAATCCCTGCTCAGTGTGTCCGTGACCCTGTGGGATTTTG**

**TTTGTCAAATACCCCCTGTAATGTCTGTCAATATTGGATTGGATATGGGTGCAATTGTGA**

**CTCGCTTAGGCAAGCAGCACTGCCCCAATCTAAAGATTCCAATTTTTTAAACGAGTCCGG**

**GGTTCTATTGTAAATGCCCGAATAGAACCCTGTTCAAGTGGTTTGTCCACTGATGTCGTC**

**TTTAGGGCATTTGACATCTGCAACTATAAGGCTAAGGTTGCTGGTATTGGAAAATACTAC**

**AAGACTAATACTTGTAGGTTTGTAGAATTAGATGACCAAGGGCATCATTTAGACTCCTAT**

**TTTGTCGTTAAGAGGCATACTATGGAGAATTATGAACTAGAGAAGCACTGTTACGATTTG**

**TTACGTGACTGTGATGCTGTAGCTCCCCATGATTTCTTCATCTTTGATGTAGACAAAGTT**

**AAAACACCTCATATTGTACGTCAGCGTTTAACTGAGTACACTATGATGGATCTTGTATAT**

**GCCCTGAGGCACTTTGATCAAAATAGCGAAGTGCTTAAGGCTATCTTAGTGAAGTATGGT**

**TGCTGTGATGTTACCTACTTTGAAAATAAACTCTGGTTTGATTTTGTTGAAAATCCCAGT**

**GTTATTGGTGTTTATCATAAACTTGGAGAACGTGTACGCCAAGCTATCTTAAACACTGTT**

**AAATTTTGTGACCACATGGTCAAGGCTGGTTTAGTCGGTGTGCTCACACTAGACAACCAG**

**GACCTTAATGGCAAGTGGTATGATTTTGGTGACTTCGTAATCACTCAACCTGGTTCAGGA**

**GTAGCTATAGTTGATAGCTACTATTCTTATTTGATGCCTGTGCTCTCAATGACCGATTGT**

**CTGGCCGCTGAGACACATAGGGATTGTGATTTTAATAAACCACTCATTGAGTGGCCACTT**

**ACTGAGTATGATTTTACTGATTATAAGGTACAACTCTTTGAGAAGTACTTTAAATATTGG**

**GATCAGACGTATCACGCAAATTGCGTTAATTGTACTGATGACCGTTGTGTGTTACATTGT**

**GCTAATTTCAATGTATTGTTTGCTATGACCATGCCTAAGACTTGTTTCGGACCCATAGTC**

**CGAAAGATCTTTGTTGATGGCGTGCCATTTGTAGTATCTTGTGGTTATCACTACAAAGAA**

**TTAGGTTTAGTCATGAATATGGATGTTAGTCTCCATAGACATAGGCTCTCTCTTAAGGAG**

**TTGATGATGTATGCCGCTGATCCAGCCATGCACATTGCCTCCTCTAACGCTTTTCTTGAT**

**TTGAGGACATCATGTTTTAGTGTCGCTGCACTTACAACTGGTTTGACTTTTCAAACTGTG**

**CGGCCTGGCAATTTTAACCAAGACTTCTATGATTTCGTGGTATCTAAAGGTTTCTTTAAG**

**GAGGGCTCTTCAGTGACGCTCAAACATTTTTTCTTTGCTCAAGATGGTAATGCTGCTATT**

**ACAGATTATAATTACTATTCTTATAATCTGCCTACTATGTGTGACATCAAACAAATGTTG**

**TTCTGCATGGAAGTTGTAAACAAGTACTTCGAAATCTATGACGGTGGTTGTCTTAATGCT**

**TCTGAAGTGGTTGTTAATAATTTAGACAAGAGTGCTGGCCATCCTTTTAATAAGTTTGGC**

**AAAGCTCGTGTCTATTATGAGAGCATGTCTTACCAGGAGCAAGATGAACTCTTTGCCATG**

**ACAAAGCGTAACGTCATTCCTACCATGACTCAAATGAATCTAAAATATGCTATTAGTGCT**

**AAGAATAGAGCTCGCACTGTTGCAGGCGTGTCCATACTTAGCACAATGACTAATCGCCAG**

**TACCATCAGAAAATGCTTAAGTCCATGGCTGCAACTCGTGGAGCGACTTGCGTCATTGGT**

**ACTACAAAGTTCTATGGTGGCTGGGATTTCATGCTTAAAACATTGTACAAAGATGTTGAT**

**AATCCGCATCTTATGGGTTGGGATTACCCTAAGTGTGATAGAGCTATGCCTAATATGTGT**

**AGAATCTTCGCTTCACTCATATTAGCTCGTAAACATGGCACTTGTTGTACTACAAGGGAC**

**AGATTTTATCGCTTGGCAAATGAGTGTGCTCAGGTGCTAAGCGAATATGTTCTATGTGGT**

**GGTGGTTACTACGTCAAACCTGGAGGTACCAGTAGCGGAGATGCCACCACTGCATATGCC**

**AATAGTGTCTTTAACATTTTGCAGGCGACAACTGCTAATGTCAGTGCACTTATGGGTGCT**

**AATGGCAACAAGATTGTTGACAAAGAAGTTAAAGACATGCAGTTTGATTTGTATGTCAAT**

**GTTTACAGGAGCACTAGCCCAGACCCCAAATTTGTTGATAAATACTATGCTTTTCTTAAT**

**AAGCACTTTTCTATGATGATACTGTCTGATGACGGTGTCGTTTGCTATAATAGTGATTAT**

**GCAGCTAAGGGTTACATTGCTGGAATACAGAATTTCAAGGAAACGCTGTATTATCAGAAC**

**AATGTCTTTATGTCTGAAGCTAAATGCTGGGTGGAAACCGATCTGAAGAAAGGGCCACAT**

**GAATTCTGTTCACAGCATACGCTTTATATTAAGGATGGCGACGATGGTTACTTCCTTCCT**

**TATCCAGACCCTTCAAGAATTTTGTCTGCCGGTTGCTTTGTAGATGATATCGTTAAGACT**

**GACGGTACACTCATGGTAGAGCGGTTTGTGTCTTTGGCTATAGATGCTTACCCTCTCACA**

**AAGCATGAAGATATAGAATACCAGAATGTATTCTGGGTCTACTTACAGTATATAGAAAAA**

**CTGTATAAAGACCTTACAGGACACATGCTTGACAGTTATTCTGTCATGCTATGTGGTGAT**

**AATTCTGCTAAGTTTTGGGAAGAGGCATTCTATAGAGATCTCTATAGTTCGCCTACCACT**

**TTGCAGGCTGTCGGTTCATGCGTTGTATGCCATTCACAGACTTCCCTACGCTGTGGGACA**

**TGCATCCGTAGACCATTTCTCTGCTGTAAATGCTGCTATGATCATGTTATAGCAACTCCA**

**CATAAGATGGTTTTGTCTGTTTCTCCTTACGTTTGTAATGCCCCTGGTTGTGGCGTTTCA**

**GACGTTACTAAGCTATATTTAGGTGGTATGAGCTACTTTTGTGTAGATCATAGACCTGTG**

**TGTAGTTTTCCACTTTGCGCTAATGGTCTTGTATTCGGCTTATACAAGAATATGTGCACA**

**GGTAGTCCTTCTATAGTTGAATTTAATAGGTTGGCTACCTGTGACTGGACTGAAAGTGGT**

**GATTACACCCTTGCCAATACTACAACAGAACCACTCAAACTTTTTGCTGCTGAGACTTTA**

**CGTGCCACTGAAGAGGCGTCTAAGCAGTCTTATGCTATTGCCACCATCAAAGAAATTGTT**

**GGTGAGCGCCAACTATTACTTGTGTGGGAGGCTGGCAAGTCCAAACCACCACTCAATCGT**

**AATTATGTTTTTACTGGTTATCATATAACCAAAAATAGTAAAGTGCAGCTCGGTGAGTAC**

**ATCTTCGAGCGCATTGATTATAGTGATGCTGTATCCTACAAGTCTAGTACAACGTATAAA**

**CTGACTGTAGGTGACATCTTCGTACTTACCTCTCACTCTGTGGCTACCTTGACGGCGCCC**

**ACAATTGTGAATCAAGAGAGGTATGTTAAAATTACTGGGTTGTACCCAACCATTACGGTA**

**CCTGAAGAGTTCGCAAGTCATGTTGCCAACTTCCAAAAATCAGGTTATAGTAAATATGTC**

**ACTGTTCAGGGACCACCTGGCACTGGCAAAAGTCATTTTGCTATAGGGTTAGCGATTTAC**

**TACCCTACAGCACGTGTTGTTTATACAGCATGTTCACACGCAGCTGTTGATGCTTTGTGT**

**GAAAAAGCTTTTAAATATTTGAACATTGCTAAATGTTCCCGTATCATTCCTGCAAAGGCA**

**CGTGTTGAGTGCTATGACAGGTTTAAAGTTAATGAGACAAATTCTCAATATTTGTTTAGT**

**ACTATTAATGCTCTACCAGAAACTTCTGCCGATATTCTGGTGGTTGATGAGGTTAGTATG**

**TGCACTAATTATGATCTTTCAATTATTAATGCACGTATTAAAGCTAAGCACATTGTCTAT**

**GTAGGAGATCCAGCACAGTTGCCAGCTCCTAGGACTTTGTTGACTAGAGGCACATTGGAA**

**CCAGAAAATTTCAATAGTGTCACTAGATTGATGTGTAACTTAGGTCCTGACATATTTTTA**

**AGTATGTGCTACAGGTGTCCTAAGGAAATAGTAAGCACTGTGAGCGCTCTTGTCTACAAT**

**AATAAATTGTTAGCCAAGAAGGAGCTTTCAGGCCAGTGCTTTAAAATACTCTATAAGGGC**

**AATGTGACGCATGATGCTAGCTCTGCCATTAATAGACCACAACTCACATTTGTGAAGAAT**

**TTTATTACTGCCAATCCGGCATGGAGTAAGGCAGTCTTTATTTCGCCTTATAATTCACAG**

**AATGCTGTGGCTCGTTCAATGCTGGGTCTTACTACTCAGACTGTTGATTCCTCACAGGGT**

**TCAGAATACCAGTACGTTATCTTCTGTCAAACAGCAGATACGGCACATGCTAACAACATT**

**AACAGATTTAATGTTGCAATCACTCGTGCCCAAAAAGGTATTCTTTGTGTTATGACATCT**

**CAGGCACTCTTTGAGTCCTTAGAGTTTACTGAATTGTCTTTTACTAATTACAAGCTCCAG**

**TCTCAGATTGTAACTGGCCTTTTTAAAGATTGCTCTAGAGAAACTTCTGGCCTCTCACCT**

**GCTTATGCACCAACATACGTTAGTGTTGATGACAAGTATAAGACGAGTGATGAGCTTTGC**

**GTGAATCTTAATTTACCCGCAAATGTCCCATACTCTCGTGTTATTTCCAGGATGGGCTTT**

**AAACTCGATGCAACAGTTCCTGGATATCCTAAGCTTTTCATTACTCGTGAAGAGGCTGTA**

**AGGCAAGTTCGAAGCTGGATAGGCTTCGATGTTGAGGGTGCTCATGCTTCCCGTAATGCA**

**TGTGGCACCAATGTGCCTCTACAATTAGGATTTTCAACTGGTGTGAACTTTGTTGTTCAG**

**CCAGTTGGTGTTGTAGACACTGAGTGGGGTAACATGTTAACGGGCATTGCTGCCCGTCCT**

**CCACCAGGTGAACAGTTTAAGCACCTCGTGCCTCTTATGCATAAGGGGGCTGCGTGGCCT**

**ATTGTTAGACGACGTATAGTGCAAATGTTGTCAGACACTTTAGACAAATTGTCTGATTAC**

**TGTACGTTTGTTTGTTGGGCTCATGGCTTTGAATTAACGTCTGCATCATACTTTTGCAAG**

**ATAGGTAAGGAACAGAAGTGTTGCATGTGCAATAGACGCGCTGCAGCGTACTCTTCACCT**

**CTGCAATCTTATGCCTGCTGGACTCATTCCTGCGGTTATGATTATGTCTACAACCCTTTC**

**TTTGTCGATGTTCAACAGTGGGGTTATGTAGGCAATCTTGCTACTAATCACGATCGTTAT**

**TGCTCTGTCCATCAAGGAGCTCATGTGGCTTCTAATGATGCAATAATGACTCGTTGTTTA**

**GCTATTCATTCTTGTTTTATAGAACGTGTGGATTGGGATATAGAGTATCCTTATATCTCA**

**CATGAAAAGAAATTGAATTCCTGTTGTAGAATCGTTGAGCGCAACGTCGTACGTGCTGCT**

**CTTCTTGCCGGTTCATTTGACAAAGTCTATGATATTGGCAATCCTAAAGGAATTCCTATT**

**GTTGATGACCCTGTGGTTGATTGGCATTATTTTGATGCACAGCCCTTGACCAGAAAGGTA**

**CAACAGCTTTTCTATACAGAGGACATGGCCTCAAGATTTGCTGATGGGCTCTGCTTATTT**

**TGGAACTGTAATGTACCAAAATATCCTAATAATGCAATTGTATGCAGGTTTGACACACGT**

**GTGCATTCTGAGTTCAATTTGCCAGGTTGTGATGGCGGTAGTTTGTATGTTAACAAGCAC**

**GCTTTTCATACACCAGCATATGATGTGAGTGCATTCCGTGATCTGAAACCTTTACCATTC**

**TTTTATTATTCTACTACACCATGTGAAGTGCATGGTAATGGTAGTATGATAGAGGATATT**

**GATTATGTACCCCTAAAATCTGCAGTCTGTATTACAGCTTGTAATTTAGGGGGCGCTGTT**

**TGTAGGAAGCATGCTACAGAGTACAGAGAGTATATGGAAGCATATAATCTTGTCTCTGCA**

**TCAGGTTTCCGCCTTTGGTGTTATAAGACCTTTGATATTTATAATCTCTGGTCTACTTTT**

**ACAAAAGTTCAAGGTTTGGAAAACATTGCTTTTAATTTTGTTAAACAAGGCCATTTTATT**

**GGTGTTGAGGGTGAACTACCTGTAGCTGTAGTCAATGATAAGATCTTCACCAAGAGTGGC**

**GTTAATGACATTTGTATGTTTGAGAATAAAACCACTTTGCCTACTAATATAGCTTTTGAA**

**CTCTATGCTAAGCGTGCTGTACGCTCGCATCCCGATTTCAAATTGCTACACAATTTACAA**

**GCAGACATTTGCTACAAGTTCGTCCTTTGGGATTATGAACGTAGCAATATTTATGGTACT**

**GCTACTATTGGTGTATGTAAGTACACTGATATTGATGTTAATTCAGCTTTGAATATATGT**

**TTTGACATACGCGATAATGGTTCATTGGAGAAGTTCATGTCTACTCCCAATGCCATCTTT**

**ATTTCTGATAGAAAAATCAAGAAATACCCTTGTATGGTAGGTCCTGATTATGCTTACTTC**

**AATGGTGCTATCATCCGTGATAGTGATGTTGTTAAACAACCAGTGAAGTTCTACTTGTAT**

**AAGAAAGTCAATAATGAGTTTATTGATCCTACTGAGTGTATTTACACTCAGAGTCGCTCT**

**TGTAGTGACTTCCTACCCCTGTCTGACATGGAGAAAGACTTTCTATCTTTTGATAGTGAT**

**GTTTTCATTAAGAAGTATGGCTTGGAAAACTATGCTTTTGAGCACGTAGTCTATGGAGAC**

**TTCTCTCATACTACGTTAGGCGGTCTTCACTTGCTTATTGGTTTATACAAGAAGCAACAG**

**GAAGGTCATATTATTATGGAAGAAATGCTAAAAGGTAGCTCAACTATTCATAACTATTTT**

**ATTACTGAGACTAACACAGCGGCTTTTAAGGCGGTGTGTTCTGTTATAGATTTAAAGCTT**

**GACGACTTTGTTATGATTTTAAAGAGTCAAGACCTTGGCGTAGTATCCAAGGTTGTCAAG**

**GTTCCTATTGACTTAACAATGATTGAGTTTATGTTATGGTGTAAGGATGGACAGGTTCAA**

**ACCTTCTACCCTCGACTCCAGGCTTCTGCAGATTGGAAACCTGGTCATGCAATGCCATCC**

**CTCTTTAAAGTTCAAAATGTAAACCTTGAACGTTGTGAGCTTGCTAATTACAAGCAATCT**

**ATTCCTATGCCTCGCGGTGTGCACATGAACATCGCTAAATATATGCAATTGTGCCAGTAT**

**TTAAATACTTGCACATTAGCCGTGCCTGCCAATATGCGTGTTATACATTTTGGCGCTGGT**

**TCTGATAAAGGTATCGCTCCTGGTACATCAGTTTTACGACAGTGGCTTCCTACAGATGCC**

**ATTATTATAGATAATGATTTAAATGAGTTCGTGTCAGATGCTGACATAACTTTATTTGGA**

**GATTGTGTAACTGTACGTGTCGGCCAACAAGTGGATCTTGTTATTTCCGACATGTATGAT**

**CCTACTACTAAGAATGTAACAGGTAGTAATGAGTCAAAGGCTTTATTCTTTACTTACCTG**

**TGTAACCTCATTAATAATAATCTTGCTCTTGGTGGGTCTGTTGCTATTAAAATAACAGAA**

**CACTCTTGGAGCGTTGAACTTTATGAACTTATGGGAAAATTTGCTTGGTGGACTGTTTTC**

**TGCACCAATGCAAATGCATCCTCATCTGAAGGATTCCTCTTAGGTATTAATTACTTGGGT**

**ACTATTAAAGAAAATATAGATGGTGGTGCTATGCACGCCAACTATATATTTTGGAGAAAT**

**TCCACTCCTATGAATCTGAGTACTTACTCACTTTTTGATTTATCCAAGTTTCAATTAAAA**

**TTAAAAGGAACACCAGTTCTTCAATTAAAGGAGAGTCAAATTAACGAACTCGTAATATCT**

**CTCCTGTCGCAGGGTAAGTTACTTATCCGTGACAATGATACACTCAGTGTTTCTACTGAT**

**GTTCTTGTTAACACCTACAGAAAGTTACGTTGATGTAGGGCCAGATTCTGTTAAGTCTGC**

**TTGTATTGAGGTTGATATACAACAGACTTTCTTTGATAAAACTTGGCCTAGGCCAATTGA**

**TGTTTCTAAGGCTGACGGTATTATATACCCTCAAGGCCGTACATATTCTAACATAACTAT**

**CACTTATCAAGGTCTTTTTCCCTATCAGGGAGACCATGGTGATATGTATGTTTACTCTGC**

**AGGACATGCTACAGGCACAACTCCACAAAAGTTGTTTGTAGCTAACTATTCTCAGGACGT**

**CAAACAGTTTGCTAATGGGTTTGTCGTCCGTATAGGAGCAGCTGCCAATTCCACTGGCAC**

**TGTTATTATTAGCCCATCTACCAGCGCTACTATACGAAAAATTTACCCTGCTTTTATGCT**

**GGGTTCTTCAGTTGGTAATTTCTCAGATGGTAAAATGGGCCGCTTCTTCAATCATACTCT**

**AGTTCTTTTGCCCGATGGATGTGGCACTTTACTTAGAGCTTTTTATTGTATTCTAGAGCC**

**TCGCTCTGGAAATCATTGTCCTGCTGGCAATTCCTATACTTCTTTTGCCACTTATCACAC**

**TCCTGCAACAGATTGTTCTGATGGCAATTACAATCGTAATGCCAGTCTGAACTCTTTTAA**

**GGAGTATTTTAATTTACGTAACTGCACCTTTATGTACACTTATAACATTACCGAAGATGA**

**GATTTTAGAGTGGTTTGGCATTACACAAACTGCTCAAGGTGTTCACCTCTTCTCATCTCG**

**GTATGTTGATTTGTACGGCGGCAATATGTTTCAATTTGCCACCTTGCCTGTTTATGATAC**

**TATTAAGTATTATTCTATCATTCCTCACAGTATTCGTTCTATCCAAAGTGATAGAAAAGC**

**TTGGGCTGCCTTCTACGTATATAAACTTCAACCGTTAACTTTCCTGTTGGATTTTTCTGT**

**TGATGGTTATATACGCAGAGCTATAGACTGTGGTTTTAATGATTTGTCACAACTCCACTG**

**CTCATATGAATCCTTCGATGTTGAATCTGGAGTTTATTCAGTTTCGTCTTTCGAAGCAAA**

**ACCTTCTGGCTCAGTTGTGGAACAGGCTGAAGGTGTTGAATGTGATTTTTCACCTCTTCT**

**GTCTGGCACACCTCCTCAGGTTTATAATTTCAAGCGTTTGGTTTTTACCAATTGCAATTA**

**TAATCTTACCAAATTGCTTTCACTTTTTTCTGTGAATGATTTTACTTGTAGTCAAATATC**

**TCCAGCAGCAATTGCTAGTAACTGTTATTCTTCACTGATTTTGGATTATTTTTCATACCC**

**ACTTAGTATGAAATCCGATCTCAGTGTTAGTTCTGCTGGTCCAATATCCCAGTTTAATTA**

**TAAACAGTCCTTTTCTAATCCCACATGTTTGATTTTAGCGACTGTTCCTCATAACCTTAC**

**TACTATTACTAAGCCTCTTAAGTACAGCTATATTAACAAGTGCTCTCGTTTTCTTTCTGA**

**TGATCGTACTGAAGTACCTCAGTTAGTGAACGCTAATCAATACTCACCCTGTGTATCCAT**

**TGTCCCATCCACTGTGTGGGAAGACGGTGATTATTATAGGAAACAACTATCTCCACTTGA**

**AGGTGGTGGCTGGCTTGTTGCTAGTGGCTCAACTGTTGCCATGACTGAGCAATTACAGAT**

**GGGCTTTGGTATTACAGTTCAATATGGTACAGACACCAATAGTGTTTGCCCCAAGCTTGA**

**ATTTGCTAATGACACAAAAATTGCCTCTCAATTAGGCAATTGCGTGGAATATTCCCTCTA**

**TGGTGTTTCGGGCCGTGGTGTTTTTCAGAATTGCACAGCTGTAGGTGTTCGACAGCAGCG**

**CTTTGTTTATGATGCGTACCAGAATTTAGTTGGCTATTATTCTGATGATGGCAACTACTA**

**CTGTTTGCGTGCTTGTGTTAGTGTTCCTGTTTCTGTCATCTATGATAAAGAAACTAAAAC**

**CCACGCTACTCTATTTGGTAGTGTTGCATGTGAACACATTTCCTCTACCATGTCTCAATA**

**CTCCCGTTCTACGCGATCAATGCTTAAACGGCGAGATTCTACATATGGCCCCCTTCAGAC**

**ACCTGTTGGTTGTGTCCTAGGACTTGTTAATTCCTCTTTGTTCGTAGAGGACTGCAAGTT**

**GCCTCTTGGTCAATCTCTCTGTGCTCTTCCTGACACACCTAGTACTCTCACACCTCGCAG**

**TGTGCGCTCTGTTCCAGGTGAAATGCGCTTGGCATCCATTGCTTTTAATCATCCTATTCA**

**GGTTGATCAACTTAATAGTAGTTATTTTAAATTAAGTATACCCACTAATTTTTCCTTTGG**

**TGTGACTCAGGAGTACATTCAGACAACCATTCAGAAAGTTACTGTTGATTGTAAACAGTA**

**CGTTTGCAATGGTTTCCAGAAGTGTGAGCAATTACTGCGCGAGTATGGCCAGTTTTGTTC**

**CAAAATAAACCAGGCTCTCCATGGTGCCAATTTACGCCAGGATGATTCTGTACGTAATTT**

**GTTTGCGAGCGTGAAAAGCTCTCAATCATCTCCTATCATACCAGGTTTTGGAGGTGACTT**

**TAATTTGACACTTCTAGAACCTGTTTCTATATCTACTGGCAGTCGTAGTGCACGTAGTGC**

**TATTGAGGATTTGCTATTTGACAAAGTCACTATAGCTGATCCTGGTTATATGCAAGGTTA**

**CGATGATTGCATGCAGCAAGGTCCAGCATCAGCTCGTGATCTTATTTGTGCTCAATATGT**

**GGCTGGTTACAAAGTATTACCTCCTCTTATGGATGTTAATATGGAAGCCGCGTATACCTC**

**ATCTTTGCTTGGCAGCATAGCAGGTGTTGGCTGGACTGCTGGCTTATCCTCCTTTGCTGC**

**TATTCCATTTGCACAGAGTATCTTTTATAGGTTAAACGGTGTTGGCATTACTCAACAGGT**

**TCTTTCAGAGAACCAAAAGCTTATTGCCAATAAGTTTAATCAGGCTCTGGGAGCTATGCA**

**AACAGGCTTCACTACAACTAATGAAGCTTTTCACAAGGTTCAGGATGCTGTGAACAACAA**

**TGCACAGGCTCTATCCAAATTAGCTAGCGAGCTATCTAATACTTTTGGTGCTATTTCCGC**

**CTCTATTGGAGACATCATACAACGTCTTGATGTTCTCGAACAGGACGCCCAAATAGACAG**

**ACTTATTAATGGCCGTTTGACAACACTAAATGCTTTTGTTGCACAGCAGCTTGTTCGTTC**

**CGAATCAGCTGCTCTTTCGGCTCAATTGGCTAAAGATAAAGTCAATGAGTGTGTCAAGGC**

**ACAATCCAAGCGTTCTGGATTTTGCGGTCAAGGCACACATATAGTGTCCTTTGTTGTAAA**

**TGCCCCTAATGGCCTTTACTTCATGCATGTTGGTTATTACCCTAGCAACCACATTGAGGT**

**TGTTTCTGCTTATGGTCTTTGCGATGCAGCTAACCCTACTAATTGTATAGCCCCTGTTAA**

**TGGCTACTTTATTAAAACTAATAACACTAGGATTGTTGATGAGTGGTCATATACTGGCTC**

**GTCCTTCTATGCACCTGAGCCCATCACCTCTCTTAATACTAAGTATGTTGCACCACAGGT**

**GACATACCAAAACATTTCTACTAACCTCCCTCCTCCTCTTCTCGGCAATTCCACCGGGAT**

**TGACTTCCAAGATGAGTTGGATGAGTTTTTCAAAAATGTTAGCACCAGTATACCTAATTT**

**TGGTTCTCTAACACAGATTAATACTACATTACTCGATCTTACCTACGAGATGTTGTCTCT**

**TCAACAAGTTGTTAAAGCCCTTAATGAGTCTTACATAGACCTTAAAGAGCTTGGCAATTA**

**TACTTATTACAACAAATGGCCGTGGTACATTTGGCTTGGTTTCATTGCTGGGCTTGTTGC**

**CTTAGCTCTATGCGTCTTCTTCATACTGTGCTGCACTGGTTGTGGCACAAACTGTATGGG**

**AAAACTTAAGTGTAATCGTTGTTGTGATAGATACGAGGAATACGACCTCGAGCCGCATAA**

**GGTTCATGTTCACTAATTAACGAACTATCAATGAGAGTTCAAAGACCACCCACTCTCTTG**

**TTAGTGTTCTCACTCTCTCTTTTGGTCACTGCATTTTCAAAACCTCTCTATGTACCTGAG**

**CATTGTCAGAATTATTCTGGTTGCATGCTTAGGGCTTGTATTAAAACTGCCCAAGCTGAT**

**ACAGCTGGTCTTTATACAAATTTTCGAATTGACGTCCCATCTGCAGAATCAACTGGTACT**

**CAATCAGTTTCTGTCGATCGTGAGTCAACTTCAACTCATGATGGTCCTACCGAACATGTT**

**ACTAGTGTGAATCTTTTTGACGTTGGTTACTCAGTTAATTAACGAACTCTATGGATTACG**

**TGTCTCTGCTTAATCAAATTTGGCAGAAGTACCTTAATTCACCGTATACTACTTGTTTGT**

**ATATCCCTAAACCCACAGCTAAGTATACACCTTTAGTTGGCACTTCATTGCACCCTGTGC**

**TGTGGAACTGTCAGCTATCCTTTGCTGGTTATACTGAATCTGCTGTTAATTCTACAAAAG**

**CTTTGGCCAAACAGGACGCAGCTCAGCGAATCGCTTGGTTGCTACATAAGGATGGAGGAA**

**TCCCTGATGGATGTTCCCTCTACCTCCGGCACTCAAGTTTATTCGCGCAAAGCGAGGAAG**

**AGGAGTCATTCTCCAACTAAGAAACTGCGCTACGTTAAGCGTAGATTTTCTCTTCTGCGC**

**CCTGAAGACCTTAGTGTTATTGTCCAACCAACACACTATGTCAGGGTTACATTTTCAGAC**

**CCCAACATGTGGTATCTACGTTCGGGTCATCATTTACACTCAGTTCACAATTGGCTTAAA**

**CCTTATGGCGGCCAACCTGTTTCTGAGTACCATATTACTCTAGCTTTGCTAAATCTCACT**

**GATGAAGATTTAGCTAGAGATTTTTCACCCATTGCGCTCTTTTTGCGCAATGTCAGATTT**

**GAGCTACATGAGTTCGCCTTGCTGCGCAAAACTCTTGTTCTTAATGCATCAGAGATCTAC**

**TGTGCTAACATACATAGATTTAAGCCTGTGTATAGAGTTAACACGGCAATCCCTACTATT**

**AAGGATTGGCTTCTCGTTCAGGGATTTTCCCTTTACCATAGTGGCCTCCCTTTACATATG**

**TCAATCTCTAAATTGCATGCACTGGATGATGTTACTCGCAATTACATCATTACAATGCCA**

**TGCTTTAGAACTTATCCTCAACAAATGTTTGTTACTCCTTTGGCCGTAGATGTTGTCTCC**

**ATACGGTCTTCCAATCAGGGTAATAAACAAATTGTTCATTCTTACCCCATTTTACATCAT**

**CCAGGATTTTAACGAACTATGGCTTTCTCGGCGTCTTTATTTAAACCCGTCCAGCTAGTC**

**CCAGTTTCTCCTGCATTTCATCGCATTGAGTCTACTGACTCTATTGTTTTCACATACATT**

**CCTGCTAGCGGCTATGTAGCTGCTTTAGCTGTCAATGTGTGTCTCATTCCCCTATTATTA**

**CTGCTACGTCAAGATACTTGTCGTCGCAGCATTATCAGAACTATGGTTCTCTATTTCCTT**

**GTTCTGTATAACTTTTTATTAGCCATTGTACTAGTCAATGGTGTACATTATCCAACTGGA**

**AGTTGCCTGATAGCCTTCTTAGTTATCCTCATAATACTTTGGTTTGTAGATAGAATTCGT**

**TTCTGTCTCATGCTGAATTCCTACATTCCACTGTTTGACATGCGTTCTCACTTTATTCGT**

**GTTAGTACAGTTTCTTCTCATGGTATGGTCCCTGTCATACACACCAAACCATTATTTATT**

**AGAAACTTCGATCAGCGTTGCAGCTGTTCTCGTTGTTTTTATTTGCACTCTTCCACTTAT**

**ATAGAGTGCACTTATATTAGCCGTTTTAGTAAGATTAGCCTAGTTTCTGTAACTGACTTC**

**TCCTTAAACGGCAATGTTTCCACTGTTTTCGTGCCTGCAACGCGCGATTCAGTTCCTCTT**

**CACATAATCGCCCCGAGCTCGCTTATCGTTTAAGCAGCTCTGCGCTACTATGGGTCCCGT**

**GTAGAGGCTAATCCATTAGTCTCTCTTTGGACATATGGAAAACGAACTATGTTACCCTTT**

**GTCCAAGAACGAATAGGGTTGTTCATAGTAAACTTTTTCATTTTTACCGTAGTATGTGCT**

**ATAACACTCTTGGTGTGTATGGCTTTCCTTACGGCTACTAGATTATGTGTGCAATGTATG**

**ACAGGCTTCAATACCCTGTTAGTTCAGCCCGCATTATACTTGTATAATACTGGACGTTCA**

**GTCTATGTAAAATTCCAGGATAGTAAACCCCCTCTACCACCTGACGAGTGGGTTTAACGA**

**ACTCCTTCATAATGTCTAATATGACGCAACTCACTGAGGCGCAGATTATTGCCATTATTA**

**AAGACTGGAACTTTGCATGGTCCCTGATCTTTCTCTTAATTACTATCGTACTACAGTATG**

**GATACCCATCCCGTAGTATGACTGTCTATGTCTTTAAAATGTTTGTTTTATGGCTCCTAT**

**GGCCATCTTCCATGGCGCTATCAATATTTAGCGCCGTTTATCCAATTGATCTAGCTTCCC**

**AGATAATCTCTGGCATTGTAGCAGCTGTTTCAGCTATGATGTGGATTTCCTACTTTGTGC**

**AGAGTATCCGGCTGTTTATGAGAACTGGATCATGGTGGTCATTCAATCCTGAGACTAATT**

**GCCTTTTGAACGTTCCATTTGGTGGTACAACTGTCGTACGTCCACTCGTAGAGGACTCTA**

**CCAGTGTAACTGCTGTTGTAACCAATGGCCACCTCAAAATGGCTGGCATGCATTTCGGTG**

**CTTGTGACTACGACAGACTTCCTAATGAAGTCACCGTGGCCAAACCCAATGTGCTGATTG**

**CTTTAAAAATGGTGAAGCGGCAAAGCTACGGAACTAATTCCGGCGTTGCCATTTACCATA**

**GATATAAGGCAGGTAATTACAGGAGTCCGCCTATTACGGCGGATATTGAACTTGCATTGC**

**TTCGAGCTTAGGCTCTTTAGTAAGAGTATCTTAATTGATTTTAACGAATCTCAATTTCAT**

**TGTTATGGCATCCCCTGCTGCACCTCGTGCTGTTTCCTTTGCCGATAACAATGATATAAC**

**AAATACAAACCTGTCTCGAGGTAGAGGACGTAATCCAAAACCACGAGCTGCACCAAATAA**

**CACTGTCTCTTGGTACACTGGGCTTACCCAACACGGGAAAGTCCCTCTTACCTTTCCACC**

**TGGGCAGGGTGTACCTCTTAATGCCAATTCCACCCCTGCGCAAAATGCTGGGTATTGGCG**

**GAGACAGGACAGAAAAATTAATACCGGGAATGGAATTAAGCAACTGGCTCCCAGGTGGTA**

**CTTCTACTACACTGGAACTGGACCCGAAGCAGCACTCCCATTCCGGGCTGTTAAGGATGG**

**CATCGTTTGGGTCCATGAAGATGGCGCCACTGATGCTCCTTCAACTTTTGGGACGCGGAA**

**CCCTAACAATGATTCAGCTATTGTTACACAATTCGCGCCCGGTACTAAGCTTCCTAAAAA**

**CTTCCACATTGAGGGGACTGGAGGCAATAGTCAATCATCTTCAAGAGCCTCTAGCGTAAG**

**CAGAAACTCTTCCAGATCTAGTTCACAAGGTTCAAGATCAGGAAACTCTACCCGCGGCAC**

**TTCTCCAGGTCCATCTGGAATCGGAGCAGTAGGAGGTGATCTACTTTACCTTGATCTTCT**

**GAACAGACTACAAGCCCTTGAGTCTGGCAAAGTAAAGCAATCGCAGCCAAAAGTAATCAC**

**TAAGAAAGATGCTGCTGCTGCTAAAAATAAGATGCGCCACAAGCGCACTTCCACCAAAAG**

**TTTCAACATGGTGCAAGCTTTTGGTCTTCGCGGACCAGGAGACCTCCAGGGAAACTTTGG**

**TGATCTTCAATTGAATAAACTCGGCACTGAGGACCCACGTTGGCCCCAAATTGCTGAGCT**

**TGCTCCTACAGCCAGTGCTTTTATGGGTATGTCGCAATTTAAACTTACCCATCAGAACAA**

**TGATGATCATGGCAACCCTGTGTACTTCCTTCGGTACAGTGGAGCCATTAAACTTGACCC**

**AAAGAATCCCAACTACAATAAGTGGTTGGAGCTTCTTGAGCAAAATATTGATGCCTACAA**

**AACCTTCCCTAAGAAGGAAAAGAAACAAAAGGCACCAAAAGAAGAATCAACAGACCAAAT**

**GTCTGAACCTCCTAAGGAGCATCGTGTGCAAGGTACTCAGCGCACTCGCACCCGTCCAAG**

**TGTTCAGCCTGGTCCAATGATTGATGTTAACACTGATTAGTGTCACTCAAAGTAACAAGA**

**TCGCGGCAATCGTTTGTGTTTGGTAACCCCATCTCACCATCGCTTGTCCACTCTTGCACA**

**GAATGGAATCATGTTGTAATTACAGTGCAATAAGGTAATTATAACCCATTTAATTGATAG**

**CTATGCTTTATTAAAGTGTGTAGCTGTAGAGAGAATGTTAAAGACTGTCACCTCTGCGTG**

**ATTGCAAGTGAACAGTGCCCCCCGGGAAGAGCTCTACAGTGTGAAATGTAAATAAAAATA**

**GCTATTATTCAATTAGATTAGGCTAATTAGATGATTTGCAAAAAAAAAAAA**
